# Associations between community built environment and leisure-time physical activity among older adults: a systematic review and meta-analysis

**DOI:** 10.3389/fpubh.2026.1956100

**Published:** 2026-09-29

**Authors:** Haixin Li, Liping Zhong

**Affiliations:** School of Physical Education, Hunan University of Technology, Zhuzhou, Hunan, China

**Keywords:** community built environment, leisure-time physical activity, leisure-time walking, older adults, systematic review, three-level meta-analysis

## Abstract

**Objective:**

To systematically evaluate associations between the community built environment and leisure-time physical activity (LTPA) among older adults using three-level meta-analysis to account for dependent effect estimates within studies.

**Methods:**

PubMed, Embase, Web of Science, Scopus, the Cochrane Library, and SPORTDiscus were searched for cross-sectional studies. Built-environment exposures were grouped into nine domains, and LTPA outcomes into leisure-time walking (LTW), neighbourhood leisure-time walking (NLTW), and General LTPA. Odds ratios (ORs) with 95% confidence intervals (CIs) were classified as categorical contrast (CAT) or continuous unit (UNIT) effects and pooled separately using three-level random-effects models. Cluster-robust variance estimation with CR2 correction, heterogeneity and prediction intervals, subgroup analyses, meta-regression, publication-bias assessments, and sensitivity analyses were conducted.

**Results:**

Thirty-four studies contributed 344 CAT and UNIT effect estimates. In primary CAT analyses, more favourable built environments were associated with higher LTW (OR = 1.103, 95% CI 1.022–1.191) and General LTPA (OR = 1.180, 95% CI 1.089–1.278), with both associations remaining significant after CR2 correction. NLTW was not significantly associated with the overall built environment. In UNIT analyses, the positive association with NLTW lost significance after CR2 correction, while no consistent associations were observed for LTW or General LTPA. Prediction intervals for all three CAT models crossed OR = 1, indicating substantial contextual variation. Domain-specific analyses showed relatively consistent positive associations between aesthetics and LTW, parks and green spaces and General LTPA, and destination accessibility and LTW in UNIT analyses. Sensitivity analyses generally supported the LTW and General LTPA CAT findings.

**Conclusion:**

More favourable community built environments were associated with higher LTW and General LTPA among older adults, but associations varied by environmental attribute, activity type, and study context. Because the NLTW analyses were based on very few independent study samples, the NLTW findings should be regarded as hypothesis-generating rather than as established effects. Because all included studies were cross-sectional and some analyses showed substantial heterogeneity or limited study numbers, the findings should be interpreted as associations rather than causal effects. Longitudinal studies and natural experiments with refined exposure assessment are needed.

**Systematic review registration:**

https://www.crd.york.ac.uk/PROSPERO/view/CRD420261435582, Unique Identifier: CRD420261435582.

## 1 Introduction

Global population ageing is accelerating, accompanied by a continuing increase in the number of older adults and growing concerns regarding chronic disease prevention, maintenance of functional capacity, and healthy ageing. The World Health Organization (WHO) has projected that the global population aged 60 years and older will increase from 1 billion in 2020 to 1.4 billion by 2030 and is expected to reach 2.1 billion by 2050 (1). WHO further notes that the United Nations Decade of Healthy Ageing (2021–2030) defines older people as individuals aged 60 years and above, whereas the WHO Guidelines on Physical Activity and Sedentary Behaviour primarily use an age threshold of 65 years and above for recommendations specific to older adults. Accordingly, the present study adopted ≥60 years as the criterion for defining older adults, consistent with the healthy ageing framework (2). A global pooled analysis of 507 population-based surveys involving approximately 5.7 million adults from 163 countries and territories showed that 31.3% of adults were insufficiently physically active in 2022, with the prevalence of insufficient physical activity increasing with age among adults aged 60 years and older across all regions and in both sexes (3). Regular physical activity is associated with a reduced risk of all-cause mortality, cardiovascular disease, and type 2 diabetes, while also providing benefits for mental health, cognitive function, and physical functioning (4). Physical activity occurs across several domains, including leisure, transportation, occupation, and household activities; the present study focuses specifically on leisure-time physical activity (LTPA). Physical activity is not determined solely by individual health status or behavioural intention. According to the social ecological model, physical activity is shaped by multiple levels of influence, including individual, social, physical environmental, and policy factors, and the environmental correlates of activity may differ across leisure, transportation, occupational, and household domains (5). The built environment represents a potentially modifiable environmental determinant and broadly encompasses human-made and human-modified physical surroundings, including land-use patterns, transportation systems, streets, public spaces, and related facilities. Built-environment attributes are commonly assessed using resident perceptions, environmental audits, and geographic information system (GIS)-based measures (6). The present study focuses on the community or neighbourhood built environment that older adults can access and use around their place of residence. Drawing on environmental-attribute classifications used in previous systematic reviews of the built environment and physical activity among older adults, and adapting these constructs to the prespecified coding scheme of the present review, built-environment exposures were grouped into nine analytical domains: traffic safety, destination accessibility, environmental safety, aesthetics, parks and green spaces, residential density, land-use mix, street connectivity, and sidewalk quality (7–9). As ageing is often accompanied by changes in mobility and functional capacity, older adults’ daily activities may become increasingly dependent on conditions in and around their residential neighbourhoods, making the relationship between the community built environment and LTPA particularly relevant.

Existing evidence suggests that associations between specific community built-environment attributes and LTPA among older adults are not consistent. Previous systematic reviews and meta-analyses have reported positive associations of walkability, land-use mix accessibility, and environmental aesthetics with leisure-time walking, whereas access to recreational facilities and parks or open spaces has been associated with overall LTPA; however, many combinations of environmental attributes and LTPA outcomes remain insufficiently studied (8). A subsequent umbrella review further showed that significant associations between built-environment attributes and leisure-time walking were less frequently observed than those for transport walking, overall walking, and overall physical activity, while evidence regarding leisure-time cycling was even more limited (9). Importantly, different types of LTPA are not necessarily equivalent in terms of behavioural purpose or spatial context. Previous research has distinguished leisure-time walking (LTW) without a specified location from neighbourhood leisure-time walking (NLTW) explicitly undertaken within the residential neighbourhood, indicating that these behaviours may reflect different behavioural and spatial contexts. Similarly, cycling, combined walking and cycling, and other forms of leisure-time physical activity may differ from leisure-time walking alone in their activity requirements and dependence on environmental conditions. Pooling heterogeneous LTPA outcomes may therefore obscure differences between specific environmental attributes and particular activity types. It is thus important to evaluate LTW, NLTW, and General LTPA separately.

In addition to differences in behavioural outcomes, substantial methodological heterogeneity exists across studies in the measurement of built-environment exposures, the spatial definition of neighbourhoods, and the reporting of effect estimates. The same environmental attribute may be measured using perceived assessments, GIS-derived indicators, or environmental audits, while neighbourhood boundaries and the operationalization of environmental variables also vary across studies, thereby increasing heterogeneity and complicating the synthesis of effect estimates across studies (6). Moreover, studies of the community built environment frequently report multiple environmental attributes, multiple spatial scales, or several eligible LTPA effect estimates derived from the same study sample. Consequently, a single study may contribute multiple correlated effect estimates based on overlapping participants. Conventional meta-analysis generally assumes that included effect estimates are statistically independent. Selecting only one effect estimate from each study may result in the loss of relevant information, whereas treating multiple effect estimates from the same study as independent observations may lead to inappropriate standard errors and statistical inference (10, 11). Three-level meta-analysis provides a suitable approach for such multilevel data structures because it allows multiple eligible effect estimates from the same study to be retained while accounting for within-study dependence. It further partitions total variability into sampling variance, within-study heterogeneity among effect estimates, and between-study heterogeneity (10, 11).

Accordingly, this systematic review and three-level meta-analysis aimed to systematically evaluate the associations between the community built environment and LTW, NLTW, and General LTPA among adults aged 60 years and older, and to compare effect estimates across different built-environment domains, measurement approaches, and study characteristics. By distinguishing LTPA behaviours according to activity type and behavioural context, and by applying three-level meta-analysis to account for multiple correlated effect estimates within individual studies, this review sought to provide more precise estimates of the overall associations between the community built environment and LTPA among older adults, characterise the extent and sources of heterogeneity, and identify environmental factors warranting further investigation. The findings may help inform future research on physical activity in older populations and provide evidence to support the optimization of community environments for healthy and active ageing.

## 2 Methods

This systematic review and meta-analysis was designed, conducted, and reported in accordance with the Preferred Reporting Items for Systematic Reviews and Meta-Analyses 2020 (PRISMA 2020) statement (12). The recommendations of PRISMA-S for reporting systematic literature searches were also followed to enhance the transparency, completeness, and reproducibility of the review process (13). The study protocol was prospectively registered with the International Prospective Register of Systematic Reviews (PROSPERO; registration number: CRD420261435582) on June 28, 2026. Because this review focused on observational associations between exposure to the community built environment and LTPA among older adults rather than on intervention effects, the research question was structured using the PECO framework—Population, Exposure, Comparator, and Outcome—which is appropriate for questions concerning environmental and other exposures. Study design was additionally specified as an eligibility component, yielding a PECOS structure for study selection (14, 15).

### 2.1 Search strategy and study selection

#### 2.1.1 Data sources and searches

To systematically identify studies examining the association between the community built environment and LTPA among older adults, we searched PubMed, Embase, Web of Science, Scopus, the Cochrane Library, and SPORTDiscus (via EBSCOhost). Each database was searched from inception to August 23, 2026, and only studies published in English were eligible for inclusion.

The search strategy was constructed around three main concept blocks: older adults, the community built environment, and physical activity. Search terms related to older adults included “Aged,” “older adults,” “older people,” and “elderly.” Built-environment terms included “built environment,” “community built environment,” “neighbourhood environment,” “neighborhood environment,” “walkability,” and terms related to traffic safety, destination accessibility, environmental safety, aesthetics, parks and green spaces, residential density, land-use mix, street connectivity, and sidewalks. Physical activity terms included “physical activity,” “leisure-time physical activity,” “leisure physical activity,” “recreational physical activity,” “walking,” “recreational walking,” “exercise walking,” and “ambulation.” Subject headings, free-text terms, and database-specific field tags were combined according to the indexing systems and search syntax of each database. Terms within the same concept block were combined using the Boolean operator “OR,” whereas the three concept blocks were combined using “AND.” The complete search strategies for all databases are provided in Supplementary Method S1. The search process and its reporting followed the PRISMA-S recommendation (13).

To minimise the risk of missing potentially eligible studies, the reference lists of all included studies and relevant systematic reviews and meta-analyses were also manually screened. Reviews, meta-analyses, study protocols, conference abstracts, commentaries, and editorials were not included as independent studies, but were used, where appropriate, to identify potentially eligible original studies.

During revision, the search strategy was broadened to improve sensitivity, and all potentially eligible records were re-screened using the clarified LTPA eligibility criteria. This process identified additional eligible studies, bringing the final number of included studies to 34 without broadening the predefined definition of LTPA.

#### 2.1.2 Study selection and eligibility criteria

All retrieved records were imported into reference management software. Duplicate records were first removed using automated identification procedures followed by manual verification. Two reviewers independently screened titles and abstracts according to the prespecified inclusion and exclusion criteria. Full texts were obtained for potentially eligible articles and independently assessed for eligibility by the same two reviewers. Disagreements were resolved through discussion; when consensus could not be reached, a third reviewer adjudicated. The processes of study identification, deduplication, title and abstract screening, full-text assessment, and final inclusion were documented using a PRISMA flow diagram (12).

Studies were eligible if they met the following PECOS criteria.

(1) Population: Participants were older adults as defined in the original studies, generally individuals aged 60 or 65 years and older. For studies using other age thresholds but explicitly targeting older or later-life populations, eligibility was determined according to the original study definition of the target population and the age distribution of the sample. Studies including participants from multiple age groups were eligible only when results for older adults could be separately identified and extracted.
(2) Exposure: Built-environment factors were prospectively classified into nine domains: traffic safety (TS), destination accessibility (DA), environmental safety (ES), aesthetics (AE), parks and green spaces (PG), residential density (RD), land-use mix (LUM), street connectivity (SC), and sidewalk quality (SQ). This classification was informed by environmental-attribute frameworks used in previous systematic reviews of the neighbourhood built environment and physical activity among older adults and was further adapted to the prespecified research questions and coding scheme of the present review (6–8). Built-environment exposures could be assessed objectively using geographic information systems (GIS), administrative spatial data, or environmental audits, or subjectively using perceived measures such as the Neighborhood Environment Walkability Scale (NEWS) and other survey-based instruments (6, 16).
(3) Comparator: For categorical exposures, the comparator was the reference category specified in the original study, such as a lower exposure level, poorer environmental quality, or the absence of a favourable built-environment characteristic. For continuous exposures, the comparison was defined according to the unit increment reported in the original study, for example, per one-unit increase, a specified distance, a specified density increment, or another clearly defined continuous change. Because built-environment variables varied substantially in their measurement units and exposure contrasts, no common exposure increment was imposed. Instead, the original reference category, exposure contrast, and unit increment for continuous variables were recorded in full during data extraction and were used to assess the comparability of effect estimates.
(4) Outcome: Studies were required to report LTPA among older adults or to provide physical activity outcomes that could be clearly identified as occurring in the leisure domain based on the description of activity purpose, activity type, and behavioural context in the original article. Drawing on outcome classifications used in previous systematic reviews of the built environment and LTPA among older adults, eligible outcomes were consolidated for the present review into three analytical categories: LTW, NLTW, and General LTPA (8, 9).

The first category was leisure-time walking (LTW), defined as walking undertaken for leisure, recreation, exercise, or sport without an explicit requirement that the activity occur within the participant’s residential neighbourhood or community.

The second category was neighbourhood leisure-time walking (NLTW), defined as leisure, recreational, or exercise walking that the original study explicitly specified as occurring within the residential neighbourhood, local community, or a corresponding neighbourhood area.

The third category was General LTPA, comprising other eligible leisure-time physical activity outcomes not classified as LTW or NLTW. These included leisure-time cycling, combined leisure-time walking and cycling, overall LTPA comprising multiple types of leisure-time physical activity, and other leisure-time physical activity outcomes explicitly excluding walking (8, 9). General LTPA was restricted to physical activity explicitly undertaken within the leisure domain and did not include total physical activity or physical activity undertaken primarily for transportation, occupational, or household purposes.

Separate datasets were constructed for the three outcome categories, and each category was meta-analysed independently. Effect estimates from different LTPA categories were not pooled into a single overall effect. Studies reporting only transport-related physical activity, occupational physical activity, total physical activity, or sedentary behaviour were not included in the corresponding analyses when an LTPA outcome meeting the present definitions could not be separately identified or extracted.

For quantitative synthesis, studies were required to report an odds ratio (OR) with a 95% confidence interval (CI), or sufficient information to derive a corresponding ratio measure of association and its sampling variance. When multiple adjusted models were reported within the same study, the effect estimate from the model with the most comprehensive adjustment for relevant confounders and the greatest consistency with the research question of the present review was preferentially extracted.

(5) Study design: Eligible studies were original analytical cross-sectional studies examining associations between the community built environment and LTPA among older adults and providing effect estimates suitable for the quantitative synthesis. Systematic reviews, meta-analyses, study protocols, conference abstracts, commentaries, editorials, case reports, dissertations, and other non-original publications were excluded.

For multiple publications derived from the same survey, data source, or substantially overlapping samples, overlap was assessed by comparing the data source, study location, survey period, and sample characteristics. Completely duplicated effect estimates were retained only once. When separate publications based on the same underlying sample reported distinct and non-duplicated built-environment exposures or LTPA outcomes, the relevant effect estimates were retained. Multiple effect estimates originating from the same independent study sample were treated as correlated effects in the statistical analyses.

### 2.2 Data extraction and quality assessment

#### 2.2.1 Data extraction

Two reviewers independently extracted data using a prespecified and standardized data extraction form. All information was verified against the full text of the original studies, including the main text, tables, figure legends, appendices, and supplementary materials, rather than being determined from abstracts alone. Extracted information included the first author, year of publication, country or region, study design, survey programme or data source, sample size, participant age, proportion of women, study setting, built-environment variables and their corresponding domains, built-environment measurement methods, spatial scale, LTPA outcome category, physical activity assessment instrument, specific outcome definition, effect measure, OR and 95% CI, statistical model, and adjusted confounders. Detailed characteristics of the included studies are summarized in Supplementary Table S1.

When a study reported multiple eligible built-environment variables, spatial scales, exposure definitions, activity thresholds, or effect estimates, each was extracted separately. To ensure data traceability, each independent study sample and each effect estimate contributed by that sample were assigned unique identifiers. Multiple effect estimates originating from the same independent study sample were treated as statistically dependent in subsequent analyses.

Original built-environment variables were first classified into the nine prespecified domains according to their conceptual definitions and measurement content. When the definition, measurement direction, or exposure increment of a variable was unclear, the full text, tables, supplementary materials, and, where necessary, the measurement scales or instruments cited in the original article were further examined. LTPA outcomes were consistently classified as LTW, NLTW, or General LTPA according to the operational definitions described above. Detailed operational definitions and classification rules for the nine built-environment domains and the three LTPA outcome categories are provided in Supplementary Method S2. The corresponding operational definitions, measurement rules, and analytical treatment of CAT and UNIT effect scales are summarized in Supplementary Table S2.

Any disagreements between the two reviewers regarding variable classification, outcome classification, effect direction, or data extraction were resolved by re-examining the full text and through discussion. If consensus could not be reached, a third reviewer adjudicated.

#### 2.2.2 Methodological quality assessment

Two reviewers independently assessed the methodological quality of the included studies using the Joanna Briggs Institute (JBI) Critical Appraisal Checklist for Analytical Cross-Sectional Studies (17). The checklist comprises eight appraisal items covering the inclusion criteria for the sample, description of the study participants and setting, validity and reliability of exposure measurement, use of objective and standardized criteria for measurement of the condition, identification and management of confounding factors, validity and reliability of outcome measurement, and appropriateness of the statistical analysis.

Each item was rated in accordance with the JBI tool as “Yes,” “No,” “Unclear,” or “Not applicable.” No numerical scores were assigned to individual items, and no total score or high-, moderate-, or low-quality category was derived from the number of “Yes” responses. Methodological quality was evaluated holistically on the basis of the item-level judgements, and an overall appraisal was assigned using the JBI categories of “Include,” “Exclude,” or “Seek further info.”

When insufficient information was available to make a clear judgement for a specific item, the main text, appendices, supplementary materials, and other relevant study reports were further examined before a final judgement was made. Disagreements between the two reviewers were resolved by re-examining the original report and through discussion; if consensus could not be reached, a third reviewer adjudicated. Item-level judgements for all eight JBI criteria and the overall appraisal for each included study are presented in Supplementary Table S3.

#### 2.2.3 Certainty of evidence assessment

To further assess the certainty of the evidence supporting the study findings, the Grading of Recommendations Assessment, Development and Evaluation (GRADE) framework was applied to provide a structured evaluation of the associations between different leisure-time physical activity outcomes and built-environment domains. The assessment was conducted for each combination of the three LTPA outcome categories—LTW, NLTW, and General LTPA—and the nine built-environment domains: traffic safety (TS), destination accessibility (DA), environmental safety (ES), aesthetics (AE), parks and green spaces (PG), residential density (RD), land-use mix (LUM), street connectivity (SC), and sidewalk quality (SQ).

Evidence was evaluated primarily across five domains: risk of bias, inconsistency, indirectness, imprecision, and publication bias. The overall certainty of evidence for each outcome–domain combination was further informed by the stability of the pooled effect estimates, 95% prediction intervals, CR2-robust inference, and the results of sensitivity analyses. Certainty of evidence was classified into four levels: high, moderate, low, or very low. Detailed assessments are presented in Supplementary Table S11.

### 2.3 Meta-analytic procedures

#### 2.3.1 Effect size calculation and harmonization

Odds ratios (ORs) with 95% confidence intervals (95% CIs) were used as the effect measure. Before formal analysis, the meaning and direction of each built-environment exposure were examined, and effect directions were harmonized so that an OR > 1 indicated an association between a more favourable community built environment and a higher level or greater likelihood of LTPA, whereas an OR < 1 indicated an association in the opposite direction.

For indicators in which higher values represented a poorer or less favourable built environment, such as greater traffic hazards, greater environmental hazards, or longer distances to destinations, effect estimates were inverted after confirming the meaning of the exposure variable:


$${\text{OR}}^{*}=1/\text{OR}$$

The corresponding 95% CI was transformed as follows:


$${\mathrm{CI}}^{*}\text{lower}=1/\text{CIupper},\;\;\;{\mathrm{CI}}^{*}\text{upper}=1/\text{CIlower}$$

No transformation was applied when the original study had already coded the effect estimate in the direction of a more favourable environment.

ORs were subsequently transformed to the natural logarithmic scale for meta-analysis. For effect estimates reported as ORs with 95% CIs, the log OR, standard error, and sampling variance were calculated using standard methods (18):


yi=ln(ORi)

$$\mathrm{SEi}=[\ln(\text{CIupper})-\ln(\text{CIlower})]/3.92,\;\;\;\mathrm{vi}={\mathrm{SEi}}^{2}$$

All models were fitted on the log OR scale, and pooled estimates were exponentiated back to the OR scale for reporting. Detailed transformation procedures are provided in Supplementary Method S3.

Effect estimates were classified as either categorical contrast effects (CAT) or continuous unit effects (UNIT) according to how built-environment exposures were statistically expressed in the original studies. CAT estimates represented categorical comparisons with a clearly defined reference group, whereas UNIT estimates represented continuous effects for which the exposure increment or measurement scale could be clearly identified. Because CAT and UNIT estimates represent different statistical estimands, CAT was treated as the primary analysis and UNIT as the secondary analysis. They were meta-analysed separately and were not directly combined to obtain a common pooled OR. Detailed classification criteria are provided in Supplementary Method S2 and Supplementary Table S2.

#### 2.3.2 Data processing and statistical analysis

Because a single independent study sample could report multiple built-environment factors, spatial scales, or effect estimates and therefore contribute multiple correlated effects to the same meta-analysis, three-level random-effects meta-analysis was used to account for the dependency structure among effect estimates (10, 19). The three-level model partitioned variability into three components: Level 1 represented sampling error, Level 2 represented variability among effect estimates within the same independent study sample, and Level 3 represented variability between independent study samples. Random-effects variance components were estimated using restricted maximum likelihood (REML). Separate three-level models were fitted for CAT and UNIT estimates, and pooled ORs, 95% CIs, and 95% prediction intervals (PIs) were reported. Heterogeneity was assessed using Cochran’s Q test, variance components (τ^2^), and I^2^ statistics, with heterogeneity further partitioned into Level 2 and Level 3 components (11, 20). The 95% PI was used to indicate the range within which the true effect of a new study conducted under similar conditions would be expected to fall (21). In addition, the full three-level model and corresponding reduced models were fitted using maximum likelihood estimation, and likelihood-ratio tests (LRTs), the Akaike information criterion (AIC), and the Bayesian information criterion (BIC) were used to evaluate whether the addition of random-effects levels improved model fit (19).

Because most primary studies did not report the exact sampling covariance among multiple effect estimates derived from the same study, cluster-robust variance estimation (RVE) was applied to the three-level models using the independent study sample as the clustering unit. CR2 small-sample correction and Satterthwaite approximations for the degrees of freedom were used to assess the robustness of the primary statistical inferences to within-study dependence structures (22–24). RVE allows robust inference for correlated effect estimates when the exact within-study covariance structure is unknown, while small-sample corrections help reduce inflation of the type I error rate when the number of independent study clusters is limited.

To explore potential sources of heterogeneity, prespecified subgroup analyses and meta-regression analyses were conducted where sufficient data were available. Categorical moderators included built-environment domain, study region, built-environment measurement method, sample sex composition, spatial scale, and urban/rural or urbanization context. Continuous moderators included mean age, proportion of women, and year of publication. Between-subgroup differences were evaluated using omnibus tests of moderators, and CR2-robust inference was additionally applied to both subgroup analyses and meta-regression models. Given that meta-regression represents an exploratory study-level analysis and may be influenced by the number of studies, the distribution of moderator variables, and residual heterogeneity, no definitive inferences were made for models with insufficient numbers of independent studies or inadequate moderator information (25).

All analyses were conducted in R version 4.5.3. The metafor package was primarily used to fit three-level random-effects models and conduct moderator analyses, the clubSandwich package was used for CR2 cluster-robust inference, and packages including orchaRd were used for visualization of the results (26). All statistical tests were two-sided, with *p* < 0.05 considered statistically significant. Definitions and interpretive guidance for the principal statistical terms used in the three-level meta-analysis are provided in Supplementary Method S4.

#### 2.3.3 Publication bias and small-study effects

Publication bias and small-study effects were initially assessed using conventional funnel plots and contour-enhanced funnel plots. Contour-enhanced funnel plots display regions corresponding to different levels of statistical significance and can therefore assist in distinguishing potential publication bias from other sources of funnel plot asymmetry, such as between-study heterogeneity (27).

Given that a single independent study sample could contribute multiple correlated effect estimates, the primary assessment was based on a three-level Egger-type meta-regression, with the standard error of the effect estimate entered as a moderator. CR2 cluster-robust inference was additionally applied using the independent study sample as the clustering unit. As a sensitivity analysis, sampling variance was used as an alternative moderator, while the conventional Egger’s test was performed as a supplementary diagnostic analysis (28).

In addition, the random-effects trim-and-fill method was used to assess the potential influence of missing effect estimates on the pooled results (29). Because the conventional trim-and-fill method does not explicitly account for the dependency among multiple effect estimates originating from the same study, it was considered an auxiliary diagnostic procedure rather than a basis for primary statistical inference. For models containing a limited number of independent study samples, results from funnel plots and Egger-type tests were interpreted cautiously. Funnel plot asymmetry may arise from publication bias, genuine heterogeneity, differences in study precision, or other study characteristics and was therefore not interpreted as direct evidence of publication bias (30).

#### 2.3.4 Sensitivity analyses

First, potential outlying effect estimates were identified using studentised residuals from the three-level model. Effect estimates with an absolute studentised residual > 1.96 were flagged as potential outliers. After excluding these potential outliers, the three-level REML model and the CR2-robust model were refitted, and changes in the pooled OR, 95% CI, 95% PI, and heterogeneity estimates were examined. Second, a leave-one-study/sample-out analysis was conducted. In each iteration, one independent study sample and all effect estimates contributed by that sample were removed before refitting the model, in order to assess whether the overall findings were disproportionately driven by any single study sample (31).

Finally, a joint CAT–UNIT sensitivity analysis was performed by including both types of effect estimates in the same model and treating effect scale as a moderator. Models assuming a shared heterogeneity structure and scale-specific heterogeneity structures were fitted separately, with CR2-robust inference applied in each case. CAT and UNIT estimates from the joint models were compared with the corresponding estimates from their separate models to evaluate the robustness of the strategy of modelling the two effect scales separately. This analysis was not intended to estimate a common overall OR across the two effect scales.

## 3 Results

### 3.1 Study selection

The study selection process and reasons for exclusion at the full-text stage are presented in Figure 1, with the complete flow diagram also provided in Supplementary Figure S1. The database searches yielded 1,690 records, with an additional 6 records identified through reference-list screening. After duplicate removal, 1,186 records underwent title and abstract screening, of which 1,113 were excluded at this stage. The full texts of 73 articles were subsequently assessed for eligibility, and 34 studies were ultimately included in this systematic review and three-level meta-analysis.

**Figure 1:**
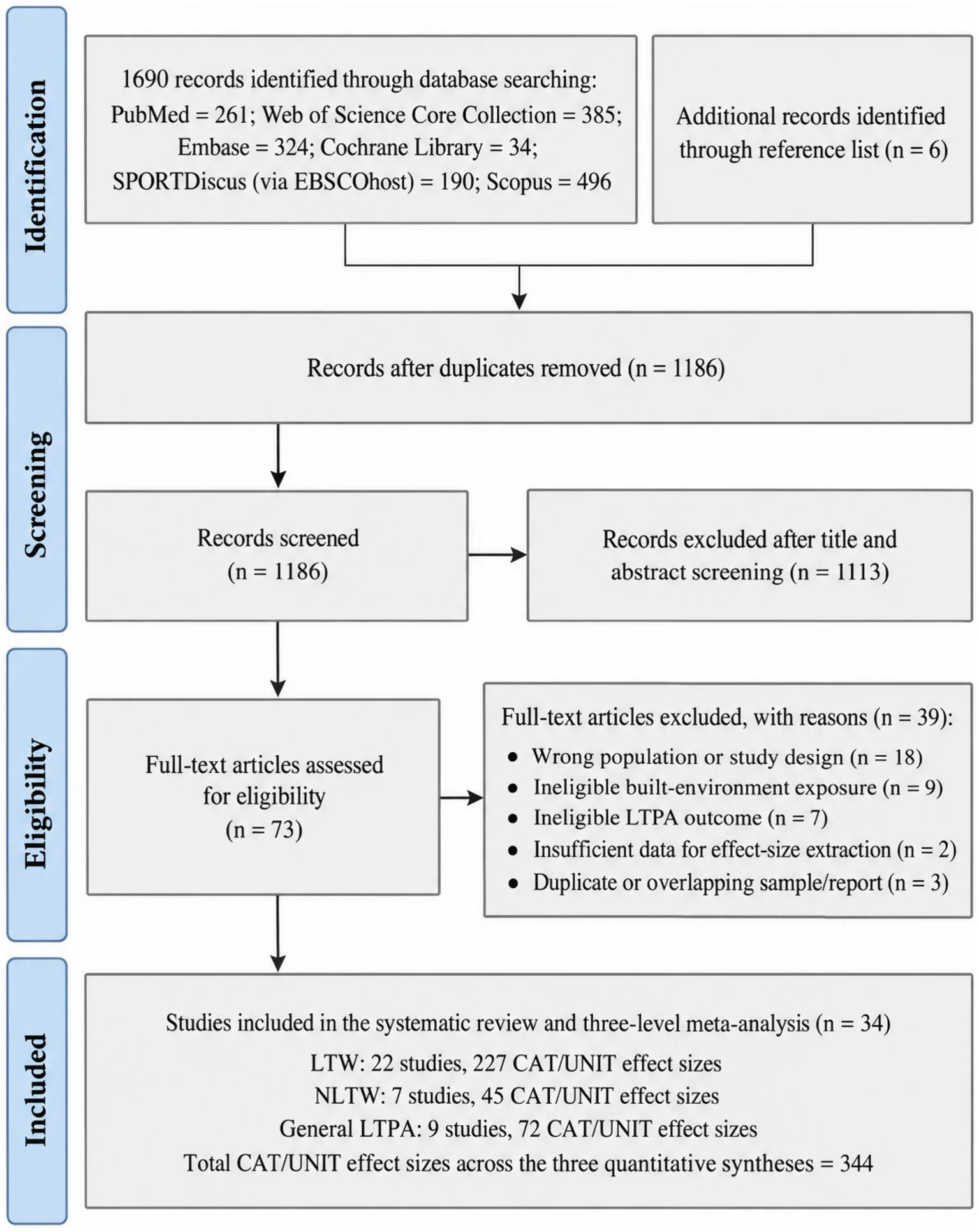
PRISMA flow diagram of study selection. PRISMA, Preferred Reporting Items for Systematic Reviews and Meta-Analyses; LTPA, leisure-time physical activity; LTW, leisure-time walking; NLTW, neighbourhood leisure-time walking; General LTPA, general leisure-time physical activity. SPORTDiscus (via EBSCOhost) indicates that the SPORTDiscus database was searched through the EBSCOhost platform. CAT denotes categorical contrast effect estimates, and UNIT denotes continuous unit effect estimates. Because some studies reported more than one eligible outcome, a single study could contribute effect estimates to more than one outcome category.

By outcome category, 22 studies were included in the leisure-time walking (LTW) analysis, contributing a total of 227 CAT and UNIT effect estimates; 7 studies were included in the neighbourhood leisure-time walking (NLTW) analysis, contributing 45 CAT and UNIT effect estimates; and 9 studies were included in the General LTPA analysis, contributing 72 CAT and UNIT effect estimates. Across the three quantitative syntheses, a total of 344 CAT and UNIT effect estimates were included. Because some studies reported more than one eligible LTPA outcome, individual studies could contribute effect estimates to more than one outcome category.

### 3.2 Characteristics of included studies

A total of 34 studies published between 2000 and 2025 were included in this systematic review and three-level meta-analysis, all of which employed a cross-sectional study design. The included studies were conducted across a range of countries and regions. Nine studies were conducted in the United States (32–40), four each in Hong Kong, China (41–44) and Australia (45–48), three each in Brazil (49–51) and Japan (52–54), and two each in mainland China (55, 56) and Colombia (57, 58). One study each was conducted in South Korea (59), the Netherlands (60), Taiwan, China (61), Canada (62), the United Kingdom (63) and Belgium (64), while one study (65) was conducted across the Czech Republic, Poland, and Slovakia.

Sample sizes ranged from 114 to 73,523 participants. Study populations comprised middle-aged and older adults or older adults, with most studies using an eligibility threshold of 60 or 65 years and above; several studies adopted thresholds of 55 years and above or other age criteria. Nineteen studies were conducted in urban settings, 12 included areas with varying levels of urbanization or mixed settings, one was conducted in a rural setting, one in an older-adult residential facility setting, and one did not clearly report the study setting. Detailed characteristics of the included studies are presented in Supplementary Table S1.

Built-environment exposures covered all nine prespecified domains. Destination accessibility (DA) was the most frequently assessed domain, evaluated in 26 studies. Environmental safety (ES), aesthetics (AE), and sidewalk quality (SQ) were each assessed in 19 studies; residential density (RD), street connectivity (SC), and traffic safety (TS) were each assessed in 17 studies; parks and green spaces (PG) were assessed in 16 studies; and land-use mix (LUM) was assessed in 11 studies. Considerable variation was observed in the measurement of built-environment attributes. Fifteen studies primarily used objective measures, including GIS-based measures, spatial databases, or environmental audits; 13 primarily used perceived measures based on NEWS or other questionnaires; and 6 combined objective and perceived measures. Spatial scales included self-defined neighbourhoods, administrative spatial units, and GIS-based buffers of varying radii. Objective spatial scales ranged from relatively small neighbourhood areas to approximately 1,200 m and 2-mile buffers, and some studies reported estimates across multiple buffer sizes.

LTPA outcomes were classified into LTW, NLTW, and General LTPA according to the prespecified operational definitions. Studies of LTW primarily assessed walking undertaken for leisure, recreation, or exercise, whereas NLTW was further restricted to leisure-time walking explicitly occurring within the residential community or neighbourhood. General LTPA encompassed leisure-time cycling, combined walking and cycling, overall leisure-time physical activity, and other eligible forms of leisure-time physical activity. Physical activity was primarily assessed using questionnaires or standardized instruments, including the International Physical Activity Questionnaire (IPAQ), Community Healthy Activities Model Program for Seniors (CHAMPS), Neighborhood Walking Questionnaire (NWQ), Short Questionnaire to Assess Health-enhancing Physical Activity (SQUASH), Physical Activity Scale for the Elderly (PASE), and Active Australia Survey; a small number of studies used travel diaries. Operational definitions of the outcomes varied across studies and included any participation versus no participation, weekly walking duration, ≥150 min/week, and specific frequency thresholds.

Effect estimates were predominantly reported as adjusted ORs. Some studies reported adjusted prevalence odds ratios or exponentiated regression coefficients that could be expressed on the OR scale. Statistical models generally adjusted for age, sex, education, socioeconomic status, health status, and other study-specific confounders.

### 3.3 Methodological quality assessment

The JBI critical appraisal showed that the 34 included studies received a “Yes” judgement for most appraisal items. All studies were rated “Yes” for Q2 (description of study participants and setting), Q5 (identification of confounding factors), Q6 (strategies for addressing confounding factors), and Q8 (appropriateness of statistical analysis). For Q1, 32 studies (94.1%) were rated “Yes” and 2 (5.9%) were rated “Unclear”; for Q3, 30 studies (88.2%) were rated “Yes” and 4 (11.8%) were rated “Unclear”; for Q4, 32 studies (94.1%) were rated “Yes” and 2 (5.9%) were rated “No”; and for Q7, 26 studies (76.5%) were rated “Yes” and 8 (23.5%) were rated “Unclear.” Accordingly, the main item-level uncertainty was concentrated in the validity and reliability of outcome measurement, followed by exposure measurement. Based on the JBI overall appraisal, all 34 studies were classified as “Include.” The overall distribution of item-level judgements and study-level appraisals is presented in Figure 2, with the complete appraisal figure provided in Supplementary Figure S2 and study-specific results presented in Supplementary Table S3.

**Figure 2:**
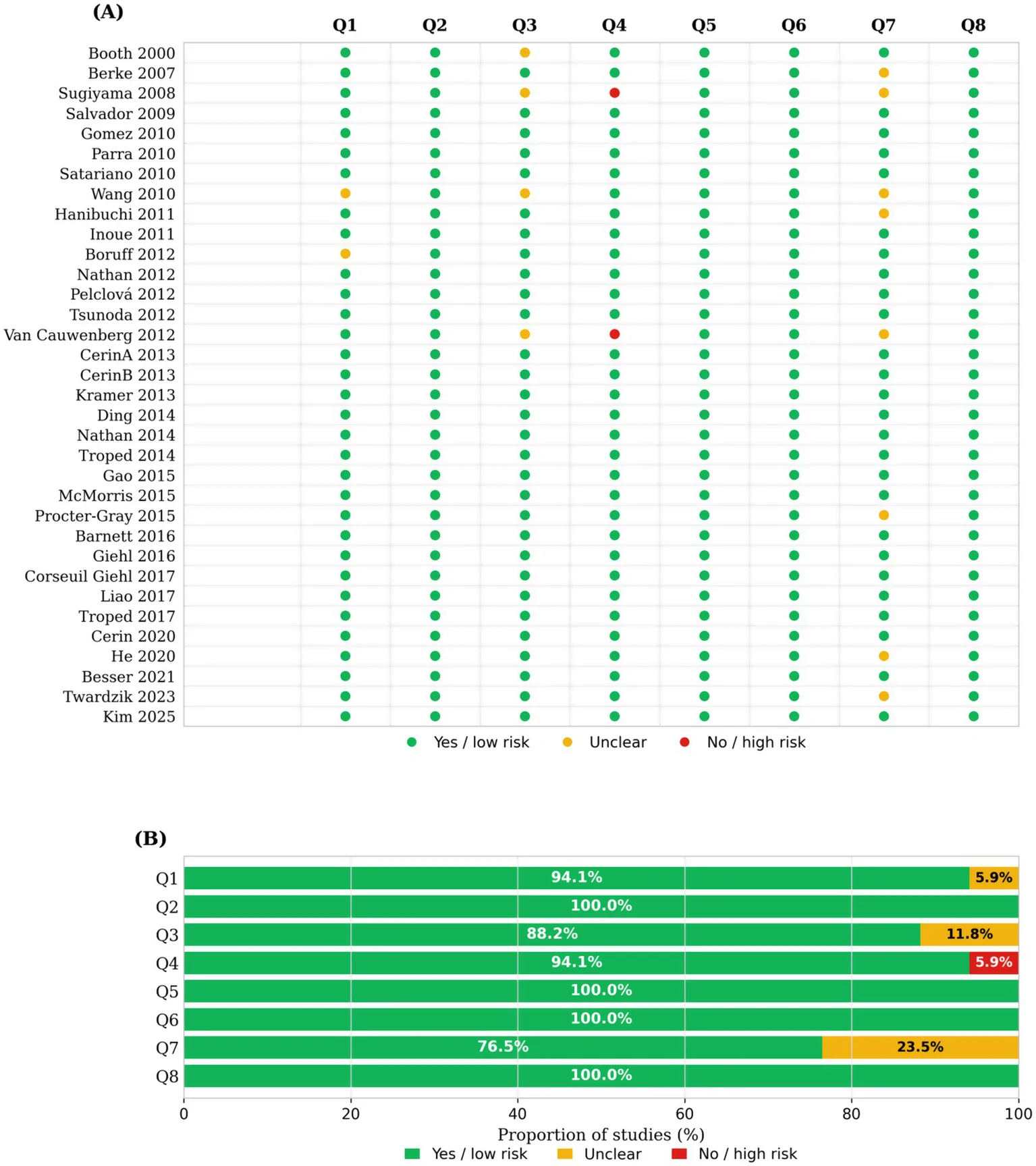
Methodological quality assessment of the included studies. **(A)** Study-level judgements across the eight JBI appraisal items for the 34 included studies; **(B)** proportions of judgements for each appraisal item. Green indicates “Yes,” yellow indicates “Unclear,” and red indicates “No.” The specific criteria for Q1–Q8 and detailed study-level appraisal results are provided in Supplementary Table S3.

### 3.4 Main meta-analysis results

The LTW, NLTW, and General LTPA outcomes were quantitatively synthesised separately using three-level random-effects models, with CAT and UNIT effect estimates analysed according to effect scale. CAT was treated as the primary analysis and UNIT as the secondary analysis. Pooled ORs, 95% CIs, 95% prediction intervals (PIs), τ^2^, I^2^, and results from cluster-robust variance estimation (RVE) with CR2 small-sample correction for each model are summarized in Table 1.

**Table 1:** Overall pooled associations between the community built environment and the three types of leisure-time physical activity.

Outcome/scale	k (g)	Pooled OR (95%CI)	*p*	95%PI	τ^2^ L3 / L2	I^2^ total (L3 / L2)	CR2-RVE OR (95%CI);*p*
LTW CAT	138 (14)	1.103 (1.022–1.191)	0.012	0.788–1.545	0.014/0.0130	55.9 (28.7/27.1)	1.103 (1.015–1.200); 0.025
LTW UNIT	89 (11)	1.068 (0.998–1.144)	0.058	0.856–1.333	0.011/0.0002	98.9 (97.0/1.9)	1.068 (0.989–1.154); 0.084
NLTW CAT	12 (3)	2.008 (0.879–4.591)	0.090	0.352–11.448	0.358/0.1260	90.0 (66.6/23.4)	2.008 (0.385–10.464); 0.210
NLTW UNIT	33 (5)	1.063 (1.017–1.112)	0.008	0.932–1.213	0.001/0.0030	92.8 (18.1/74.8)	1.063 (0.978–1.156); 0.094
General LTPA CAT	49 (7)	1.180 (1.089–1.278)	<0.001	0.911–1.528	0.006/0.0090	57.4 (24.0/33.3)	1.180 (1.067–1.304); 0.007
General LTPA UNIT	23 (4)	1.154 (0.871–1.528)	0.302	0.623–2.136	0.070/0.0000	100.0 (100.0/0.0)	1.154 (0.756–1.761); 0.358

k denotes the number of effect estimates, and g denotes the number of independent study samples; PI, prediction interval. τ^2^ L3 and τ^2^ L2 represent the between-study-sample and within-study-sample variance components, respectively. Values in parentheses for I^2^ correspond to Level 3 and Level 2 I^2^, respectively. Variance components (τ^2^) are reported to three or four decimal places, with additional precision retained for small non-zero estimates to avoid obscuring them through rounding. RVE was clustered at the independent study-sample level and implemented with CR2 small-sample correction. Full results for Cochran’s Q tests, CR2 degrees of freedom, and model-structure comparisons are provided in Supplementary Table S4.

The orchard plots for the three primary CAT analyses are presented in Figure 3. Complete overall CAT and UNIT analysis plots for LTW, NLTW, and General LTPA are provided in Supplementary Figures S3, S9, S15, respectively. Full results for Cochran’s Q tests, CR2 degrees of freedom, and model-structure comparisons based on likelihood-ratio tests (LRTs), Akaike information criterion (AIC), and Bayesian information criterion (BIC) are presented in Supplementary Table S4.

**Figure 3:**
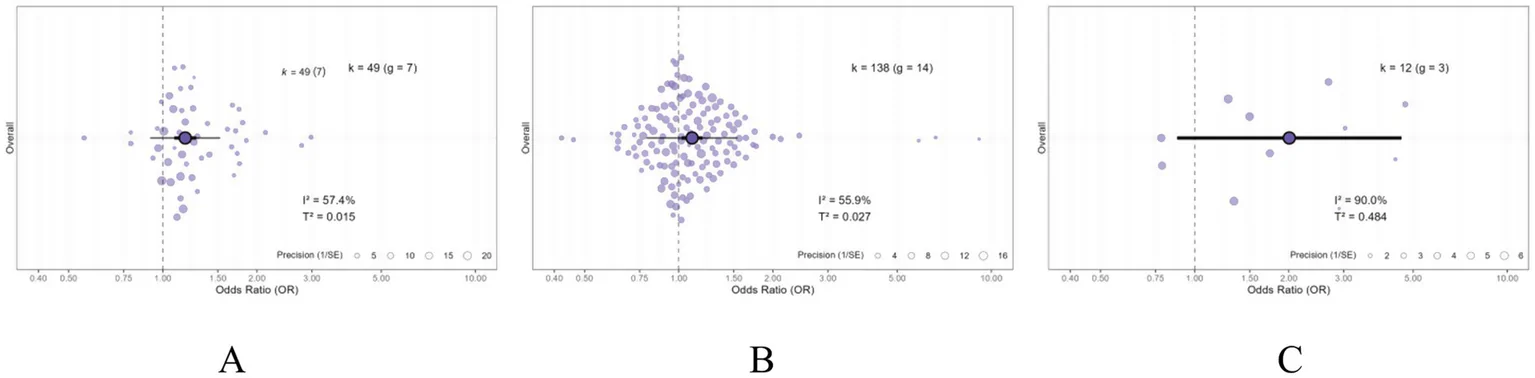
Orchard plots for the primary CAT analyses of associations between the community built environment and the three types of leisure-time physical activity. **(A)** LTW; **(B)** NLTW; **(C)** General LTPA. Light-coloured points represent individual effect estimates, and larger solid points represent pooled ORs from the three-level models. Black intervals indicate the 95% CIs and 95% prediction intervals. An OR > 1 indicates an association between a more favourable community built environment and a higher likelihood of LTPA. k denotes the number of effect estimates, and g denotes the number of independent study samples.

#### 3.4.1 Leisure-time walking (LTW)

The primary CAT analysis for LTW included 14 independent study samples (g = 14) and 138 effect estimates (k = 138). The three-level random-effects model showed a significant positive association between a more favourable community built environment and a higher likelihood of LTW (OR = 1.103, 95% CI 1.022–1.191, *p* = 0.012). The 95% PI was 0.788–1.545 and crossed OR = 1. Cochran’s Q test indicated statistically significant heterogeneity (Q(137) = 300.46, *p* < 0.001). The variance components were τ^2^ (Level 3) = 0.014 and τ^2^ (Level 2) = 0.0130, with a total I^2^ of 55.9%; Level 3 I^2^ was 28.7% and Level 2 I^2^ was 27.1%, indicating that heterogeneity was distributed across both the between-study-sample and within-study-sample levels. After applying RVE with CR2 small-sample correction, the positive association remained statistically significant (OR = 1.103, 95% CI 1.015–1.200, *p* = 0.025), consistent with the primary model.

The secondary UNIT analysis for LTW included 11 independent study samples (g = 11) and 89 effect estimates (k = 89). The pooled effect from the three-level random-effects model was OR = 1.068 (95% CI 0.998–1.144, *p* = 0.058), which did not reach statistical significance. The 95% PI was 0.856–1.333 and crossed OR = 1. Cochran’s Q test indicated statistically significant heterogeneity (Q(88) = 316.60, *p* < 0.001). The variance components were τ^2^ (Level 3) = 0.011 and τ^2^ (Level 2) = 0.0002, with a total I^2^ of 98.9%; Level 3 I^2^ was 97.0% and Level 2 I^2^ was 1.9%, indicating that heterogeneity was predominantly concentrated at the between-study-sample level. The CR2-RVE result was also not statistically significant (OR = 1.068, 95% CI 0.989–1.154, *p* = 0.084), consistent with the statistical inference from the primary model.

#### 3.4.2 Neighbourhood leisure-time walking (NLTW)

The primary CAT analysis for NLTW included 3 independent study samples (g = 3) and 12 effect estimates (k = 12). The pooled effect from the three-level random-effects model was OR = 2.008 (95% CI 0.879–4.591, *p* = 0.090), which did not reach statistical significance. The 95% PI was 0.352–11.448 and crossed OR = 1. Cochran’s Q test indicated statistically significant heterogeneity (Q(11) = 56.46, *p* < 0.001). The variance components were τ^2^ (Level 3) = 0.358 and τ^2^ (Level 2) = 0.1260, with a total I^2^ of 90.0%; Level 3 I^2^ was 66.6% and Level 2 I^2^ was 23.4%, indicating that heterogeneity was predominantly concentrated at the between-study-sample level. The CR2-RVE result also did not reach statistical significance (OR = 2.008, 95% CI 0.385–10.464, *p* = 0.210), consistent with the statistical inference from the primary model.

The secondary UNIT analysis for NLTW included 5 independent study samples (g = 5) and 33 effect estimates (k = 33). The three-level random-effects model showed a statistically significant positive association (OR = 1.063, 95% CI 1.017–1.112, *p* = 0.008). The 95% PI was 0.932–1.213 and crossed OR = 1. Cochran’s Q test indicated statistically significant heterogeneity (Q(32) = 76.21, *p* < 0.001). The variance components were τ^2^ (Level 3) = 0.001 and τ^2^ (Level 2) = 0.0030, with a total I^2^ of 92.8%; Level 3 I^2^ was 18.1% and Level 2 I^2^ was 74.8%, indicating that heterogeneity was predominantly concentrated among different effect estimates within the same study sample. After CR2-RVE correction, however, the association was no longer statistically significant (OR = 1.063, 95% CI 0.978–1.156, *p* = 0.094), indicating that the statistical inference for this result was sensitive to robust variance correction.

#### 3.4.3 General leisure-time physical activity (general LTPA)

The primary CAT analysis for General LTPA included 7 independent study samples (g = 7) and 49 effect estimates (k = 49). The three-level random-effects model showed a significant positive association between a more favourable community built environment and a higher likelihood of General LTPA (OR = 1.180, 95% CI 1.089–1.278, *p* < 0.001). The 95% PI was 0.911–1.528 and crossed OR = 1. Cochran’s Q test indicated statistically significant heterogeneity (Q(48) = 116.20, *p* < 0.001). The variance components were τ^2^ (Level 3) = 0.006 and τ^2^ (Level 2) = 0.0090, with a total I^2^ of 57.4%; Level 3 I^2^ was 24.0% and Level 2 I^2^ was 33.3%, indicating that heterogeneity was distributed across both the between-study-sample and within-study-sample levels. The CR2-RVE result remained statistically significant (OR = 1.180, 95% CI 1.067–1.304, *p* = 0.007), consistent with the primary model.

The secondary UNIT analysis for General LTPA included 4 independent study samples (g = 4) and 23 effect estimates (k = 23). The pooled effect from the three-level random-effects model was OR = 1.154 (95% CI 0.871–1.528, *p* = 0.302), which did not reach statistical significance. The 95% PI was 0.623–2.136 and crossed OR = 1. Cochran’s Q test indicated statistically significant heterogeneity (Q(22) = 124.53, *p* < 0.001). The variance components were τ^2^ (Level 3) = 0.070 and τ^2^ (Level 2) = 0.0000, with a total I^2^ of 100.0%; Level 3 I^2^ was 100.0% and Level 2 I^2^ was 0.0%, indicating that the estimated heterogeneity was concentrated entirely at the between-study-sample level. The CR2-RVE result was likewise not statistically significant (OR = 1.154, 95% CI 0.756–1.761, *p* = 0.358), consistent with the statistical inference from the primary model.

Overall, the primary CAT analyses for LTW and General LTPA were statistically significant in both the three-level models and the CR2-RVE analyses, whereas the primary CAT analysis for NLTW was not statistically significant. Among the secondary UNIT analyses, only NLTW reached statistical significance in the conventional three-level model, but this association was no longer statistically significant after CR2-RVE correction.

### 3.5 Moderator analyses

#### 3.5.1 Subgroup analyses

To explore potential sources of heterogeneity, subgroup analyses were conducted, where sufficient data were available, according to built-environment domain, geographic region, built-environment measurement method, sample sex composition, spatial scale, and urbanization context. Because some subgroups were supported by only one or two independent study samples, and because some CR2 omnibus tests could not be estimated reliably owing to insufficient effective degrees of freedom, subgroup findings were interpreted primarily as exploratory evidence. The main CAT subgroup results by built-environment domain are presented in Figure 4. Complete CAT and UNIT subgroup analyses for LTW, NLTW, and General LTPA are provided in Supplementary Figures S4, S10, and S16, respectively, with full estimates and CR2 results reported in Supplementary Table S5.

**Figure 4:**
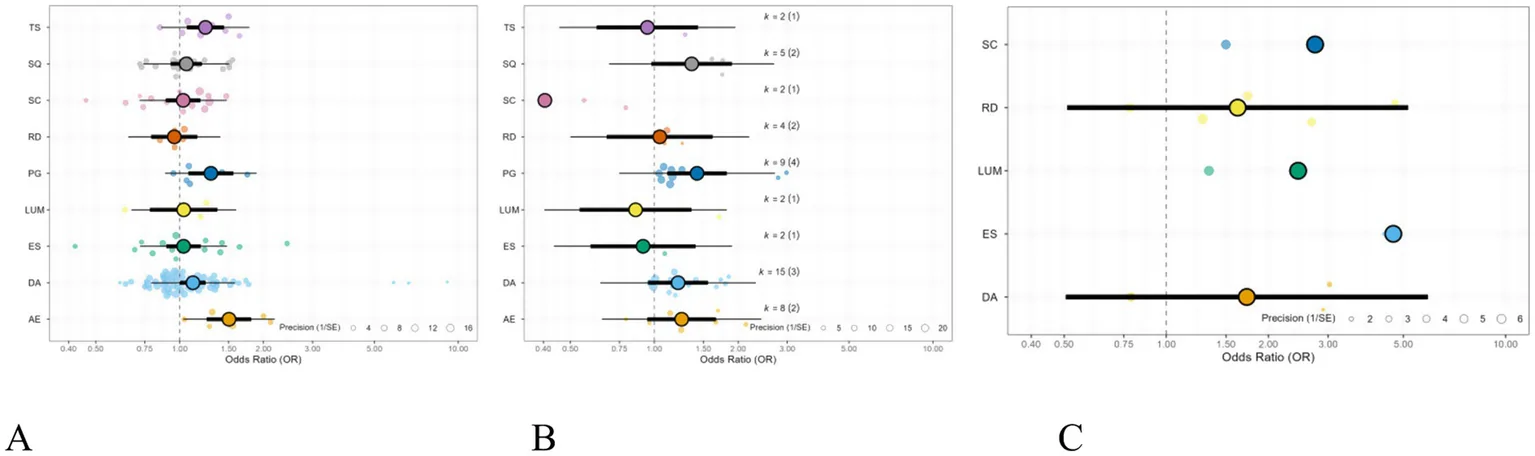
Subgroup orchard plots stratified by built-environment domain in the primary CAT analyses**. **(A)** LTW; **(B)** General LTPA; **(C)** NLTW. Large circles represent the pooled ORs for each built-environment domain, thick black lines indicate 95% CIs, thin black lines indicate 95% prediction intervals, and light-coloured points represent individual effect estimates. The dashed line indicates OR = 1. TS, traffic safety; DA, destination accessibility; ES, environmental safety; AE, aesthetics; PG, parks and green spaces; RD, residential density; LUM, land-use mix; SC, street connectivity; SQ, sidewalk quality. Estimates for NLTW and for some built-environment domains were based on a limited number of independent study samples and should therefore be interpreted with caution.

In the primary CAT analysis for LTW, the conventional three-level model suggested differences across built-environment domains, although the CR2 omnibus test could not be estimated reliably because of insufficient effective degrees of freedom. Domain-specific analyses showed that aesthetics remained significantly associated with a higher likelihood of LTW after CR2 correction (OR = 1.50, 95% CI 1.01–2.23, *p* = 0.046). No stable between-group differences were observed for measurement method, geographic region, sample sex composition, spatial scale, or urbanization context. Although perceived built-environment measures and mixed-urbanization settings yielded statistically significant estimates within their respective strata, the corresponding omnibus between-group tests were not significant; these findings were therefore not interpreted as clear moderator effects. In the secondary UNIT analysis for LTW, the conventional omnibus test for built-environment domain was significant, but the corresponding CR2 omnibus test was similarly unstable. Domain-specific CR2 results showed that destination accessibility was positively associated with LTW (OR = 1.10, 95% CI 1.01–1.20, *p* = 0.031), whereas environmental safety was negatively associated with LTW (OR = 0.83, 95% CI 0.74–0.94, *p* = 0.020). Built-environment measurement method showed only a borderline between-group difference after CR2 correction, while no stable evidence was observed for the remaining moderators.

No reliable subgroup differences were identified in either the CAT or UNIT analyses for NLTW. In the CAT analysis, built-environment domain, measurement method, geographic region, spatial scale, and urbanization context showed no clear moderating effects. Although the conventional UNIT model suggested differences across built-environment domains, each domain was supported by only one or two independent study samples, and no robust evidence was obtained. Other moderators likewise showed no consistent between-group differences, and formal comparisons were not conducted for some variables because of insufficient data. Accordingly, subgroup findings for NLTW were interpreted as exploratory.

In the primary CAT analysis for General LTPA, built-environment measurement method emerged as a comparatively more consistent moderator. Both the conventional model and the CR2 omnibus test indicated between-group differences (CR2 *p* = 0.021). The pooled effect for perceived built-environment measures was OR = 1.28 (95% CI 1.09–1.50, *p* = 0.026), compared with OR = 1.09 (95% CI 0.99–1.21, *p* = 0.060) for objectively measured built-environment exposures. In addition, parks and green spaces were positively associated with General LTPA after CR2 correction (OR = 1.42, 95% CI 1.04–1.96, *p* = 0.035). Street connectivity was supported by only two effect estimates from a single independent study sample and was therefore presented descriptively only. Geographic region and spatial scale showed no moderating effects. Although the CR2 omnibus test for urbanization context reached nominal statistical significance, the effective degrees of freedom were only approximately 1.24; this finding was therefore not considered robust evidence. In the secondary UNIT analysis for General LTPA, several conventional models suggested potential differences by built-environment domain, sample sex composition, or spatial scale; however, the corresponding CR2 tests were constrained by sparse clustering and did not reveal a reproducible pattern of moderation.

#### 3.5.2 Meta-regression

Meta-regression analyses of continuous moderators further examined whether mean age, proportion of women, and year of publication could explain variation in effect estimates. Overall, none of the three continuous moderators showed a consistent moderating effect across outcomes and effect scales in both the conventional models and CR2-robust analyses. Full regression coefficients, 95% CIs, and CR2 results are presented in Supplementary Table S6, with corresponding plots shown in Figure 5 and Supplementary Figures S5, S11, S17.

**Figure 5:**
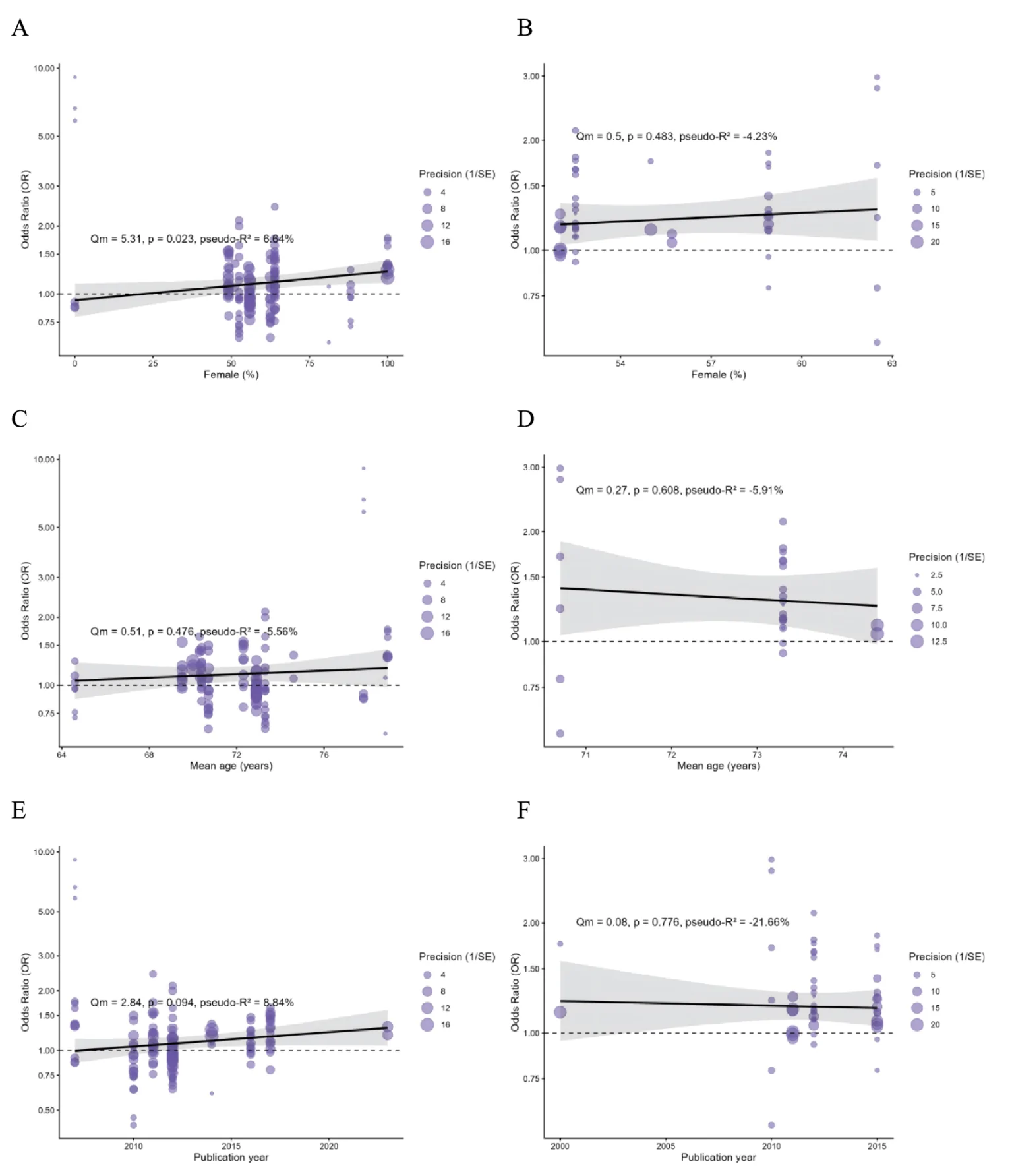
Meta-regression plots for the primary CAT analyses. **(A)** LTW and proportion of women; **(B)** general LTPA and proportion of women; **(C)** LTW and mean age; **(D)** general LTPA and mean age; **(E)** LTW and year of publication; **(F)** general LTPA and year of publication. Solid lines represent the fitted meta-regression relationships, grey shaded areas indicate 95% CIs, point sizes reflect precision (1/SE), and dashed lines indicate OR = 1. Because the primary CAT analysis for NLTW included only 3 independent study samples, the corresponding meta-regression results are presented as exploratory analyses in Supplementary Figure S11.

In the primary CAT analysis for LTW, the proportion of women showed a positive moderating effect in the conventional model (β = 0.003, 95% CI 0.000–0.005, *p* = 0.023), but this association was no longer statistically significant after CR2 correction (β = 0.0029, 95% CI − 0.0037 to 0.0096, *p* = 0.155). Neither mean age nor year of publication showed evidence of moderation in either the conventional or CR2 models. In the secondary UNIT analysis for LTW, mean age showed a negative moderating effect in the conventional model (β = −0.038, 95% CI − 0.065 to −0.011, *p* = 0.007), but this association also became non-significant after CR2 correction. The proportion of women and year of publication likewise showed no stable associations.

In the primary CAT analysis for NLTW, the proportion of women, mean age, and year of publication did not show robust moderating effects. In the secondary UNIT analysis, the proportion of women yielded a nominally significant result in the CR2 model (β = 0.0461, 95% CI 0.0412–0.0510, *p* = 0.005). However, this analysis included only 2 independent study samples and 3 effect estimates, with a Satterthwaite degree of freedom of 1.00; the finding was therefore not considered reliable evidence of moderation. Mean age and year of publication were not statistically significant.

In the primary CAT analysis for General LTPA, the proportion of women, mean age, and year of publication showed no evidence of moderation. In the secondary UNIT analysis, the proportion of women reached nominal statistical significance in the CR2 model (β = 0.0037, 95% CI 0.0021–0.0053, *p* = 0.022), but the analysis was supported by only 3 independent study samples and had an effective degree of freedom of approximately 1.00; this finding was therefore not interpreted substantively. Year of publication showed no moderating effect. Because of insufficient data, a formal meta-regression of mean age was not conducted for the secondary UNIT analysis of General LTPA.

### 3.6 Publication bias and small-study effects

Publication bias and small-study effects were evaluated comprehensively using contour-enhanced funnel plots, three-level Egger-type meta-regression, CR2-robust Egger tests, and the trim-and-fill method. Because the NLTW analyses and several UNIT models included only a small number of independent study samples, the statistical power of funnel plots and Egger-type tests was limited; therefore, funnel plot asymmetry was not interpreted directly as evidence of publication bias. Complete CAT and UNIT diagnostic plots for LTW, NLTW, and General LTPA are provided in Supplementary Figures S6, S12, S18, respectively, and full results are reported in Supplementary Table S7.

The primary CAT analysis for LTW showed no clear evidence of small-study effects. Neither the three-level SE-Egger test nor its CR2-corrected counterpart was statistically significant (*p* = 0.896 and *p* = 0.956, respectively), and the sensitivity analysis using sampling variance as the moderator was also non-significant (*p* = 0.247). The trim-and-fill method did not estimate any potentially missing effect estimates. Similarly, the secondary UNIT analysis for LTW showed no consistent evidence of small-study effects, with neither the SE-Egger nor CR2 test reaching statistical significance. Although trim-and-fill estimated 6 potentially missing effect estimates, the direction of the adjusted effect remained unchanged.

The NLTW findings require greater caution. In the primary CAT analysis for NLTW, the conventional three-level SE-Egger test and the sensitivity analysis using sampling variance suggested funnel plot asymmetry, but the association was no longer statistically significant after CR2 correction (*p* = 0.107). Moreover, this analysis included only 3 independent study samples. Trim-and-fill also suggested potentially missing effect estimates; therefore, these findings should be interpreted cautiously. Signals of small-study effects were more apparent in the secondary UNIT analysis. Both the three-level SE-Egger test (*p* < 0.001) and the sampling-variance sensitivity test (*p* = 0.002) were statistically significant, whereas the CR2-corrected result was only borderline significant (*p* = 0.068). Trim-and-fill estimated 13 potentially missing effect estimates, and the adjusted two-level pooled effect was reduced to OR = 1.01 (95% CI 0.97–1.06, *p* = 0.565).

Among the three outcomes, the primary CAT analysis for General LTPA showed the most consistent signal of small-study effects. The three-level SE-Egger test was statistically significant (*p* < 0.001), remained significant after CR2 correction (*p* = 0.006), and was also significant in the sensitivity analysis using sampling variance (*p* < 0.001). Trim-and-fill estimated 13 potentially missing effect estimates, and the adjusted two-level pooled effect was attenuated to OR = 1.10 (95% CI 1.01–1.20, *p* = 0.030), although the direction of association remained positive. The secondary UNIT analysis for General LTPA also showed evidence of funnel plot asymmetry. Both the conventional SE-Egger test and the sampling-variance test were statistically significant, whereas the CR2-corrected result did not reach the conventional threshold for statistical significance (*p* = 0.055). Trim-and-fill likewise suggested the possibility of missing effect estimates.

Overall, evidence of small-study effects was inconsistent across outcomes and effect scales. The strongest evidence was observed in the primary CAT analysis for General LTPA, while some evidence was also present in the secondary UNIT analysis for NLTW. In contrast, the primary CAT analysis for LTW showed no clear evidence of small-study effects. Because the trim-and-fill method does not account for dependence among multiple effect estimates within the same study, its results were treated only as supplementary two-level sensitivity diagnostics and did not replace statistical inference from the primary three-level models.

### 3.7 Sensitivity analyses

The robustness of the main findings was evaluated using outlier exclusion, leave-one-study/sample-out analyses, and joint CAT–UNIT models. Complete CAT and UNIT sensitivity analyses for LTW, NLTW, and General LTPA are provided in Supplementary Figures S7, S13, S19, respectively. Results of outlier exclusion and leave-one-study/sample-out analyses are reported in Supplementary Tables S8, S9. Joint CAT–UNIT models are presented in Supplementary Figures S8, S14, S20, with full results provided in Supplementary Table S10.

The primary CAT result for LTW remained statistically significant after excluding 9 potential outlying effect estimates (CR2 OR = 1.12, 95% CI 1.04–1.20, *p* = 0.006), while total I^2^ decreased from 55.9 to 44.6%. In the leave-one-study/sample-out analysis, pooled ORs remained between 1.09 and 1.13 and were statistically significant in every iteration, indicating that the overall result was not driven by any single study. The primary CAT analysis for General LTPA also showed good robustness. After excluding 4 potential outlying effect estimates, the CR2 result remained statistically significant (OR = 1.15, 95% CI 1.05–1.25, *p* = 0.009), while total I^2^ decreased from 57.4 to 41.1%. All leave-one-study/sample-out models likewise remained statistically significant.

In contrast, the NLTW analyses and most secondary UNIT analyses showed lower stability. No potential outliers were identified in the primary CAT analysis for NLTW; however, because only 3 independent study samples were included, effect estimates varied substantially across leave-one-study/sample-out iterations. In the UNIT analysis for NLTW, the CR2 result remained non-significant after outlier exclusion, and the statistical significance of the overall effect changed after removal of individual studies. The UNIT analyses for LTW and General LTPA reached statistical significance in some sensitivity models, but these findings were sensitive to the exclusion of outlying effect estimates or individual studies. Accordingly, these analyses did not alter the overall interpretation that evidence from UNIT analyses was less robust than that from the primary CAT analyses.

The joint CAT–UNIT sensitivity analyses generally reproduced the main findings from the separate models. Taken together, the outlier exclusion, leave-one-study/sample-out, and joint-model analyses supported the robustness of the primary CAT findings for LTW and General LTPA, whereas greater uncertainty remained for NLTW and the UNIT analyses. The joint models further supported treating CAT and UNIT as distinct statistical estimands and modelling them separately rather than combining them into a single overall effect (Figure 6).

**Figure 6:**
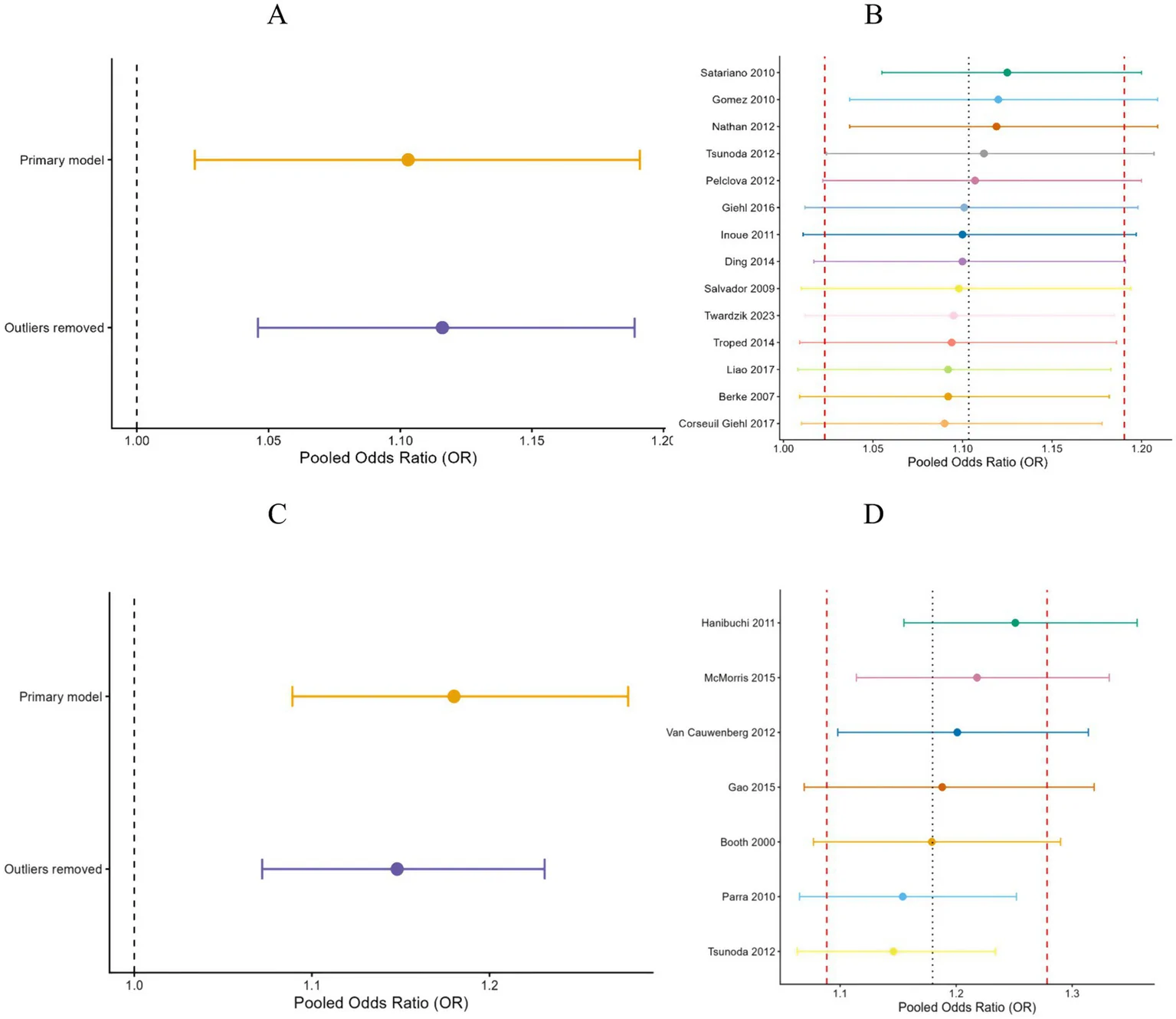
Sensitivity analyses for the primary CAT analyses. **(A)** Comparison of LTW estimates before and after outlier exclusion; **(B)** leave-one-study/sample-out analysis for LTW; **(C)** comparison of General LTPA estimates before and after outlier exclusion; **(D)** leave-one-study/sample-out analysis for General LTPA. The dashed line indicates OR = 1. In the leave-one-study/sample-out analyses, the three-level model was refitted after sequentially removing one independent study sample and all effect estimates contributed by that sample. Because the primary CAT analysis for NLTW included only a small number of independent study samples, its sensitivity analysis is presented in Supplementary Figure S13.

### 3.8 Certainty of evidence

Based on the adapted GRADE framework, the certainty of evidence across outcome–built-environment domain combinations was generally rated as low to moderate (Supplementary Table S11). For LTW, the certainty of evidence was rated as moderate for traffic safety (TS), destination accessibility (DA), aesthetics (AE), parks and green spaces (PG), and residential density (RD), whereas the remaining built-environment domains were rated as low. For NLTW, the certainty of evidence was rated as moderate for traffic safety (TS) and destination accessibility (DA), while all other domains were rated as low. For General LTPA, the certainty of evidence was rated as moderate for traffic safety (TS), destination accessibility (DA), aesthetics (AE), parks and green spaces (PG), and residential density (RD), whereas the remaining domains were rated as low. Overall, outcome–domain combinations supported by a relatively larger evidence base and showing greater stability across robustness analyses were assigned moderate-certainty ratings, whereas combinations based on fewer studies, greater heterogeneity, or less precise effect estimates remained low-certainty evidence.

## 4 Discussion

This systematic review and three-level meta-analysis included 34 cross-sectional studies and systematically evaluated the associations between the community built environment and different types of leisure-time physical activity (LTPA) among older adults. Overall, more favourable community built environments were positively associated with leisure-time walking (LTW) and General LTPA, and the primary CAT findings for both outcomes remained statistically consistent after CR2-robust inference, outlier exclusion, and leave-one-study/sample-out analyses. In contrast, although the overall direction of association for NLTW tended to be positive, the primary CAT analysis did not reach statistical significance, while the UNIT analysis was significant in the conventional model but became non-significant after CR2 correction, indicating that the statistical evidence for this outcome remains unstable. At the same time, the 95% prediction intervals for all three primary CAT models crossed OR = 1, suggesting substantial variation in the average associations across different study contexts.

More importantly, the built-environment association patterns were not fully consistent across different types of LTPA. Aesthetics, destination accessibility, and parks and green spaces showed clearer associations with different activity types or effect scales, broadly consistent with previous reviews of the built environment and physical activity among older adults, which have likewise suggested that different environmental attributes may be associated with different physical activity outcomes. These findings indicate that the relationship between the community built environment and LTPA among older adults may be behaviour- and context-specific. In other words, the association of a particular environmental attribute may depend on the type of activity and the setting in which that activity occurs, rather than all favourable environmental characteristics being associated with all forms of LTPA in the same manner.

### 4.1 Association patterns across LTPA types and potential mechanisms

For LTW, the primary CAT analysis showed that a more favourable community built environment was positively associated with higher levels of leisure-time walking, with aesthetics showing a relatively clear domain-specific association. Previous quantitative and qualitative reviews have similarly reported that environmental aesthetics, greenery, favourable landscape experiences, and pleasant walking environments are closely related to walking and physical activity among older adults. Leisure-time walking differs from commuting or other utilitarian walking in that it is generally more discretionary and experiential. Therefore, in addition to basic conditions that facilitate movement, whether the environment is comfortable, attractive, and conducive to lingering may also influence older adults’ behavioural choices. In a systematic review of qualitative studies among older adults, Moran et al. identified aesthetics, amenities, safety, and walking infrastructure as important physical-environment themes associated with physical activity, suggesting that high-quality greenery, landscape features, and environmental maintenance may increase willingness to engage in leisure-time walking by improving the walking experience and enhancing the attractiveness of outdoor activity (66).

In the UNIT analysis for LTW, destination accessibility was positively associated with higher levels of leisure-time walking, and the underlying mechanism may differ from that of aesthetics. Whereas aesthetics primarily reflects the attractiveness of the activity environment, destination accessibility may increase opportunities for older adults to go outdoors and participate in activity by providing reachable recreational facilities, public spaces, and other activity destinations. Previous meta-analyses and qualitative reviews have likewise identified access to destinations, services, and recreational facilities as important environmental correlates of walking and physical activity among older adults. LTW may therefore be influenced by both environmental experience and the structure of activity opportunities, although different environmental attributes may operate through distinct pathways. Notably, environmental safety showed an inverse association in the UNIT analysis for LTW. Because this finding was based on a limited number of independent study samples, and because the conceptual definitions, measurement approaches, and continuous exposure increments for environmental safety differed across studies, the available evidence is insufficient to support a clear mechanistic interpretation. Methodological reviews have highlighted substantial variation in the definition of environmental indicators, objective versus perceived measurement, and spatial operationalization in studies of neighbourhood environments among older adults, all of which may reduce the comparability of effect estimates (6). This result should therefore be regarded as exploratory and should not be interpreted as evidence that greater environmental safety reduces leisure-time walking among older adults.

Compared with LTW, NLTW has a more direct conceptual correspondence with the built environment surrounding the residence because the activity is explicitly restricted to the community or neighbourhood. Nevertheless, the present study did not identify a stable overall association. One possible explanation is that substantially fewer independent studies were available for NLTW than for LTW, limiting the precision of effect estimates and the reliability of robust inference. In addition, the operational definition of “neighbourhood” varied across studies and included administrative areas, fixed residential buffers, self-defined neighbourhood areas, and participant-perceived neighbourhood boundaries. Spatial exposure research has further shown that fixed buffers centred on residential locations may not fully capture the built environments that individuals actually encounter in daily life, whereas activity-space approaches based on actual mobility trajectories characterise environmental exposure from a different perspective (67). Accordingly, the instability of the NLTW findings should not be interpreted simply as evidence of no association between the community built environment and neighbourhood leisure-time walking; it may also partly reflect mismatch between the defined environmental exposure area and the actual spatial context in which activity occurs.

The primary CAT analysis for General LTPA also showed an overall positive association, with parks and green spaces demonstrating a relatively clear domain-specific association. Previous meta-analyses have reported positive relationships of parks, public open spaces, and recreational facilities with overall physical activity and LTPA among older adults. Compared with leisure-time walking alone, General LTPA encompasses cycling, combined walking and cycling, and other forms of leisure-time physical activity, which may depend more strongly on public spaces capable of accommodating a wider range of activities. Parks and green spaces can provide not only walking routes but also opportunities for cycling, exercise, recreation, and social interaction, and therefore may function as multifunctional spatial resources that support diverse forms of activity. Qualitative evidence has similarly identified green spaces, open spaces, and accessible recreational facilities as important environmental conditions for physical activity among older adults (66).

Taken together, the present findings support a differentiated environment–behaviour association pattern. LTW may be more responsive to environmental experience and destination opportunities, General LTPA may depend more strongly on spatial resources capable of accommodating diverse activities, and NLTW may require a closer spatial match between residential environmental exposure and the actual setting in which activity takes place. These findings also suggest that aesthetics, destination accessibility, and parks and green spaces are best viewed at present as candidate environmental factors warranting further investigation within specific behavioural contexts, rather than as independent determinants exerting uniform effects across all forms of LTPA among older adults.

### 4.2 Heterogeneity, moderators, and robustness of the evidence

Most models in the present study exhibited varying degrees of heterogeneity, and the 95% prediction intervals for all three primary CAT models crossed OR = 1. This indicates that the pooled estimates represent average associations across heterogeneous study conditions and should not be generalized unconditionally to all communities or older populations. Heterogeneity may arise simultaneously from differences in built-environment exposures, definitions of LTPA outcomes, and the spatial correspondence between environmental exposure and behaviour. Previous methodological reviews have shown that variation in neighbourhood definitions, objective versus perceived environmental measures, buffer sizes, and the operationalization of specific indicators can all influence estimates of associations between the built environment and physical activity.

Among the prespecified subgroup analyses, built-environment measurement method emerged as the clearest moderator for General LTPA, with stronger associations observed for perceived environmental measures than for objectively measured environments. Previous studies have likewise shown that perceived and objective environmental measures do not necessarily exhibit identical patterns of association with physical activity. Objective measures primarily capture actual physical conditions, such as street networks, facilities, land use, and spatial structure, whereas perceived measures also incorporate individuals’ subjective evaluations of safety, convenience, comfort, and environmental usability. Among older adults, who may vary substantially in physical functioning, risk perception, and mobility, the same physical environment may not translate into the same behavioural opportunities. Objective and perceived measures may therefore capture different dimensions of the environment–behaviour relationship.

However, the stronger associations observed for perceived environments should not be interpreted as indicating that perceived measures are inherently superior to objective measures. Because many LTPA outcomes were also self-reported, associations may be affected by common method bias when both environmental exposures and physical activity are reported by the same respondent within the same survey (68). A more plausible interpretation is that objective environmental measures and subjective perceptions capture different aspects of environmental provision and individual environmental experience. Future research should therefore consider incorporating both types of measures rather than substituting one for the other. The potential influence of common method bias on associations between exposures and outcomes derived from the same information source has been widely discussed in the methodological literature on behavioural research.

Mean age, proportion of women, and year of publication did not show stable meta-regression effects, and categorical moderators such as geographic region, spatial scale, and urbanization context likewise showed no consistent patterns. This may indicate that these study characteristics are not major effect modifiers, but it may also reflect the limited number of independent studies, uneven distributions of moderator variables, and residual heterogeneity. Meta-regression is particularly sensitive to the number of studies and the distribution of study-level characteristics; therefore, isolated statistically significant findings should not be interpreted as definitive evidence of effect modification.

Sensitivity analyses further demonstrated that statistical stability differed across outcomes. The primary CAT findings for LTW and General LTPA remained broadly consistent after outlier exclusion and leave-one-study/sample-out analyses, suggesting that the pooled estimates were not driven by a single study or a small number of extreme effect estimates. In contrast, findings for NLTW and most UNIT analyses were more sensitive to CR2 correction or the exclusion of individual studies. In particular, the NLTW-UNIT association reached statistical significance in the conventional three-level model but became non-significant after CR2 correction, suggesting that statistical uncertainty may be underestimated by conventional models when the number of independent studies is limited and individual studies contribute multiple correlated effect estimates. CR2 and related small-sample corrections were specifically developed to improve robust variance inference under conditions of limited clustering, making this discrepancy important when assessing the strength of the evidence.

Diagnostics for publication bias and small-study effects also varied across outcomes. No clear signal of small-study effects was observed for LTW-CAT, whereas General LTPA-CAT showed relatively consistent evidence of funnel plot asymmetry across the three-level Egger-type test, CR2-robust testing, and other supplementary analyses. Different degrees of funnel plot asymmetry were also observed for NLTW and several UNIT models, although these analyses were based on relatively few independent studies. Accordingly, these findings are more appropriately interpreted as evidence of potential small-study effects or funnel plot asymmetry rather than as definitive evidence of publication bias. In addition to publication bias, funnel plot asymmetry may arise from genuine heterogeneity, differences in study precision, variation in study characteristics, and other mechanisms.

### 4.3 Methodological contributions and boundaries of interpretation

One methodological strength of the present study was the use of three-level meta-analysis to explicitly account for dependence among effect estimates while retaining multiple eligible estimates contributed by the same independent study sample. Conventional meta-analysis generally assumes that included effect estimates are independent; however, studies of the community built environment frequently report multiple environmental variables, spatial scales, or effect estimates from the same sample. Selecting only a single effect estimate may result in loss of relevant information, whereas treating all estimates as independent observations may underestimate standard errors and compromise statistical inference. By distinguishing sampling error, within-study-sample variation among effect estimates, and between-study-sample variation, the three-level model provides a more appropriate framework for utilizing a greater proportion of the available information while accounting for this dependent data structure.

The use of CR2 cluster-robust inference was also more than a purely supplementary methodological procedure, as it materially affected the statistical interpretation of several findings. Some UNIT models reached statistical significance under conventional three-level estimation but became non-significant after CR2 correction, indicating that conventional models may yield relatively optimistic inference when the number of independent study clusters is limited and the exact within-study correlation structure is unknown. By applying a finite-sample correction to the cluster-robust variance estimator together with Satterthwaite approximations for the degrees of freedom, CR2 provides more reliable inference for this type of data structure when the number of clusters is small.

Another methodological feature of this study was the separate modelling of CAT and UNIT estimates as distinct statistical estimands. CAT ORs primarily represent relative comparisons between categories of exposure, whereas UNIT ORs represent the strength of association corresponding to a specified unit or increment of continuous exposure. These two effect types are therefore not statistically equivalent. Although directly pooling them would increase the number of available effect estimates, doing so could combine different estimands and reduce the interpretability of the overall OR. Accordingly, CAT estimates were treated as the primary analysis and UNIT estimates as the secondary analysis, with joint CAT–UNIT models used for sensitivity analyses. The joint analyses generally reproduced the main patterns observed in the separate models and provided no basis for interpreting the two effect scales as a single common overall OR.

The findings should nevertheless be interpreted within several important evidential boundaries. First, all included studies used cross-sectional designs, precluding determination of the temporal sequence between community built-environment exposure and LTPA. The observed findings should therefore be interpreted as associations rather than causal effects (69). Second, the evidence base for NLTW was particularly sparse. The primary NLTW-CAT model included only 3 independent study samples (k = 12 effect estimates), while the NLTW-UNIT model included 5 independent study samples (k = 33 effect estimates). In addition, some NLTW moderator analyses were based on extremely limited effective degrees of freedom, with a Satterthwaite df of approximately 1.00, providing little statistical power for detecting moderator effects. Accordingly, the NLTW findings should be considered exploratory and hypothesis-generating. Importantly, the non-significant NLTW-CAT result should not be interpreted as evidence of no association between the community built environment and neighbourhood leisure-time walking, because the very small number of contributing independent study samples substantially limits statistical power and precision. In addition, the initial search formulation was relatively narrow, which may have reduced search sensitivity. During revision, the search strategy was broadened and potentially eligible records were re-screened using the clarified LTPA criteria, resulting in a final evidence base of 34 studies.

The GRADE assessment further indicated that the certainty of evidence across outcome–built-environment domain combinations was generally low to moderate. Moderate-certainty evidence was identified for traffic safety, destination accessibility, aesthetics, parks and green spaces, and residential density for LTW and General LTPA, and for traffic safety and destination accessibility for NLTW, suggesting relatively more consistent support for these associations. In contrast, the certainty of evidence for the remaining outcome–domain combinations was rated as low, primarily reflecting uncertainty arising from limited numbers of studies, between-study heterogeneity, and imprecision of effect estimates. Therefore, even when some pooled associations reached statistical significance and remained stable under CR2-robust inference and sensitivity analyses, they should not be interpreted as established causal effects. Further prospective and longitudinal studies are needed to clarify the temporal sequence and potential causal relationships between specific built-environment attributes and LTPA among older adults.

### 4.4 Implications for community environment optimization and future research

The practical implication of the present study is not to identify a single optimal environment applicable to all forms of LTPA among older adults, but rather to suggest that community environment optimization may need to be tailored to specific types of activity. For community health-promotion initiatives aimed primarily at leisure-time walking, greater attention could be given to the landscape quality and comfort of the walking environment, as well as the accessibility of activity destinations, so that the environment not only provides basic conditions for mobility but also attracts and supports older adults in sustained participation in leisure-time walking. To promote a broader range of General LTPA, greater emphasis may be placed on parks, green spaces, and multifunctional public spaces capable of supporting walking, cycling, exercise, and social interaction. The WHO age-friendly cities framework similarly highlights the importance of safe and accessible outdoor spaces, green areas, walking facilities, and public environments suitable for older adults in supporting activity and participation in later life (70).

The stronger association observed between perceived environments and General LTPA further suggests that assessments of community environments should not rely solely on objective spatial indicators such as road density, park area, or the number of facilities. For older adults, whether environmental resources are translated into actual opportunities for physical activity also depends on their lived experiences of convenience, safety, comfort, and usability. Accordingly, community planning and age-friendly environmental modification may benefit from combining objective environmental assessments with surveys of residents’ perceptions, moving beyond the question of whether an environmental resource is physically present to whether it can actually be used by older adults. Future research should first prioritise longitudinal studies, natural experiments, and evaluations of community environmental modifications to clarify the temporal sequence between changes in environmental exposure and changes in LTPA, while more adequately addressing residential self-selection and residual confounding. Previous systematic reviews of longitudinal studies and natural experiments have suggested that, compared with cross-sectional studies, these designs can provide more informative evidence regarding the temporal relationship between the built environment and physical activity, although the available evidence remains limited (71).

Second, greater attention should be given to improving the spatial correspondence between environmental exposure assessment and the actual locations in which physical activity occurs. Using fixed residential buffers alone as proxies for environmental exposure may not fully capture the spaces that older adults actually encounter in daily life. Future studies could integrate GPS, GIS, accelerometers or other wearable devices, and activity-space approaches to simultaneously capture the locations of activities and levels of physical activity, thereby enabling more precise identification of actual environmental exposures (72). Reviews of GPS-based studies among community-dwelling older adults have also shown that location tracking has been used to assess physical activity and community participation, providing a methodological basis for further spatiotemporal matching research.

Future studies should also, wherever possible, measure objective and perceived environments within the same study to distinguish the respective roles of physical environmental provision and individual environmental experience in LTPA. For continuous built-environment exposures, researchers should clearly report measurement units, specific exposure increments, and spatial scales, and should seek to improve the comparability of effect estimates across studies. Where sufficient data are available, dose–response relationships and potential non-linear associations should also be examined rather than assuming a simple linear relationship between all built-environment indicators and physical activity. A recent systematic review of walking among older adults has suggested that residential density, land-use mix, street connectivity, green space, and other environmental factors may exhibit threshold or non-linear relationships with walking; however, evidence regarding the specific thresholds for different environmental attributes remains to be confirmed (73).

Future research should therefore move beyond asking simply which environmental factors are associated with LTPA and instead address which environmental attributes, at what exposure levels, for which types of activity and older populations, and under which spatial contexts are associated with more consistent behavioural outcomes. Such an approach would improve the behavioural specificity and spatial precision of research on the community built environment and provide a stronger foundation for evaluating the potential causal relationships between environmental modifications and physical activity among older adults.

## 5 Conclusion

This systematic review and three-level meta-analysis showed that more favourable community built environments were generally associated with higher levels of LTW and General LTPA among older adults. For both outcomes, the primary CAT findings remained consistent after CR2-robust inference and multiple sensitivity analyses. In contrast, the evidence for NLTW was based on only a small number of independent study samples and should therefore be regarded as hypothesis-generating rather than as an established effect. The non-significant NLTW findings should likewise not be interpreted as evidence of no association. Most secondary UNIT analyses also showed lower statistical stability. Domain-specific analyses identified aesthetics, destination accessibility, and parks and green spaces as candidate environmental factors warranting further investigation; however, the associations between specific environmental attributes and different types of LTPA were not fully consistent.

Overall, the findings support a degree of behaviour- and context-specificity in the relationship between the community built environment and LTPA among older adults, suggesting that associations between particular environmental attributes and LTPA may vary according to the type of activity and the setting in which it occurs. Future research and community-based health-promotion practice should therefore avoid relying on a single, universally applicable environmental framework and instead place greater emphasis on matching environmental attributes with specific activity types and real-world behavioural contexts.

Because all available evidence was derived from cross-sectional studies, and because some outcomes were characterised by substantial heterogeneity, small-study effects, measurement differences, and limited numbers of independent studies, the present findings should be interpreted as evidence of association rather than causal effects. Future longitudinal studies, natural experiments, and evaluations of community environmental modifications, combined with objective environmental measures, perceived environmental assessments, and measurements of actual activity spaces, are needed to clarify which community environmental attributes show more consistent associations with LTPA among older adults under specific behavioural and spatial contexts.

## Data Availability

The original contributions presented in the study are included in the article/Supplementary material, further inquiries can be directed to the corresponding author.
