# Supplementary material for: Associations between community built environment and leisure-time physical activity among older adults: a systematic review and meta-analysis
Source: Front Public Health. 2026 Sep 29;14:1956100. doi: 10.3389/fpubh.2026.1956100 (PMC13660755; doi:10.3389/fpubh.2026.1956100)
Supplement: Supplementary file 1 [file Data_Sheet_1.PDF]

Supplementary Material

Table of Contents

Supplementary Methods

Supplementary Method S1. Search strategy of PubMed, Embase, Cochrane Library, Web of Science, Scopus, and SPORTDiscus via EBSCOhost.
Supplementary Method S2. Definitions and decision rules for built-environment domains, leisure-time physical activity outcome categories, and effect-scale classifications.
Supplementary Method S3. Effect-size processing, direction harmonization, and calculation of sampling variances.
Supplementary Method S4. Key statistical terms and interpretative framework used in the three-level meta-analysis.

Supplementary Tables

Supplementary Table S1. Study characteristics of the included studies.
Supplementary Table S2. Operational definitions, measurement rules, and analytic handling of community built-environment domains, leisure-time physical activity outcomes, and effect-scale classifications.
Supplementary Table S3. JBI methodological quality assessment of included studies.
Supplementary Table S4. Main pooled effects, heterogeneity estimates, prediction intervals, CR2 robust inference, and model-structure comparisons for LTW, NLTW, and General LTPA.
Supplementary Table S5. Subgroup analyses for LTW, NLTW, and General LTPA by built-environment domain and study characteristics.
Supplementary Table S6. Meta-regression analyses of female percentage, mean age, and publication year for LTW, NLTW, and General LTPA.
Supplementary Table S7. Publication-bias and small-study-effect diagnostics for LTW, NLTW, and General LTPA.
Supplementary Table S8. Comparison of original and outlier-removed pooled effects for LTW, NLTW, and General LTPA.
Supplementary Table S9. Leave-one-study/sample-out sensitivity analyses for LTW, NLTW, and General LTPA.
Supplementary Table S10. CAT–UNIT joint sensitivity analyses for LTW, NLTW, and General LTPA.
Supplementary Table S11. GRADE assessment of the certainty of evidence

Supplementary Figures

Supplementary Figure S1. PRISMA flow diagram of the study-selection process.
Supplementary Figure S2. Methodological quality assessment of the included studies using the JBI critical appraisal checklist.
Supplementary Figure S3. Leisure-time walking (LTW): overall three-level meta-analysis plots.
Supplementary Figure S4. Leisure-time walking (LTW): subgroup analyses.
Supplementary Figure S5. Leisure-time walking (LTW): meta-regression plots.
Supplementary Figure S6. Leisure-time walking (LTW): publication-bias and small-study-effect diagnostics.
Supplementary Figure S7. Leisure-time walking (LTW): sensitivity analyses.
Supplementary Figure S8. Leisure-time walking (LTW): CAT-UNIT joint sensitivity analysis.
Supplementary Figure S9. Neighbourhood leisure-time walking (NLTW): overall three-level meta-analysis plots.
Supplementary Figure S10. Neighbourhood leisure-time walking (NLTW): subgroup analyses.
Supplementary Figure S11. Neighbourhood leisure-time walking (NLTW): meta-regression plots.
Supplementary Figure S12. Neighbourhood leisure-time walking (NLTW): publication-bias and small-study-effect diagnostics.
Supplementary Figure S13. Neighbourhood leisure-time walking (NLTW): sensitivity analyses.
Supplementary Figure S14. Neighbourhood leisure-time walking (NLTW): CAT-UNIT joint sensitivity analysis.
Supplementary Figure S15. General leisure-time physical activity (General LTPA): overall three-level meta-analysis plots.
Supplementary Figure S16. General leisure-time physical activity (General LTPA): subgroup analyses.
Supplementary Figure S17. General leisure-time physical activity (General LTPA): meta-regression plots.
Supplementary Figure S18. General leisure-time physical activity (General LTPA): publication-bias and small-study-effect diagnostics.
Supplementary Figure S19. General leisure-time physical activity (General LTPA): sensitivity analyses.
Supplementary Figure S20. General leisure-time physical activity (General LTPA): CAT-UNIT joint sensitivity analysis.

Supplementary Methods S1. Search strategy of PubMed, Embase, Cochrane Library, Web of Science, Scopus, and SPORTDiscus via EBSCOhost.

Database	Search Terms		
	Population	Community built environment	Physical activity / LTPA
PubMed [MeSH Terms / Title/Abstract]	("Aged"[MeSH Terms] OR "Aged, 80 and over"[MeSH Terms] OR "Frail Elderly"[MeSH Terms] OR aged[Title/Abstract] OR "older adult*"[Title/Abstract] OR "older people"[Title/Abstract] OR "older person*"[Title/Abstract] OR elderly[Title/Abstract] OR senior*[Title/Abstract] OR geriatric*[Title/Abstract])	("Built Environment"[MeSH Terms] OR "built environment*"[Title/Abstract] OR "community built environment*"[Title/Abstract] OR "urban built environment*"[Title/Abstract] OR "neighborhood environment*"[Title/Abstract] OR "neighbourhood environment*"[Title/Abstract] OR "community environment*"[Title/Abstract] OR "residential environment*"[Title/Abstract] OR walkab*[Title/Abstract] OR "Neighborhood Environment Walkability Scale"[Title/Abstract] OR "Neighbourhood Environment Walkability Scale"[Title/Abstract] OR NEWS[Title/Abstract] OR "traffic safety"[Title/Abstract] OR "traffic danger*"[Title/Abstract] OR "road safety"[Title/Abstract] OR "destination access*"[Title/Abstract] OR "access to destination*"[Title/Abstract] OR "environmental safety"[Title/Abstract] OR "neighborhood safety"[Title/Abstract] OR "neighbourhood safety"[Title/Abstract] OR "crime safety"[Title/Abstract] OR aesthetic*[Title/Abstract] OR park*[Title/Abstract] OR "green space*"[Title/Abstract] OR greenspace*[Title/Abstract] OR "public open space*"[Title/Abstract] OR "recreational facilit*"[Title/Abstract] OR "residential density"[Title/Abstract] OR "population density"[Title/Abstract] OR "land use mix"[Title/Abstract] OR "land-use mix"[Title/Abstract] OR "mixed land use"[Title/Abstract] OR "street connect*"[Title/Abstract] OR "intersection density"[Title/Abstract] OR sidewalk*[Title/Abstract] OR pavement*[Title/Abstract] OR "pedestrian infrastructure"[Title/Abstract] OR crosswalk*[Title/Abstract])	("Exercise"[MeSH Terms] OR "Walking"[MeSH Terms] OR "Motor Activity"[MeSH Terms] OR "Leisure Activities"[MeSH Terms] OR exercise*[Title/Abstract] OR "physical activit*"[Title/Abstract] OR "leisure-time physical activit*"[Title/Abstract] OR "leisure time physical activit*"[Title/Abstract] OR "leisure physical activit*"[Title/Abstract] OR "recreational physical activit*"[Title/Abstract] OR walk*[Title/Abstract] OR "leisure walk*"[Title/Abstract] OR "recreational walk*"[Title/Abstract] OR "walking for exercise"[Title/Abstract] OR "exercise walking"[Title/Abstract] OR "neighborhood walk*"[Title/Abstract] OR "neighbourhood walk*"[Title/Abstract] OR cycl*[Title/Abstract] OR bicycl*[Title/Abstract] OR "recreational cycl*"[Title/Abstract] OR "leisure-time cycl*"[Title/Abstract] OR sport*[Title/Abstract] OR recreation*[Title/Abstract] OR ambulation[Title/Abstract])
Embase [Title/Abstract/Keywords]	('aged'/exp OR 'aged':ti,ab,kw OR 'older adult*':ti,ab,kw OR 'older people':ti,ab,kw OR 'older person*':ti,ab,kw OR elderly:ti,ab,kw OR senior*:ti,ab,kw OR geriatric*:ti,ab,kw)	('built environment'/exp OR 'built environment*':ti,ab,kw OR 'community built environment*':ti,ab,kw OR 'urban built environment*':ti,ab,kw OR 'neighborhood environment*':ti,ab,kw OR 'neighbourhood environment*':ti,ab,kw OR 'community environment*':ti,ab,kw OR 'residential environment*':ti,ab,kw OR walkab*:ti,ab,kw OR 'neighborhood environment walkability scale':ti,ab,kw OR 'neighbourhood environment walkability scale':ti,ab,kw OR NEWS:ti,ab,kw OR 'traffic safety':ti,ab,kw OR 'traffic danger*':ti,ab,kw OR 'road safety':ti,ab,kw OR 'destination access*':ti,ab,kw OR 'access to destination*':ti,ab,kw OR 'environmental safety':ti,ab,kw OR 'neighborhood safety':ti,ab,kw OR 'neighbourhood safety':ti,ab,kw OR 'crime safety':ti,ab,kw OR aesthetic*:ti,ab,kw OR park*:ti,ab,kw OR 'green space*':ti,ab,kw OR greenspace*:ti,ab,kw OR 'public open space*':ti,ab,kw OR 'recreational facilit*':ti,ab,kw OR 'residential density':ti,ab,kw OR 'population density':ti,ab,kw OR 'land use mix':ti,ab,kw OR 'land-use mix':ti,ab,kw OR 'mixed land use':ti,ab,kw OR 'street connect*':ti,ab,kw OR 'intersection density':ti,ab,kw OR sidewalk*:ti,ab,kw OR pavement*:ti,ab,kw OR 'pedestrian infrastructure':ti,ab,kw OR crosswalk*:ti,ab,kw)	('physical activity'/exp OR 'exercise'/exp OR 'walking'/exp OR 'leisure'/exp OR exercise*:ti,ab,kw OR 'physical activit*':ti,ab,kw OR 'leisure-time physical activit*':ti,ab,kw OR 'leisure time physical activit*':ti,ab,kw OR 'leisure physical activit*':ti,ab,kw OR 'recreational physical activit*':ti,ab,kw OR walk*:ti,ab,kw OR 'leisure walk*':ti,ab,kw OR 'recreational walk*':ti,ab,kw OR 'walking for exercise':ti,ab,kw OR 'exercise walking':ti,ab,kw OR 'neighborhood walk*':ti,ab,kw OR 'neighbourhood walk*':ti,ab,kw OR cycl*:ti,ab,kw OR bicycl*:ti,ab,kw OR 'recreational cycl*':ti,ab,kw OR 'leisure-time cycl*':ti,ab,kw OR sport*:ti,ab,kw OR recreation*:ti,ab,kw OR ambulation:ti,ab,kw)
Cochrane Library [Title/Abstract/Keywords]	(MeSH descriptor: [Aged] explode all trees OR ("older adult*" OR "older people" OR "older person*" OR elderly OR senior* OR geriatric*):ti,ab,kw)	(MeSH descriptor: [Built Environment] explode all trees OR ("built environment*" OR "community built environment*" OR "urban built environment*" OR "neighborhood environment*" OR "neighbourhood environment*" OR "community environment*" OR "residential environment*" OR walkab* OR "Neighborhood Environment Walkability Scale" OR "Neighbourhood Environment Walkability Scale" OR NEWS OR "traffic safety" OR "traffic danger*" OR "road safety" OR "destination access*" OR "access to destination*" OR "environmental safety" OR "neighborhood safety" OR "neighbourhood safety" OR "crime safety" OR aesthetic* OR park* OR "green space*" OR greenspace* OR "public open space*" OR "recreational facilit*" OR "residential density" OR	(MeSH descriptor: [Exercise] explode all trees OR MeSH descriptor: [Walking] explode all trees OR MeSH descriptor: [Motor Activity] explode all trees OR MeSH descriptor: [Leisure Activities] explode all trees OR (exercise* OR "physical activit*" OR "leisure-time physical activit*" OR "leisure time physical activit*" OR "leisure physical activit*" OR "recreational physical activit*" OR walk* OR "leisure walk*" OR "recreational walk*" OR "walking for exercise" OR "exercise

Supplementary Methods S1. Search strategy of PubMed, Embase, Cochrane Library, Web of Science, Scopus, and SPORTDiscus via EBSCOhost.			
**Database**	**Search Terms**		
	**Population**	**Community built environment**	**Physical activity / LTPA**
		"population density" OR "land use mix" OR "land-use mix" OR "mixed land use" OR "street connect*" OR "intersection density" OR sidewalk* OR pavement* OR "pedestrian infrastructure" OR crosswalk*):ti,ab,kw)	walking" OR "neighborhood walk*" OR "neighbourhood walk*" OR cycl* OR bicycl* OR "recreational cycl*" OR "leisure-time cycl*" OR sport* OR recreation* OR ambulation):ti,ab,kw)
Web of Science [Topic]	TS=("older adult*" OR "older people" OR "older person*" OR elderly OR aged OR senior* OR geriatric*)	TS=("built environment*" OR "community built environment*" OR "urban built environment*" OR "neighborhood environment*" OR "neighbourhood environment*" OR "community environment*" OR "residential environment*" OR walkab* OR "Neighborhood Environment Walkability Scale" OR "Neighbourhood Environment Walkability Scale" OR NEWS OR "traffic safety" OR "traffic danger*" OR "road safety" OR "destination access*" OR "access to destination*" OR "environmental safety" OR "neighborhood safety" OR "neighbourhood safety" OR "crime safety" OR aesthetic* OR park* OR "green space*" OR greenspace* OR "public open space*" OR "recreational facilit*" OR "residential density" OR "population density" OR "land use mix" OR "land-use mix" OR "mixed land use" OR "street connect*" OR "intersection density" OR sidewalk* OR pavement* OR "pedestrian infrastructure" OR crosswalk*)	TS=(exercise* OR "physical activit*" OR "leisure-time physical activit*" OR "leisure time physical activit*" OR "leisure physical activit*" OR "recreational physical activit*" OR walk* OR "leisure walk*" OR "recreational walk*" OR "walking for exercise" OR "exercise walking" OR "neighborhood walk*" OR "neighbourhood walk*" OR cycl* OR bicycl* OR "recreational cycl*" OR "leisure-time cycl*" OR sport* OR recreation* OR ambulation)
Scopus [Title/Abstract/Keywords]	TITLE-ABS-KEY("older adult*" OR "older people" OR "older person*" OR elderly OR aged OR senior* OR geriatric*)	TITLE-ABS-KEY("built environment*" OR "community built environment*" OR "urban built environment*" OR "neighborhood environment*" OR "neighbourhood environment*" OR "community environment*" OR "residential environment*" OR walkab* OR "Neighborhood Environment Walkability Scale" OR "Neighbourhood Environment Walkability Scale" OR NEWS OR "traffic safety" OR "traffic danger*" OR "road safety" OR "destination access*" OR "access to destination*" OR "environmental safety" OR "neighborhood safety" OR "neighbourhood safety" OR "crime safety" OR aesthetic* OR park* OR "green space*" OR greenspace* OR "public open space*" OR "recreational facilit*" OR "residential density" OR "population density" OR "land use mix" OR "land-use mix" OR "mixed land use" OR "street connect*" OR "intersection density" OR sidewalk* OR pavement* OR "pedestrian infrastructure" OR crosswalk*)	TITLE-ABS-KEY(exercise* OR "physical activit*" OR "leisure-time physical activit*" OR "leisure time physical activit*" OR "leisure physical activit*" OR "recreational physical activit*" OR walk* OR "leisure walk*" OR "recreational walk*" OR "walking for exercise" OR "exercise walking" OR "neighborhood walk*" OR "neighbourhood walk*" OR cycl* OR bicycl* OR "recreational cycl*" OR "leisure-time cycl*" OR sport* OR recreation* OR ambulation)
SPORTDiscus (via EBSCOhost) [Title/Abstract]	TI ("older adult*" OR "older people" OR "older person*" OR elderly OR aged OR senior* OR geriatric*) OR AB ("older adult*" OR "older people" OR "older person*" OR elderly OR aged OR senior* OR geriatric*)	TI ("built environment*" OR "community built environment*" OR "urban built environment*" OR "neighborhood environment*" OR "neighbourhood environment*" OR "community environment*" OR "residential environment*" OR walkab* OR "Neighborhood Environment Walkability Scale" OR "Neighbourhood Environment Walkability Scale" OR NEWS OR "traffic safety" OR "traffic danger*" OR "road safety" OR "destination access*" OR "access to destination*" OR "environmental safety" OR "neighborhood safety" OR "neighbourhood safety" OR "crime safety" OR aesthetic* OR park* OR "green space*" OR greenspace* OR "public open space*" OR "recreational facilit*" OR "residential density" OR "population density" OR "land use mix" OR "land-use mix" OR "mixed land use" OR "street connect*" OR "intersection density" OR sidewalk* OR pavement* OR "pedestrian infrastructure" OR crosswalk*) OR AB ("built environment*" OR "community built environment*" OR "urban built environment*" OR "neighborhood environment*" OR "neighbourhood environment*" OR "community environment*" OR "residential environment*" OR walkab* OR "Neighborhood Environment Walkability Scale" OR "Neighbourhood Environment Walkability Scale" OR NEWS OR "traffic safety" OR "traffic danger*" OR "road safety" OR "destination access*" OR "access to destination*" OR "environmental safety" OR "neighborhood safety" OR "neighbourhood safety" OR "crime safety" OR aesthetic* OR park* OR "green space*" OR greenspace* OR "public open space*" OR "recreational facilit*" OR "residential density" OR	TI (exercise* OR "physical activit*" OR "leisure-time physical activit*" OR "leisure time physical activit*" OR "leisure physical activit*" OR "recreational physical activit*" OR walk* OR "leisure walk*" OR "recreational walk*" OR "walking for exercise" OR "exercise walking" OR "neighborhood walk*" OR "neighbourhood walk*" OR cycl* OR bicycl* OR "recreational cycl*" OR "leisure-time cycl*" OR sport* OR recreation* OR ambulation) OR AB (exercise* OR "physical activit*" OR "leisure-time physical activit*" OR "leisure time physical activit*" OR "leisure physical activit*" OR "recreational physical activit*" OR walk* OR "leisure walk*" OR "recreational walk*" OR "walking for exercise" OR "exercise walking" OR "neighborhood walk*" OR "neighbourhood walk*" OR cycl* OR bicycl* OR "recreational cycl*" OR "leisure-time cycl*" OR sport* OR recreation* OR ambulation)

Supplementary Methods S1. Search strategy of PubMed, Embase, Cochrane Library, Web of Science, Scopus, and SPORTDiscus via EBSCOhost.			
**Database**	**Search Terms**		
	**Population**	**Community built environment**	**Physical activity / LTPA**
		"population density" OR "land use mix" OR "land-use mix" OR "mixed land use" OR "street connect*" OR "intersection density" OR sidewalk* OR pavement* OR "pedestrian infrastructure" OR crosswalk*)	

Search logic: terms within each concept block were combined using OR; the three concept blocks (Population AND Community built environment AND Physical activity/LTPA) were combined using AND. No study-design filter was applied in order to maximize sensitivity for eligible observational studies.

Final search date: August 23, 2026.

Reference lists of included studies and relevant systematic reviews/meta-analyses were also screened manually to identify potentially eligible studies not retrieved through database searches. English-language eligibility was applied during study selection.

Supplementary Method S2

Supplementary Method S2. Definitions and decision rules for built-environment domains, leisure-time physical activity outcome categories, and effect-scale classifications.

Classification was based on the operational definition, measurement method, model coding, and context reported in the original article and, when necessary, in cited instruments or supplementary materials. Author-provided variable labels were not used in isolation when the underlying construct could be determined more precisely from the full report.

A. Built-environment domains

Category	Full name	Definition and decision rules
**TS**	Traffic safety	Built-environment characteristics related to the risk or perceived risk posed by motorized traffic to walking or other outdoor activity. Eligible indicators included traffic volume or speed, dangerous traffic, crossing safety, pedestrian signals, road-crossing difficulty, and comparable traffic-related safety measures. Crime, violence, and personal security were not classified as TS and were assigned to ES when appropriate.
**DA**	Destination accessibility	Access to destinations, services, amenities, or activity opportunities in and around the residential neighbourhood. Eligible indicators included proximity, distance, density, number, diversity, or perceived access to shops, services, public transport, community facilities, recreational destinations, and other routine destinations. When an indicator primarily represented access to a destination rather than the diversity of land uses, it was classified as DA rather than LUM.
**ES**	Environmental safety	Safety of the neighbourhood environment from non-traffic threats. Eligible indicators included perceived or objectively assessed crime, violence, personal security, fear of crime, neighbourhood security, or other non-traffic safety concerns. Traffic-related danger was classified as TS rather than ES.
**AE**	Aesthetics	Perceived or objectively assessed visual and experiential qualities of the neighbourhood that may make outdoor activity more or less appealing. Eligible indicators included attractiveness, pleasant scenery, visual interest, cleanliness, maintenance, attractive buildings or streetscapes, and comparable aesthetic qualities. Measures whose primary construct was the presence or accessibility of parks or green spaces were classified as PG.
**PG**	Parks and green spaces	Availability, proximity, accessibility, quantity, or quality of parks, public open spaces, green spaces, gardens, and comparable recreational green areas. Indicators could be objectively measured (e.g., GIS-based distance or area) or perceived. General neighbourhood aesthetics or greenery not explicitly representing a park/green-space resource were not automatically classified as PG.
**RD**	Residential density	Density of residences, dwellings, households, or population within the residential or neighbourhood area. Eligible indicators included dwelling density, housing density, residential density, population density, and comparable density measures. Density of destinations or amenities was classified according to the construct represented, most commonly DA.
**LUM**	Land-use mix	Degree of heterogeneity, diversity, or mixing of land uses within the neighbourhood. Eligible indicators included land-use mix indices, entropy measures, residential-commercial mix, diversity of land-use categories, and comparable measures of mixed use. Single-destination proximity or counts were classified as DA rather than LUM.
**SC**	Street connectivity	Configuration and connectedness of the street or route network. Eligible indicators included intersection density, street connectivity indices, route directness, block size or length, cul-de-sac prevalence, connected street networks, and comparable measures describing the ease of moving through the street network. Sidewalk condition or pedestrian-path quality was classified as SQ.
**SQ**	Sidewalk quality	Presence, continuity, coverage, width, condition, maintenance, accessibility, or perceived quality of sidewalks, footpaths, pavements, and comparable pedestrian infrastructure. Street-network configuration was classified as SC, whereas the physical quality or availability of pedestrian paths was classified as SQ.

Decision rule for ambiguous built-environment variables. When a reported variable could plausibly map to more than one domain, classification was based on the primary construct represented by the original operational definition and measurement items. The full text and cited measurement instrument were checked when required, and disagreements were resolved by reviewer consensus. A variable was not reclassified solely because its statistical label differed from the terminology used in this review.

B. Leisure-time physical activity outcome categories

Category	Full name	Definition and decision rules

Category	Full name	Definition and decision rules
**LTW**	Leisure-time walking	Walking undertaken for leisure, recreation, exercise, or similar non-transport purposes, without an explicit requirement that the activity occurred within the participant's residential neighbourhood. Examples included recreational walking, walking for exercise, and leisure walking. If the original outcome explicitly restricted walking to the neighbourhood/community, it was classified as NLTW.
**NLTW**	Neighbourhood leisure-time walking	Leisure, recreational, or exercise walking explicitly reported as occurring within the participant's neighbourhood, residential area, or comparable local community context. Neighbourhood location had to be stated in the outcome definition, measurement instrument, or analytic description. General leisure walking with no neighbourhood restriction was classified as LTW.
**General LTPA**	General leisure-time physical activity	All other eligible leisure-time physical activity outcomes that did not meet the operational definitions of LTW or NLTW. This category included leisure-time cycling, combined leisure-time walking and cycling, overall/general LTPA composed of more than one leisure activity, and leisure-time physical activity explicitly excluding walking. Mixed-purpose or broadly defined physical-activity outcomes were not automatically classified as General LTPA; they were retained only when full-text review confirmed that the outcome met the prespecified leisure-time physical-activity eligibility criteria.

Outcome classification was based on the activity purpose and setting described in the original report or source instrument. The three outcome categories were analyzed separately; effect sizes were not pooled across LTW, NLTW, and General LTPA.

C. Effect-scale classifications

Category	Full name	Definition and decision rules
**CAT**	Categorical comparison	Effect estimates based on an explicit categorical contrast with a clearly defined reference group. Eligible comparisons included presence versus absence, high versus low, better versus worse, ordered categories, tertiles/quartiles/quintiles, or other study-defined categories. When several non-reference categories were compared with the same reference group, each reported contrast was retained as a separate CAT effect estimate when otherwise eligible.
**UNIT**	Continuous unit-based estimate	Effect estimates for a continuous built-environment exposure when the increment corresponding to the reported OR was explicitly identifiable from the original analysis. Eligible scaling included per 1-unit increase, a specified physical increment (e.g., distance, density, percentage), or an explicitly stated standardized increment such as per SD or IQR. Knowing only the measurement unit of the exposure was insufficient: the exact model increment or scaling represented by the OR had to be reported or unambiguously derivable.

Analytic rule. CAT and UNIT estimates represent different estimands and were therefore modeled separately. CAT was treated as the primary effect scale, and UNIT was analyzed in separate three-level models. Only effect estimates meeting one of these two prespecified definitions were included in the quantitative synthesis. Joint CAT–UNIT models were used only as sensitivity analyses and were not interpreted as a single pooled overall OR.

Note. TS, traffic safety; DA, destination accessibility; ES, environmental safety; AE, aesthetics; PG, parks and green spaces; RD, residential density; LUM, land-use mix; SC, street connectivity; SQ, sidewalk quality; LTW, leisure-time walking; NLTW, neighbourhood leisure-time walking; LTPA, leisure-time physical activity; CAT, categorical comparison; UNIT, continuous unit-based estimate; OR, odds ratio; SD, standard deviation; IQR, interquartile range.

Supplementary Method S3

Supplementary Method S3. Effect-size processing, direction harmonization, and calculation of sampling variances.

Effect-size processing was performed to place eligible associations between community built-environment characteristics and leisure-time physical activity (LTPA) on a consistent odds-ratio (OR) scale while preserving the exposure contrast or increment reported in the original study. All extracted estimates were checked against the full article, tables, supplementary materials, and, where necessary, the cited measurement instrument before quantitative analysis.

1. Eligible effect estimates

Adjusted odds ratios (aORs) and prevalence odds ratios were preferentially extracted. When an original study reported an exponentiated regression coefficient from a logistic model, it was treated as an OR only when the model specification and coefficient interpretation were equivalent to an odds ratio for the eligible LTPA outcome. When several multivariable models were available for the same exposure-outcome contrast, the most fully adjusted model that remained conceptually appropriate for the review question was retained.

Effect estimates that could not be interpreted on an OR scale, or for which the information required to derive a log OR and its sampling variance was unavailable, were not entered into the pooled OR models. Such results were retained for supplementary or narrative description when relevant.

2. Direction harmonization

Before transformation to the logarithmic scale, the direction of each built-environment exposure was audited. The common interpretation was defined so that OR > 1 represented an association between a more favorable built environment and a higher level or greater likelihood of LTPA, whereas OR < 1 represented the opposite direction.

For exposures for which a higher numerical value represented a less favorable environment (for example, greater traffic danger, greater crime-related risk, or longer distance to a destination), the OR was inverted so that the effect estimate represented the corresponding favorable direction. The confidence limits were inverted simultaneously:

$$OR^* = 1 / OR$$

$$CI^*lower = 1 / CIupper \quad \text{and} \quad CI^*upper = 1 / CIlower$$

No inversion was applied when the original exposure coding already followed the favorable direction. Direction harmonization was based on the underlying construct and coding reported in the original analysis rather than on the variable name alone. For categorical comparisons, the reference and comparison categories were checked directly; for continuous variables, the sign and meaning of the reported increment were verified.

3. Transformation to log odds ratios and sampling variances

All eligible ORs were analyzed on the natural-logarithmic scale. For each effect estimate i, the effect size was:

$$yi = \ln(ORi)$$

When a 95% confidence interval was reported, the standard error (SE) of the log OR was calculated from the confidence limits:

$$SEi = [\ln(CIupper) - \ln(CIlower)] / (2 \times 1.96)$$

$$SEi = [\ln(CIupper) - \ln(CIlower)] / 3.92$$

The corresponding Level-1 sampling variance was:

$$vi = SEi^2$$

If a standard error or other directly usable variance measure was reported by the original study, the reported value was used after confirming that it referred to the same OR estimate. Meta-analytic models were fitted on the log-OR scale, and pooled coefficients and interval estimates were exponentiated back to the OR scale for reporting.

4. Handling multiple eligible estimates from the same independent study sample

A single independent study sample could contribute more than one eligible effect estimate because studies frequently reported multiple built-environment domains, different spatial buffers, multiple exposure definitions, or more than one eligible outcome threshold. Distinct eligible estimates were retained rather than selecting a single effect arbitrarily, provided that each estimate represented a clearly identifiable exposure-outcome contrast.

All effect estimates were linked to their independent study sample so that within-sample dependence could be handled in the three-level random-effects model. Level 1 represented known sampling error, Level 2 represented heterogeneity among multiple effect estimates within the same independent study sample, and Level 3 represented heterogeneity between independent study samples. Cluster-robust variance estimation with the CR2 small-sample correction was additionally used at the independent study-sample level to assess the robustness of inference to residual within-sample dependence.

5. Effect-scale classification and analytic handling

After direction auditing and effect-size calculation, estimates were classified as CAT or UNIT according to the definitions and decision rules in Supplementary Method S2.

CAT estimates represented explicit categorical contrasts with a defined reference group. Each eligible contrast was retained as a separate effect estimate when the original study reported more than one non-reference category.

UNIT estimates represented continuous-exposure effects for which the increment corresponding to the reported OR was explicitly stated or could be unambiguously derived. The original scaling (for example, per unit, per specified distance or density increment, per standard deviation, or per interquartile range) was recorded as reported. No attempt was made to impose a common physical unit across conceptually different built-environment measures.

Because CAT and UNIT estimates represent different estimands, they were analyzed in separate three-level models. CAT was treated as the primary effect scale and UNIT as a separate secondary quantitative analysis. Joint CAT-UNIT

models were used only as sensitivity analyses to assess consistency across effect scales and were not interpreted as a single pooled overall OR.

6. Outcome-specific data organization

Effect-size datasets were organized separately for leisure-time walking (LTW), neighbourhood leisure-time walking (NLTW), and General LTPA. An effect estimate was assigned to only the outcome category supported by the operational definition in the original report. No pooled effect was calculated across the three outcome categories.

Where the same article contributed estimates to more than one eligible outcome category, the estimates were retained in their respective outcome-specific datasets while preserving the shared independent study-sample structure for clustering and sensitivity analyses.

7. Quality-control checks before meta-analysis

Before model fitting, all eligible rows were checked for internal consistency among the reported OR, confidence limits, exposure direction, effect-scale classification, and outcome category. Log ORs and sampling variances were recalculated programmatically from the final harmonized ORs and confidence intervals. Records with non-positive ORs or confidence limits, reversed confidence limits that could not be resolved from the source article, duplicate effect estimates, or insufficient information for variance calculation were flagged for manual review before inclusion.

Effect-size processing and subsequent three-level meta-analytic procedures were conducted in R. The analysis dataset retained identifiers for the independent study sample and individual effect estimate, the built-environment domain, original exposure definition, harmonized OR and 95% confidence interval, log OR, standard error, sampling variance, effect-scale classification, and LTPA outcome category to ensure complete traceability from the source article to the final model.

Note. OR, odds ratio; aOR, adjusted odds ratio; CI, confidence interval; SE, standard error; LTPA, leisure-time physical activity; LTW, leisure-time walking; NLTW, neighbourhood leisure-time walking; CAT, categorical comparison; UNIT, continuous unit-based estimate; CR2, cluster-robust variance estimator with small-sample correction.

Supplementary Method S4

Supplementary Method S4. Key statistical terms and interpretative framework used in the three-level meta-analysis

Term	Explanation
**Odds ratio (OR)**	The odds ratio was the principal effect measure. After direction harmonization, OR > 1 indicated that a more favorable community built environment was associated with a higher level or greater likelihood of leisure-time physical activity (LTPA), whereas OR < 1 indicated the opposite direction. OR = 1 represented no association.
**Log odds ratio (log OR)**	Eligible ORs were transformed to the natural-logarithmic scale before model fitting because the log OR has more suitable statistical properties for meta-analysis. Sampling variances were calculated on this scale. Model estimates and interval limits were exponentiated back to the OR scale for presentation and interpretation.
**Direction of effect**	Exposure directions were harmonized so that a higher value consistently represented a more favorable built environment. For variables in which higher values denoted a less favorable condition, such as greater traffic danger, greater crime-related risk, or longer distance to a destination, the OR and its confidence limits were inverted before analysis.
**95% confidence interval (CI)**	The 95% CI quantifies uncertainty around the pooled mean effect. On the OR scale, an interval that does not include 1 is conventionally regarded as statistically significant at the two-sided 0.05 level. The CI describes uncertainty in the average pooled association and is distinct from the prediction interval.
**p value and statistical significance**	Statistical tests were two-sided and $p < 0.05$ was considered statistically significant. P values were interpreted together with effect magnitude, 95% CIs, prediction intervals, heterogeneity, CR2 robust inference, and sensitivity analyses rather than being used as the sole criterion for interpretation.
**CAT effect scale**	CAT refers to categorical exposure comparisons with an explicit reference group, such as presence versus absence, high versus low, better versus worse, or study-defined ordered categories. CAT estimates were treated as the primary quantitative effect scale and modeled separately from UNIT estimates because the two scales represent different estimands.
**UNIT effect scale**	UNIT refers to continuous built-environment estimates for which the increment represented by the reported OR was explicitly stated or could be unambiguously derived. The original scaling was retained as reported. UNIT estimates were analyzed in separate three-level models and were not directly pooled with CAT estimates in the primary analyses.
**Three-level meta-analysis**	A three-level random-effects model was used because an independent study sample could contribute multiple eligible effect estimates, for example across several built-environment domains, spatial scales, exposure definitions, or outcome thresholds. The model separates known sampling error, within-sample heterogeneity among multiple effect estimates, and between-sample heterogeneity across independent study samples.
**Level 1 variance**	Level 1 represents the known sampling variance of each observed log OR. This component reflects uncertainty caused by estimating an association from a finite sample and is calculated from the standard error of the individual effect estimate.
**Level 2 variance (within-study/sample heterogeneity)**	Level 2 represents heterogeneity among multiple effect estimates derived from the same independent study sample. Such variation may arise from different built-environment domains, spatial buffers, exposure operationalizations, or outcome definitions reported within the same sample.
**Level 3 variance (between-study/sample heterogeneity)**	Level 3 represents heterogeneity in true associations across independent study samples. This component may reflect differences in study populations, geographic contexts, built-environment measurement, spatial scale, outcome assessment, study design, or other study-level characteristics.
**Variance components (tau-squared, τ^2)**	The three-level model estimates separate random-effects variance components for Level 2 and Level 3. Larger τ^2 values indicate greater heterogeneity at the corresponding level. The variance components were estimated using restricted maximum likelihood in the primary random-effects models.
**Heterogeneity**	Heterogeneity refers to variability in observed associations beyond that expected from sampling error alone. It was evaluated using Cochran's Q statistic, model-based variance components, and I^2 statistics, with particular attention to the decomposition of heterogeneity across Levels 2 and 3.
**Cochran's Q statistic**	The Q statistic evaluates whether the dispersion of observed effect estimates exceeds that expected from sampling error alone. A statistically significant Q value suggests heterogeneity, but the test is sensitive to the number of effect estimates and was interpreted jointly with I^2 and the estimated variance components.
**I^2 statistic**	I^2 expresses the proportion of total observed variability attributable to heterogeneity rather than sampling error. Conventional reference values of approximately 25%, 50%, and 75% may be described as low, moderate, and substantial heterogeneity, respectively, but interpretation should consider the number of studies, effect sizes, and research context.
**Level 2 and Level 3 I^2**	In the three-level framework, total heterogeneity was decomposed into Level 2 and Level 3 components. Level 2 I^2 reflects variability among multiple effect estimates within the same independent study sample, whereas Level 3 I^2 reflects variability between independent study samples.
**95% prediction interval (PI)**	The 95% PI estimates the range in which the true association from a future comparable study may plausibly fall. Unlike the 95% CI, which quantifies uncertainty around the pooled mean association, the PI incorporates heterogeneity and therefore provides information about the expected dispersion of true effects across settings.

Term	Explanation
**Restricted maximum likelihood (REML)**	REML was used to estimate random-effects variance components in the primary three-level models. REML is commonly preferred for random-effects meta-analysis because it generally provides comparatively stable and less biased heterogeneity estimates than simple maximum-likelihood estimation.
**Maximum likelihood (ML)**	ML estimation was used when comparing nested model structures. The full three-level model and corresponding simplified models were refitted using ML so that likelihood-based comparisons were valid.
**Likelihood-ratio test (LRT)**	The LRT was used to compare nested models and assess whether retaining additional random-effects levels materially improved model fit. The test was considered together with Akaike information criterion (AIC) and Bayesian information criterion (BIC), rather than as the sole determinant of model adequacy.
**Akaike information criterion (AIC) and Bayesian information criterion (BIC)**	AIC and BIC are comparative model-fit indices that balance model fit against complexity. Lower values indicate a more favorable trade-off between fit and parsimony when comparing models fitted to the same data under the same estimation framework.
**Cluster-robust variance estimation (CR2)**	CR2 robust variance estimation was used to obtain standard errors, confidence intervals, and hypothesis tests that are less sensitive to misspecification of the covariance structure among multiple effect estimates from the same independent study sample. The independent study sample served as the clustering unit.
**Satterthwaite approximation**	The Satterthwaite approximation was used with CR2 inference to obtain small-sample-adjusted degrees of freedom for t and F tests. This correction is particularly important when the number of independent study-sample clusters is limited.
**Subgroup analysis**	Subgroup analyses examined whether pooled associations differed across categorical moderators, including built-environment domain and other study characteristics when the available data were sufficient. Between-group differences were evaluated using moderator tests rather than by comparing whether individual subgroup estimates were separately statistically significant.
**Meta-regression**	Meta-regression examined whether continuous study-level characteristics were associated with variation in effect estimates. Mean age, percentage of female participants, and publication year were considered when data availability and the number of independent study samples were adequate. Regression coefficients describe the estimated change in log OR associated with a one-unit increase in the moderator.
**Moderator analysis**	Moderator analysis refers collectively to subgroup analyses and meta-regression used to explore potential sources of heterogeneity. Because these analyses are observational at the study level and may have limited power, they were interpreted as exploratory rather than causal.
**Publication bias and small-study effects**	Publication bias refers to selective publication related to study results, whereas small-study effects describe a broader association between effect magnitude and study precision. Funnel-plot asymmetry may arise from publication bias but may also reflect heterogeneity, methodological differences, or other sources of asymmetry.
**Contour-enhanced funnel plot**	Contour-enhanced funnel plots display effect estimates against precision and superimpose regions corresponding to conventional statistical-significance thresholds. These contours assist in distinguishing asymmetry potentially compatible with publication bias from asymmetry that may be related to heterogeneity or other study characteristics.
**Egger-type regression**	Egger-type regression examines whether effect estimates are associated with their standard errors or sampling variances. In this review, Egger-type tests were fitted within the three-level framework where appropriate and were supplemented by CR2 robust inference at the independent study-sample level. A significant result was interpreted as evidence of small-study effects or asymmetry, not as definitive proof of publication bias.
**Trim-and-fill method**	Trim-and-fill was used as a supplementary diagnostic to estimate potentially missing effect estimates under assumptions of funnel-plot asymmetry and to examine the corresponding adjusted pooled estimate. Because the conventional method does not explicitly model the three-level dependence structure and relies on strong assumptions, results were treated as exploratory sensitivity evidence.
**Sensitivity analysis**	Sensitivity analyses evaluated whether the main findings were robust to influential observations and analytic choices. These analyses included outlier removal, leave-one-study/sample-out analyses, and joint CAT-UNIT sensitivity models.
**Outlier analysis**	Potential outlying effect estimates were identified using studentized residuals from the three-level model. Estimates with an absolute studentized residual greater than 1.96 were flagged for sensitivity analysis. Models were refitted after their exclusion and changes in pooled estimates, confidence intervals, prediction intervals, and heterogeneity were examined.
**Leave-one-study/sample-out analysis**	One independent study sample and all effect estimates contributed by that sample were removed at a time, after which the three-level model was refitted. This procedure assessed whether the pooled association depended disproportionately on any single independent data source and preserved the clustering structure more appropriately than deleting one effect estimate at a time.
**CAT-UNIT joint sensitivity analysis**	CAT and UNIT estimates were entered together only in sensitivity models with effect scale included as a moderator. Shared-heterogeneity and scale-specific-heterogeneity specifications were examined, with CR2 robust inference. The purpose was to evaluate whether the separate CAT and UNIT analyses yielded consistent directions and inferences, not to obtain a single pooled overall OR across incompatible estimands.
**Methodological quality assessment**	Methodological quality was assessed using the Joanna Briggs Institute critical appraisal tool for analytical cross-sectional studies. Eight domains covering participant selection and description, validity and reliability of exposure and outcome measurement, identification and handling of confounding, and appropriateness of statistical analysis were evaluated.

Term	Explanation
**Forest plot**	Forest plots were used to display individual study-level or effect-level OR estimates with corresponding 95% CIs and, where appropriate, pooled estimates. They provide a transparent view of the direction, magnitude, and precision of the contributing associations.
**Orchard plot**	Orchard plots were used to provide compact visual summaries of pooled estimates and moderator results from the three-level models. These plots can display pooled ORs together with 95% CIs and 95% PIs and are particularly useful when multiple subgroup estimates or dependent effect sizes are presented.

Note. OR, odds ratio; CI, confidence interval; PI, prediction interval; LTPA, leisure-time physical activity; CAT, categorical comparison; UNIT, continuous unit-based estimate; REML, restricted maximum likelihood; ML, maximum likelihood; LRT, likelihood-ratio test; AIC, Akaike information criterion; BIC, Bayesian information criterion; CR2, cluster-robust variance estimator with small-sample correction; JBI, Joanna Briggs Institute.

Supplementary Table S1 – Study characteristics of the included studies.

Study-level					Participants				Built-environment characteristics			LTPA / walking outcome			Analysis	
ID	Study (year)	Country	Design	Cohort / data source	N	Age (mean [SD])	Female (%)	Setting	BE domain(s)	BE assessment	Spatial scale	Outcome category	PA measure	Outcome definition	Effect	Adjusted covariates
1	**Booth 2000**	Australia	Cross-sectional	NR	402	60+	55.2	NR	PG;SQ;DA;ES	Perc./Q	NR	General LTPA	Q	Sufficiently active vs inactive	aOR	Age; sex; birthplace; marital; employ.; living
2	**Berke 2007**	USA	Cross-sectional	ACT	936	78.5±6.1	64.2	Mixed	DA;RD;LUM;SC	Obj./GIS	100/500/1000 m	LTW	Q	Any exercise walking vs none	aOR	Age; edu.; income; depression; living; chronic dz; arthritis; smoking
3	**Sugiyama 2008**	UK	Cross-sectional	NR	268	75.0±7.2	60.8	Mixed	PG;AE;SQ;ES;DA	Perc./Q	Self	LTW	Q	Recr. walk >1 h/wk vs ≤1 h	aOR	Age; sex; edu.; former occupation
4	**Salvador 2009**	Brazil	Cross-sectional	NR	385	60+	60.5	Urban	DA;ES;PG	Perc./NEWS	Self	LTW	IPAQ-LF	LTPA ≥150 min/wk; walking predominant	aOR	Education; sex-stratified models
5	**Gómez 2010**	Colombia	Cross-sectional	BEOAP	1,966	70.7±7.7	62.5	Urban	PG;SC;TS;SQ;DA	Obj.+Perc./GIS+Q	500 m	LTW	Q	Walking ≥60 / ≥150 min/wk; leisure + utilitarian combined	aPOR	Age; sex; edu.; residence yrs; family proximity; slope; all GIS/perceived env. variables
6	**Parra 2010**	Colombia	Cross-sectional	BEOAP	1,966	70.7±7.7	62.5	Urban	PG;SC;LUM	Obj./GIS	500 m	General LTPA	Q	Active park use vs no	aPOR	Sex; age; edu.; PA limitation; slope; all GIS vars
7	**Satariano 2010**	USA	Cross-sectional	HAN Walking Study	884	≥65	76.6	Mixed	RD;LUM;SC;SQ;AE;TS;ES;DA	Obj.+Perc./GIS+NEWS	400 m; Self	LTW	Q	Walking <150 vs ≥150 min/wk	aOR	Site; age; sex; race; income; edu.; lower-body function; car access
8	**Wang 2010**	USA	Cross-sectional	NR	114	84.2 (61–100)	81.6	Senior-living	DA;SC;SQ;AE;PG	Obj.+Perc./GIS+Q	0.25/0.5/1/2 mi	NLTW	Q	≥1 time/day; ≥10 min/occurrence	aOR	Age; edu.; marital; income; health; mobility; self-efficacy; social/living factors
9	**Hanibuchi 2011**	Japan	Cross-sectional	AGES	9,414	65+	52.0	Mixed	RD;SC;DA;PG	Obj./GIS	250/500/1000 m	General LTPA	Q	Leisure sports frequency categories	aOR	Age; sex; marital; edu.; income; work; health; IADL; location
10	**Inoue 2011**	Japan	Cross-sectional	NR	1,921	69.5±2.9	48.1	Mixed	RD;DA;SQ;TS;ES;AE	Perc./Q	Self	LTW	Q	Recr. walking ≥30 min/wk vs <30	aOR	Age; sex; city; work; edu.; BMI; self-rated health
11	**Boruff 2012**	Australia	Cross-sectional	Active Living Study	325	76.8±7.4	68.0	Urban	PG	Obj./GIS	1 km	LTW	CHAMPS	Any leisure walking vs none	aOR	Age; sex; edu.; marital; BMI; physical function; clustering
12	**Nathan 2012**	Australia	Cross-sectional	WAHWSS	2,918	72.9±5.4	55.9	Urban	DA;LUM;SC	Obj./GIS	400/800 m	LTW	Active Australia Survey	Weekly walking: none vs some; <150 vs ≥150 min/wk; recreation/exercise + transport	aOR	Age; sex; edu.; marital; self-rated health; assistive equipment; street connectivity
13	**Pelclová 2012**	Czech/Poland/Slovakia	Cross-sectional	NR	456	55+	88.2	Urban	RD;LUM;DA;SC;AE;TS;ES;SQ	Perc./NEWS	Self	LTW	IPAQ	≥30 min ×5 d/wk	OR	NR
14	**Tsunoda 2012**	Japan	Cross-sectional	Kasama Study	421	73.3±5.3	52.5	Rural	RD;DA;SQ;TS;ES;AE	Perc./Q	NR	LTW; General LTPA	PASE	LTPA except walking: any/sufficient vs inactive/insufficient	aOR	Age; sex; edu.; work; heart/stroke; low-back/knee/hip dz

Study-level					Participants				Built-environment characteristics			LTPA / walking outcome			Analysis	
ID	Study (year)	Country	Design	Cohort / data source	N	Age (mean [SD])	Female (%)	Setting	BE domain(s)	BE assessment	Spatial scale	Outcome category	PA measure	Outcome definition	Effect	Adjusted covariates
15	**Van Cauwenberg 2012**	Belgium	Cross-sectional	Belgian Aging Studies	48,879	74.4±6.7	55.7	Mixed	RD;DA;ES;TS;AE;PG;SQ	Obj.+Perc.	Admin.;Self	General LTPA	Q	Recr. walk/cycle ≥1×/wk	aOR	Edu.; functional limitations
16	**Cerin 2013a**	Hong Kong, China	Cross-sectional	NR	484	65–74/75+	NR	Urban	PG;DA;SQ;ES;TS;AE	Obj./Audit	400 m	LTW; General LTPA	IPAQ	Recr. walk min/wk; NW-LTPA participation/min	aOR;eβ	Age; sex; edu.; area SES; audited segments; clustering; env. covariates
17	**Cerin 2013b**	Hong Kong, China	Cross-sectional	NR	484	65–74/75+	NR	Urban	DA;SQ;ES;TS;AE	Obj./Audit	400 m	NLTW	NWQ-CS	Within-neigh. recreational walking participation / % of walking for recreation	aOR;eβ	Age; sex; edu.; area SES; clustering; env. covariates
18	**Kramer 2013**	Netherlands	Cross-sectional	POLS + WoON	4,121	>65	54.6	Mixed	TS;ES;AE	Perc./Q	Admin.	LTW; General LTPA	SQUASH	Outdoor LTW/LTC ≥30 min/wk	aOR	Age; sex; ethnicity; HH comp.; edu.; income; wealth; pop. density
19	**Ding 2014**	USA	Cross-sectional	SNQLS	880	75 (66–97)	56.0	Urban	TS;DA;ES;AE;PG;RD;LUM;SC;SQ	Obj.+Perc.	500 m	LTW	CHAMPS	Leisure walk ≥1×/wk	aOR	Age; sex; edu.; race; marital; living; residence yrs; health; mobility
20	**Nathan 2014**	Australia	Cross-sectional	Active Living Study	323	76.9±7.3	68.1	Urban	AE;ES;TS;DA	Perc./NEWS	Self	LTW	CHAMPS	Leisure walk ≥150 min/wk	aOR	Age; sex; edu.; physical function; sampling; walkability; clustering
21	**Troped 2014**	USA	Cross-sectional	NHS	23,434	70.0±6.9	100	Mixed	RD;SC;DA	Obj./GIS	800/1200 m	LTW	Q	Meeting PA recommendation via walking (≥500 MET-min/wk)	aOR	Age; race/ethnicity; participant/spouse edu.; BMI; walking limits; indoor preference; years at address
22	**Gao 2015**	China	Cross-sectional	NR	2,783	60+	58.9	Urban	AE;DA	Perc./Q	Self	General LTPA	IPAQ	LTPA ≥150 min/wk	aOR	Sex; age; marital; edu.; self-rated health; chronic dz
23	**McMorris 2015**	Canada	Cross-sectional	CCHS 2001	18,474	60+	NR	Urban	PG	Obj./GIS	30/500 m	General LTPA	Q	Any LTPA in past 3 mo	aOR	Income adequacy; smoking; marital; month; longitude
24	**Procter-Gray 2015**	USA	Cross-sectional	MOBILIZE Boston	745	78.1±5.4	NR	Urban	DA;PG	Obj./GIS	NR	NLTW	Q	Neighborhood exercise walk ≥1×/wk	aOR	Personal composite; amenity distances; community SES
25	**Barnett 2016**	Hong Kong, China	Cross-sectional	ALECS	909	76±6	66.0	Urban	RD;LUM;DA;SC;ES;TS;AE;SQ	Perc./NEWS	Self	NLTW	NWQ-CS	Within-neigh. recr. walk participation/min	aOR;eβ	Socio-demo.; recruitment; chronic conditions; comorbidity
26	**Giehl 2016**	Brazil	Cross-sectional	EpiFloripa	1,667	70.4±8.0	63.9	Urban	RD;SC;SQ;LUM;PG;ES	Obj./GIS	Admin.	LTW	IPAQ	Leisure walk ≥10 min/wk vs none	aOR	Sex; age; edu.
27	**Corseuil Giehl 2017**	Brazil	Cross-sectional	EpiFloripa	1,637	70.3±7.7	63.9	Urban	SQ;TS;ES;AE;PG	Perc./NEWS	Self	LTW	IPAQ	0; 10–149; ≥150 min/wk	aOR	Sex; age; edu.; BMI; self-rated health
28	**Liao 2017**	Taiwan	Cross-sectional	NR	1,032	72.3±6.1	49.2	Mixed	RD;DA;SQ;ES;TS;AE;SC	Perc./Q	Self	LTW	IPAQ	Recr. walking ≥150 min/wk	aOR	Sex; age; work; edu.; marital; living; area; health; BMI

Study-level					Participants				Built-environment characteristics			LTPA / walking outcome			Analysis	
ID	Study (year)	Country	Design	Cohort / data source	N	Age (mean [SD])	Female (%)	Setting	BE domain(s)	BE assessment	Spatial scale	Outcome category	PA measure	Outcome definition	Effect	Adjusted covariates
29	Troped 2017	USA	Cross-sectional	NHS	2,732	72.8±6.8	100	Mixed	RD;LUM;DA;SC;SQ;AE;ES;TS	Obj.+Perc.	1200 m;Self	LTW	Q	Any; ≥150 min/wk LTW	aOR	Age; race; ethnicity; edu.; walking limits; BMI
30	Cerin 2020	Hong Kong, China	Cross-sectional	ALECS	909	76.5±6.0	66.3	Urban	RD;SC;DA;PG	Obj./GIS	800 m	LTW; NLTW	NWQ-CS	Within/outside-neigh. recr. walking	eβ	DAG-selected socio-demo.; health; SES covariates
31	He 2020	China	Cross-sectional	NR	1,161	71.22	53.6	Urban	RD;SC;LUM;DA	Obj./GIS	800 m	NLTW	Q	Leisure walk ≥150 vs <150 min/wk	aOR	Sex; age; edu.
32	Besser 2021	USA	Cross-sectional	NHTS 2017	73,523	65+	53.0	Mixed	RD	Obj./GIS	Admin.	NLTW	Travel diary	≥1 social/recreational neighborhood walk trip/day (purpose-stratified)	aOR	Age; sex; edu.; income; race; home/car; HH size; transit; health
33	Twardzik 2023	USA	Cross-sectional	NHATS 2018	4,836	68+	55.5	Mixed	DA	Obj./GIS	Admin.	LTW	Q	Any exercise walking last month	aOR	Age; sex; race; marital; edu.; homeownership; disability; metro; duration; disorder; cohesion
34	Kim 2025	Korea	Cross-sectional	Community survey	840	74.1±7.9	69.4	Urban	RD;LUM;DA;SC;SQ;AE;TS;ES	Perc./K-NEWS-A	Self	LTW	Q	≥30 min/day on ≥5 d/wk (≥150 min/wk)	aOR	Age; edu.; health/IADL/depression; social-network and neighborhood-environment variables

Notes:

(1) BE domains: TS = traffic safety; DA = destination accessibility; ES = environmental safety; AE = aesthetics; PG = parks and green spaces; RD = residential density; LUM = land-use mix; SC = street connectivity; SQ = sidewalk quality.

(2) Outcome categories: LTW = leisure-time walking; NLTW = neighbourhood leisure-time walking; General LTPA = general leisure-time physical activity. General LTPA combines leisure-time cycling, leisure-time walking and cycling combined, overall leisure-time physical activity, and leisure-time physical activity other than walking. Study-specific outcome wording and operational definitions are retained in the "Outcome definition" column.

(3) BE assessment: Obj. = objective; Perc. = perceived; GIS = geographic information system; NEWS = Neighborhood Environment Walkability Scale; K-NEWS-A = Korean version of the abbreviated NEWS; Q = questionnaire; Self = self-defined/perceived neighbourhood; Admin. = administrative spatial unit.

(4) PA measures: IPAQ = International Physical Activity Questionnaire; IPAQ-LF = IPAQ long form; CHAMPS = Community Healthy Activities Model Program for Seniors; PASE = Physical Activity Scale for the Elderly; SQUASH = Short Questionnaire to Assess Health-enhancing Physical Activity; NWQ-CS = Neighborhood Walking Questionnaire–Chinese version.

(5) Effect measures: OR = odds ratio; aOR = adjusted odds ratio; aPOR = adjusted prevalence odds ratio; eβ = exponentiated regression coefficient. NR = not reported; CS = cross-sectional; IADL = instrumental activities of daily living; SES = socioeconomic status; BMI = body mass index.

(6) Country/setting labels are abbreviated in the source extraction where appropriate. Cerin 2013a and Cerin 2013b are two distinct publications from the same Hong Kong sample. For Wang 2010, N = 114 refers to the survey sample; the objective GIS sub-sample comprised 61 participants. For Gómez 2010, N = 1,966 is the recruited study sample; regression analyses with complete environmental-perception data used 1,886 observations.

Supplementary Table S2. Operational definitions, measurement rules, and analytic handling of community built-environment domains, leisure-time physical activity outcomes, and effect-scale classifications.

Category	Full name / construct	Operational definition and eligible indicators	Measurement / operationalization observed in included studies	Direction / eligibility rule	Decision rules and analytic handling
**Panel A. Community built-environment domains**					
TS	**Traffic safety**	Risk or perceived risk posed by motorized traffic to walking or outdoor activity; includes traffic volume/speed, dangerous traffic, crossing safety, pedestrian signals, and road-crossing difficulty.	Perceived questionnaire/NEWS items and objective or audited traffic-related indicators.	Higher values indicating greater traffic danger were reverse-harmonized so that a more favorable condition corresponded to OR > 1.	Crime/violence/personal security were classified as ES rather than TS.
DA	**Destination accessibility**	Access to destinations, services, amenities, public transport, community facilities, and recreational opportunities; includes proximity, distance, counts, density, diversity, or perceived access.	Objective GIS-based distances/counts/densities and perceived access items.	Longer distance or poorer access was reverse-harmonized when necessary.	Indicators representing access to specific destinations were classified as DA rather than LUM.

Category	Full name / construct	Operational definition and eligible indicators	Measurement / operationalization observed in included studies	Direction / eligibility rule	Decision rules and analytic handling
ES	**Environmental safety**	Neighborhood safety from non-traffic threats, including crime, violence, personal security, fear of crime, and neighborhood security.	Perceived safety questionnaires/NEWS items and objective safety indicators when available.	Higher values representing greater danger or insecurity were reverse-harmonized.	Traffic-related danger was classified as TS.
AE	**Aesthetics**	Visual and experiential qualities that may make outdoor activity more appealing, including attractiveness, pleasant scenery, cleanliness, maintenance, visual interest, and streetscape quality.	Primarily perceived questionnaire/NEWS items; some objective/audit indicators.	Less favorable coding was reversed when necessary.	Measures primarily representing parks/green-space resources were classified as PG.
PG	**Parks and green spaces**	Availability, proximity, accessibility, quantity, or quality of parks, public open spaces, green spaces, gardens, and comparable recreational green areas.	GIS-based distance/availability measures and perceived access/quality measures.	Greater availability/access/quality was treated as the favorable direction; distance measures were inverted when necessary.	General aesthetics/greenery not explicitly representing a park or green-space resource was not automatically classified as PG.
RD	**Residential density**	Density of residences, dwellings, households, or population within the residential/neighborhood area.	Objective GIS/administrative density indicators and, less commonly, perceived density measures.	Direction was retained as reported unless original coding explicitly represented a less favorable contrast.	Density of destinations/amenities was classified according to the represented construct, most commonly DA.
LUM	**Land-use mix**	Degree of heterogeneity, diversity, or mixing of land uses, including entropy/mix indices and residential-commercial mix.	Objective GIS-based land-use mix indices and perceived mixed-use indicators.	Higher mix was treated as the favorable direction when supported by the original coding.	Single-destination proximity or counts were classified as DA.
SC	**Street connectivity**	Configuration and connectedness of the street/route network, including intersection density, route directness, block size/length, cul-de-sac prevalence, and connectivity indices.	Objective GIS/network measures and perceived connectivity items.	Measures coded as poorer connectivity were reversed when necessary.	Sidewalk/path condition or quality was classified as SQ.
SQ	**Sidewalk quality**	Presence, continuity, coverage, width, condition, maintenance, accessibility, or perceived quality of sidewalks, footpaths, pavements, and comparable pedestrian infrastructure.	Perceived questionnaire/NEWS items and objective/audit measures.	Poorer sidewalk condition/availability was reverse-harmonized when necessary.	Street-network configuration itself was classified as SC.
**Panel B. Leisure-time physical activity outcome categories**					
LTW	Leisure-time walking	Walking undertaken for leisure, recreation, exercise, or similar non-transport purposes without an explicit requirement that it occur within the residential neighborhood.	Questionnaire-based recreational/leisure/exercise walking measures, including instruments such as CHAMPS, IPAQ, Active Australia Survey, SQUASH, PASE, and study-specific questionnaires.	Purpose had to be leisure/recreation/exercise; no neighborhood-location restriction was required.	Analyzed separately. Current CAT/UNIT dataset: 227 effect sizes (138 CAT; 89 UNIT) from 22 independen study samples.
NLTW	Neighbourhood leisure-time walking	Leisure, recreational, or exercise walking explicitly reported as occurring within the participant's neighborhood, residential area, or comparable local community.	Neighborhood walking questionnaires (e.g., NWQ-CS), travel-diary or study-specific neighborhood walking measures.	Neighborhood location had to be explicit in the outcome definition, instrument, or analytic description.	Analyzed separately. Current CAT/UNIT dataset: 45 effect sizes (12 CAT; 33 UNIT) from 7 independent study samples.
General LTPA	General leisure-time physical activity	Other eligible leisure-time physical activity outcomes that did not meet LTW or NLTW definitions, including leisure-time cycling, combined leisure-time walking/cycling, overall/general LTPA, and eligible non-walking LTPA.	Study-specific questionnaires and validated PA instruments (e.g., IPAQ, SQUASH, PASE) when the full-text definition satisfied leisure-time PA eligibility.	Broad or mixed-purpose PA outcomes were retained only when full-text review confirmed that the measure represented eligible leisure-time physical activity.	Analyzed separately. Current CAT/UNIT dataset: 72 effect sizes (49 CAT; 23 UNIT) from 9 independent study samples.
**Panel C. Effect-scale classifications**					

Category	Full name / construct	Operational definition and eligible indicators	Measurement / operationalization observed in included studies	Direction / eligibility rule	Decision rules and analytic handling
**CAT**	Categorical comparison	Effect estimates based on an explicit categorical contrast with a clearly defined reference group, including presence vs absence, high vs low, better vs worse, ordered categories, or study-defined quantiles.	Adjusted OR/aOR/aPOR contrasts extracted from the most fully adjusted eligible model.	Reference and comparison categories were checked directly; unfavorable coding was inverted when necessary.	Primary quantitative effect scale. Current three outcome-specific datasets contain 199 CAT effect sizes in total.
**UNIT**	Continuous unit-based estimate	Continuous built-environment estimates for which the increment represented by the reported OR was explicitly stated or could be unambiguously derived (e.g., per unit, specified distance/density increment, SD, or IQR).	Continuous adjusted ORs with a recoverable increment; original scaling was retained.	Knowing the measurement unit alone was insufficient; the exact model increment represented by the OR had to be known.	Analyzed in separate three-level models. Current three outcome-specific datasets contain 145 UNIT effect sizes in total.

Note. TS, traffic safety; DA, destination accessibility; ES, environmental safety; AE, aesthetics; PG, parks and green spaces; RD, residential density; LUM, land-use mix; SC, street connectivity; SQ, sidewalk quality; LTW, leisure-time walking; NLTW, neighbourhood leisure-time walking; LTPA, leisure-time physical activity; CAT, categorical comparison; UNIT, continuous unit-based estimate; OR, odds ratio; SD, standard deviation; IQR, interquartile range. Only effect estimates meeting the prespecified CAT or UNIT definitions were included in the quantitative synthesis; CAT and UNIT were modeled separately because they represent different estimands.

Supplementary Table S3. JBI critical appraisal of included analytical cross-sectional studies.

No.	Study	Q1	Q2	Q3	Q4	Q5	Q6	Q7	Q8	Overall appraisal
1	Booth 2000	Yes	Yes	Unclear	Yes	Yes	Yes	Yes	Yes	Include
2	Berke 2007	Yes	Yes	Yes	Yes	Yes	Yes	Unclear	Yes	Include
3	Sugiyama 2008	Yes	Yes	Unclear	No	Yes	Yes	Unclear	Yes	Include
4	Salvador 2009	Yes	Yes	Yes	Yes	Yes	Yes	Yes	Yes	Include
5	Gomez 2010	Yes	Yes	Yes	Yes	Yes	Yes	Yes	Yes	Include
6	Parra 2010	Yes	Yes	Yes	Yes	Yes	Yes	Yes	Yes	Include
7	Satariano 2010	Yes	Yes	Yes	Yes	Yes	Yes	Yes	Yes	Include
8	Wang 2010	Unclear	Yes	Unclear	Yes	Yes	Yes	Unclear	Yes	Include
9	Hanibuchi 2011	Yes	Yes	Yes	Yes	Yes	Yes	Unclear	Yes	Include
10	Inoue 2011	Yes	Yes	Yes	Yes	Yes	Yes	Yes	Yes	Include
11	Boruff 2012	Unclear	Yes	Yes	Yes	Yes	Yes	Yes	Yes	Include
12	Nathan 2012	Yes	Yes	Yes	Yes	Yes	Yes	Yes	Yes	Include
13	Pelclová 2012	Yes	Yes	Yes	Yes	Yes	Yes	Yes	Yes	Include
14	Tsunoda 2012	Yes	Yes	Yes	Yes	Yes	Yes	Yes	Yes	Include
15	Van Cauwenberg 2012	Yes	Yes	Unclear	No	Yes	Yes	Unclear	Yes	Include
16	CerinA 2013	Yes	Yes	Yes	Yes	Yes	Yes	Yes	Yes	Include
17	CerinB 2013	Yes	Yes	Yes	Yes	Yes	Yes	Yes	Yes	Include
18	Kramer 2013	Yes	Yes	Yes	Yes	Yes	Yes	Yes	Yes	Include
19	Ding 2014	Yes	Yes	Yes	Yes	Yes	Yes	Yes	Yes	Include
20	Nathan 2014	Yes	Yes	Yes	Yes	Yes	Yes	Yes	Yes	Include
21	Troped 2014	Yes	Yes	Yes	Yes	Yes	Yes	Yes	Yes	Include
22	Gao 2015	Yes	Yes	Yes	Yes	Yes	Yes	Yes	Yes	Include
23	McMorris 2015	Yes	Yes	Yes	Yes	Yes	Yes	Yes	Yes	Include
24	Procter-Gray 2015	Yes	Yes	Yes	Yes	Yes	Yes	Unclear	Yes	Include
25	Barnett 2016	Yes	Yes	Yes	Yes	Yes	Yes	Yes	Yes	Include
26	Giehl 2016	Yes	Yes	Yes	Yes	Yes	Yes	Yes	Yes	Include
27	Corseuil Giehl 2017	Yes	Yes	Yes	Yes	Yes	Yes	Yes	Yes	Include
28	Liao 2017	Yes	Yes	Yes	Yes	Yes	Yes	Yes	Yes	Include
29	Troped 2017	Yes	Yes	Yes	Yes	Yes	Yes	Yes	Yes	Include
30	Cerin 2020	Yes	Yes	Yes	Yes	Yes	Yes	Yes	Yes	Include
31	He 2020	Yes	Yes	Yes	Yes	Yes	Yes	Unclear	Yes	Include
32	Besser 2021	Yes	Yes	Yes	Yes	Yes	Yes	Yes	Yes	Include
33	Twardzik 2023	Yes	Yes	Yes	Yes	Yes	Yes	Unclear	Yes	Include
34	Kim 2025	Yes	Yes	Yes	Yes	Yes	Yes	Yes	Yes	Include

Notes for Supplementary Table S3

Item	JBI criterion
**Q1**	Were the criteria for inclusion in the sample clearly defined?
**Q2**	Were the study subjects and the setting described in detail?
**Q3**	Was the exposure measured in a valid and reliable way?
**Q4**	Were objective, standard criteria used for measurement of the condition?
**Q5**	Were confounding factors identified?
**Q6**	Were strategies to deal with confounding factors stated?
**Q7**	Were the outcomes measured in a valid and reliable way?
**Q8**	Was appropriate statistical analysis used?

Appraisal judgments: Yes, No, Unclear, or Not applicable. Overall appraisal: Include, Exclude, or Seek further info. No numerical score or High/Moderate/Low quality classification was applied.

Appraisal tool: Joanna Briggs Institute (JBI) Critical Appraisal Checklist for Analytical Cross-Sectional Studies.

Supplementary Table S4. Main pooled effects, heterogeneity estimates, prediction intervals, CR2 robust inference, and model-structure comparisons for LTW, NLTW, and General LTPA.

Panel A. Main three-level random-effects models

Outcome	Scale	k (g)	Pooled OR (95% CI)	p	95% PI	Q (df), p	tau2 L3	tau2 L2	I2 total (%)	I2 L3 (%)	I2 L2 (%)	CR2 OR (95% CI)	CR2 p	CR2 df
LTW	CAT	138 (14)	1.103 (1.022-1.191)	0.012	0.788-1.545	300.46 (137), <0.001	0.014	0.0130	55.9	28.7	27.1	1.103 (1.015-1.200)	0.025	12.14
LTW	UNIT	89 (11)	1.068 (0.998-1.144)	0.058	0.856-1.333	316.60 (88), <0.001	0.011	0.0002	98.9	97.0	1.9	1.068 (0.989-1.154)	0.084	9.40
NLTW	CAT	12 (3)	2.008 (0.879-4.591)	0.090	0.352-11.448	56.46 (11), <0.001	0.358	0.1260	90.0	66.6	23.4	2.008 (0.385-10.464)	0.210	1.99
NLTW	UNIT	33 (5)	1.063 (1.017-1.112)	0.008	0.932-1.213	76.21 (32), <0.001	0.001	0.0030	92.8	18.1	74.8	1.063 (0.978-1.156)	0.094	2.36
General LTPA	CAT	49 (7)	1.180 (1.089-1.278)	<0.001	0.911-1.528	116.20 (48), <0.001	0.006	0.0090	57.4	24.0	33.3	1.180 (1.067-1.304)	0.007	5.62
General LTPA	UNIT	23 (4)	1.154 (0.871-1.528)	0.302	0.623-2.136	124.53 (22), <0.001	0.070	0.0000	100.0	100.0	0.0	1.154 (0.756-1.761)	0.358	2.97

Panel B. Comparison of three-level and reduced two-level model structures (ML estimation)

Outcome	Scale	Three-level AIC	Three-level BIC	Reduced AIC	Reduced BIC	LRT	p
LTW	CAT	25.93	34.71	37.97	43.83	14.046	<0.001
LTW	UNIT	-46.72	-39.25	-21.83	-16.86	26.885	<0.001
NLTW	CAT	28.89	30.35	37.33	38.30	10.438	0.001
NLTW	UNIT	-48.04	-43.55	-34.24	-31.24	15.805	<0.001
General LTPA	CAT	15.32	21.00	17.90	21.68	4.577	0.032
General LTPA	UNIT	-109.21	-105.80	-106.68	-104.41	4.525	0.033

Note. k, number of effect estimates; g, number of independent study samples; CI, confidence interval; PI, prediction interval; tau2 L3, between-study/sample variance; tau2 L2, within-study/sample variance; I2, proportion of variability attributable to heterogeneity; CR2, cluster-robust variance estimation with small-sample correction; ML, maximum likelihood; AIC, Akaike information criterion; BIC, Bayesian information criterion; LRT, likelihood-ratio test. The reduced model is the corresponding model without the Level-2 variance component used in the two-versus-three-level comparison. CAT is the primary effect scale and UNIT is a separate secondary quantitative analysis.

Supplementary Table S5. Subgroup analyses for LTW, NLTW, and General LTPA by built-environment domain and study characteristics.

Outcome	Scale	Moderator	Subgroup	k (g)	Standard OR (95% CI)	p	CR2 OR (95% CI)	CR2 p	Omnibus p Standard / CR2	Support
LTW	CAT	Built-environment domain	AE	6 (5)	1.504 (1.250-1.809)	<0.001	1.504 (1.013-2.233)	0.046	0.002 / NE	Relatively supported
LTW	CAT	Built-environment domain	DA	59 (9)	1.117 (1.004-1.242)	0.042	1.117 (0.960-1.298)	0.132	0.002 / NE	Relatively supported
LTW	CAT	Built-environment domain	ES	15 (8)	1.033 (0.896-1.192)	0.651	1.033 (0.835-1.279)	0.727	0.002 / NE	Relatively supported
LTW	CAT	Built-environment domain	LUM	3 (2)	1.035 (0.782-1.368)	0.810	1.035 (0.125-8.570)	0.898	0.002 / NE	Sparse
LTW	CAT	Built-environment domain	PG	6 (3)	1.295 (1.075-1.560)	0.007	1.295 (0.937-1.791)	0.076	0.002 / NE	Limited
LTW	CAT	Built-environment domain	RD	7 (5)	0.957 (0.790-1.159)	0.651	0.957 (0.781-1.172)	0.570	0.002 / NE	Relatively supported
LTW	CAT	Built-environment domain	SC	15 (6)	1.030 (0.892-1.189)	0.683	1.030 (0.793-1.337)	0.771	0.002 / NE	Relatively supported
LTW	CAT	Built-environment domain	SQ	19 (8)	1.057 (0.928-1.203)	0.402	1.057 (0.945-1.181)	0.282	0.002 / NE	Relatively supported
LTW	CAT	Built-environment domain	TS	8 (5)	1.238 (1.062-1.444)	0.007	1.238 (0.892-1.718)	0.154	0.002 / NE	Relatively supported
LTW	CAT	Environment-measure type	Objective	70 (8)	1.071 (0.965-1.188)	0.196	1.071 (0.916-1.251)	0.325	0.369 / 0.506	Relatively supported
LTW	CAT	Environment-measure type	Perceived	68 (8)	1.136 (1.025-1.259)	0.015	1.136 (1.005-1.284)	0.043	0.369 / 0.506	Relatively supported
LTW	CAT	Region	North America	26 (5)	1.114 (0.944-1.315)	0.200	1.114 (0.809-1.533)	0.394	0.899 / 0.469	Relatively supported
LTW	CAT	Region	Asia	36 (3)	1.132 (0.942-1.361)	0.184	1.132 (0.892-1.436)	0.154	0.899 / 0.469	Limited
LTW	CAT	Region	Europe	7 (1)	1.030 (0.701-1.514)	0.879	NE	NE	0.899 / 0.469	Descriptive
LTW	CAT	Region	Oceania	24 (1)	0.963 (0.712-1.302)	0.804	NE	NE	0.899 / 0.469	Descriptive
LTW	CAT	Region	South America	45 (4)	1.125 (0.956-1.324)	0.155	1.125 (0.913-1.387)	0.170	0.899 / 0.469	Limited
LTW	CAT	Sex composition	Mixed	123 (12)	1.081 (0.995-1.175)	0.065	1.081 (0.984-1.188)	0.095	0.029 / 0.306	Relatively supported
LTW	CAT	Sex composition	Female	9 (2)	1.322 (1.078-1.621)	0.008	1.322 (0.441-3.963)	0.192	0.029 / 0.306	Sparse
LTW	CAT	Sex composition	Male	6 (1)	0.932 (0.684-1.269)	0.651	NE	NE	0.029 / 0.306	Descriptive
LTW	CAT	Spatial scale	100 m	4 (1)	1.120 (0.811-1.546)	0.488	NE	NE	0.964 / NE	Descriptive
LTW	CAT	Spatial scale	1000 m	4 (1)	1.184 (0.848-1.653)	0.318	NE	NE	0.964 / NE	Descriptive
LTW	CAT	Spatial scale	1200 m	3 (1)	1.229 (0.896-1.686)	0.197	NE	NE	0.964 / NE	Descriptive
LTW	CAT	Spatial scale	400 m	12 (1)	0.958 (0.709-1.294)	0.777	NE	NE	0.964 / NE	Descriptive
LTW	CAT	Spatial scale	500 m	18 (3)	1.062 (0.862-1.308)	0.568	1.062 (0.601-1.876)	0.677	0.964 / NE	Limited
LTW	CAT	Spatial scale	800 m	12 (1)	0.966 (0.718-1.300)	0.819	NE	NE	0.964 / NE	Descriptive
LTW	CAT	Spatial scale	Administrative	17 (3)	1.112 (0.895-1.382)	0.335	1.112 (0.659-1.877)	0.375	0.964 / NE	Limited
LTW	CAT	Spatial scale	Local area (NEWS; no metric buffer reported)	12 (1)	1.167 (0.851-1.600)	0.334	NE	NE	0.964 / NE	Descriptive
LTW	CAT	Spatial scale	Neighborhood within 10–15 min walk	8 (1)	1.137 (0.838-1.543)	0.404	NE	NE	0.964 / NE	Descriptive
LTW	CAT	Spatial scale	Self-defined	6 (1)	1.313 (0.937-1.839)	0.112	NE	NE	0.964 / NE	Descriptive
LTW	CAT	Urbanicity	Urban	88 (7)	1.094 (1.002-1.195)	0.045	1.094 (0.965-1.240)	0.127	0.385 / 0.122	Relatively supported
LTW	CAT	Urbanicity	Mixed	20 (3)	1.204 (1.049-1.381)	0.009	1.204 (1.019-1.421)	0.041	0.385 / 0.122	Limited
LTW	CAT	Urbanicity	Rural	18 (1)	1.026 (0.821-1.283)	0.819	NE	NE	0.385 / 0.122	Descriptive
LTW	UNIT	Built-environment domain	AE	13 (6)	1.161 (1.070-1.259)	<0.001	1.161 (0.946-1.426)	0.126	0.001 / NE	Relatively supported
LTW	UNIT	Built-environment domain	DA	18 (4)	1.102 (1.020-1.190)	0.014	1.102 (1.012-1.200)	0.031	0.001 / NE	Limited
LTW	UNIT	Built-environment domain	ES	9 (4)	0.833 (0.723-0.961)	0.013	0.833 (0.735-0.944)	0.020	0.001 / NE	Limited
LTW	UNIT	Built-environment domain	LUM	11 (5)	1.052 (0.968-1.144)	0.225	1.052 (0.982-1.128)	0.118	0.001 / NE	Relatively supported
LTW	UNIT	Built-environment domain	PG	7 (1)	1.029 (0.841-1.258)	0.781	NE	NE	0.001 / NE	Descriptive
LTW	UNIT	Built-environment domain	RD	5 (3)	1.071 (0.980-1.171)	0.130	1.071 (0.989-1.159)	0.073	0.001 / NE	Limited
LTW	UNIT	Built-environment domain	SC	7 (2)	1.131 (0.980-1.304)	0.091	1.131 (0.867-1.474)	0.144	0.001 / NE	Sparse
LTW	UNIT	Built-environment domain	SQ	10 (4)	1.166 (1.067-1.274)	0.001	1.166 (0.992-1.371)	0.058	0.001 / NE	Limited
LTW	UNIT	Built-environment domain	TS	9 (3)	1.052 (0.918-1.205)	0.462	1.052 (0.632-1.751)	0.712	0.001 / NE	Limited

Outcome	Scale	Moderator	Subgroup	k (g)	Standard OR (95% CI)	p	CR2 OR (95% CI)	CR2 p	Omnibus p Standard / CR2	Support
LTW	UNIT	Environment-measure type	Objective	30 (6)	1.027 (0.962-1.096)	0.420	1.027 (0.984-1.072)	0.178	0.016 / 0.075	Relatively supported
LTW	UNIT	Environment-measure type	Perceived	59 (6)	1.132 (1.051-1.220)	0.001	1.132 (0.996-1.287)	0.055	0.016 / 0.075	Relatively supported
LTW	UNIT	Region	North America	38 (3)	1.165 (1.045-1.300)	0.007	1.165 (0.860-1.579)	0.163	0.229 / 0.655	Limited
LTW	UNIT	Region	Asia	29 (3)	1.019 (0.914-1.135)	0.733	1.019 (0.999-1.039)	0.054	0.229 / 0.655	Limited
LTW	UNIT	Region	Europe	4 (2)	1.115 (0.908-1.371)	0.295	1.115 (0.039-31.686)	0.750	0.229 / 0.655	Sparse
LTW	UNIT	Region	Oceania	18 (3)	1.010 (0.901-1.131)	0.867	1.010 (0.948-1.075)	0.562	0.229 / 0.655	Limited
LTW	UNIT	Sex composition	Mixed	51 (7)	1.062 (0.977-1.154)	0.155	1.062 (0.980-1.151)	0.116	0.213 / 0.843	Relatively supported
LTW	UNIT	Sex composition	Female	26 (4)	1.099 (0.978-1.234)	0.112	1.099 (0.816-1.479)	0.361	0.213 / 0.843	Limited
LTW	UNIT	Sex composition	Male	12 (2)	0.963 (0.810-1.145)	0.667	0.963 (0.423-2.191)	0.823	0.213 / 0.843	Sparse
LTW	UNIT	Spatial scale	1000 m	7 (1)	1.027 (0.989-1.068)	0.158	NE	NE	0.305 / NE	Descriptive
LTW	UNIT	Spatial scale	1200 m	10 (1)	1.043 (1.004-1.084)	0.030	NE	NE	0.305 / NE	Descriptive
LTW	UNIT	Spatial scale	400 m	7 (2)	1.008 (0.978-1.040)	0.586	1.008 (0.905-1.123)	0.530	0.305 / NE	Sparse
LTW	UNIT	Spatial scale	500 m	2 (1)	1.090 (1.003-1.184)	0.043	NE	NE	0.305 / NE	Descriptive
LTW	UNIT	Spatial scale	800 m	4 (2)	1.013 (0.982-1.045)	0.400	1.013 (0.850-1.207)	0.544	0.305 / NE	Sparse
LTW	UNIT	Spatial scale	Administrative	2 (1)	0.828 (0.638-1.075)	0.150	NE	NE	0.305 / NE	Descriptive
LTW	UNIT	Spatial scale	Self-defined	4 (1)	0.998 (0.833-1.196)	0.981	NE	NE	0.305 / NE	Descriptive
LTW	UNIT	Urbanicity	Urban	11 (3)	1.008 (0.906-1.121)	0.883	1.008 (0.965-1.053)	0.524	0.560 / 0.668	Limited
LTW	UNIT	Urbanicity	Mixed	26 (3)	1.061 (0.920-1.225)	0.404	1.061 (0.612-1.842)	0.650	0.560 / 0.668	Limited
NLTW	CAT	Built-environment domain	DA	3 (2)	1.727 (0.505-5.907)	0.328	1.727 (0.026-114.045)	0.437	0.594 / NE	Sparse
NLTW	CAT	Built-environment domain	ES	2 (1)	4.669 (0.753-28.942)	0.086	NE	NE	0.594 / NE	Descriptive
NLTW	CAT	Built-environment domain	LUM	1 (1)	2.447 (0.552-10.856)	0.198	NE	NE	0.594 / NE	Descriptive
NLTW	CAT	Built-environment domain	RD	5 (2)	1.624 (0.510-5.167)	0.355	1.624 (0.061-43.394)	0.372	0.594 / NE	Sparse
NLTW	CAT	Built-environment domain	SC	1 (1)	2.746 (0.615-12.264)	0.155	NE	NE	0.594 / NE	Descriptive
NLTW	CAT	Environment-measure type	Objective	8 (2)	1.497 (0.692-3.242)	0.271	1.497 (0.016-138.423)	0.461	0.165 / 0.201	Sparse
NLTW	CAT	Environment-measure type	Perceived	4 (1)	4.032 (1.148-14.166)	0.033	NE	NE	0.165 / 0.201	Descriptive
NLTW	CAT	Region	North America	8 (2)	2.767 (1.373-5.575)	0.009	2.767 (0.054-140.773)	0.188	0.090 / 0.198	Sparse
NLTW	CAT	Region	Asia	4 (1)	1.062 (0.433-2.604)	0.884	NE	NE	0.090 / 0.198	Descriptive
NLTW	CAT	Spatial scale	800 m	3 (1)	1.724 (0.448-6.630)	0.361	NE	NE	0.657 / 0.414	Descriptive
NLTW	CAT	Spatial scale	Administrative	5 (2)	1.387 (0.408-4.721)	0.537	1.387 (0.003-714.855)	0.626	0.657 / 0.414	Sparse
NLTW	CAT	Urbanicity	Urban	8 (2)	2.009 (0.455-8.880)	0.320	2.009 (0.000-13137.797)	0.498	0.961 / 0.969	Sparse
NLTW	CAT	Urbanicity	Mixed	4 (1)	2.128 (0.271-16.724)	0.433	NE	NE	0.961 / 0.969	Descriptive
NLTW	UNIT	Built-environment domain	AE	1 (1)	1.069 (0.895-1.277)	0.445	NE	NE	<0.001 / NE	Descriptive
NLTW	UNIT	Built-environment domain	DA	12 (2)	1.056 (1.019-1.095)	0.004	1.056 (0.746-1.495)	0.297	<0.001 / NE	Sparse
NLTW	UNIT	Built-environment domain	ES	4 (2)	1.178 (1.089-1.273)	<0.001	1.178 (0.989-1.403)	0.054	<0.001 / NE	Sparse
NLTW	UNIT	Built-environment domain	LUM	1 (1)	1.062 (0.869-1.298)	0.541	NE	NE	<0.001 / NE	Descriptive
NLTW	UNIT	Built-environment domain	PG	1 (1)	1.220 (0.847-1.757)	0.273	NE	NE	<0.001 / NE	Descriptive
NLTW	UNIT	Built-environment domain	RD	4 (2)	1.001 (1.000-1.003)	0.174	1.001 (1.000-1.002)	0.027	<0.001 / NE	Sparse
NLTW	UNIT	Built-environment domain	SC	2 (1)	1.053 (0.926-1.197)	0.418	NE	NE	<0.001 / NE	Descriptive
NLTW	UNIT	Built-environment domain	SQ	5 (2)	1.151 (1.048-1.264)	0.005	1.151 (0.404-3.277)	0.340	<0.001 / NE	Sparse
NLTW	UNIT	Built-environment domain	TS	3 (1)	0.989 (0.903-1.083)	0.799	NE	NE	<0.001 / NE	Descriptive
NLTW	UNIT	Environment-measure type	Objective	12 (3)	1.041 (0.992-1.092)	0.100	1.041 (0.923-1.173)	0.208	0.145 / 0.139	Limited

Outcome	Scale	Moderator	Subgroup	k (g)	Standard OR (95% CI)	p	CR2 OR (95% CI)	CR2 p	Omnibus p Standard / CR2	Support
NLTW	UNIT	Environment-measure type	Perceived	21 (2)	1.093 (1.043-1.145)	0.001	1.093 (0.925-1.291)	0.093	0.145 / 0.139	Sparse
NLTW	UNIT	Region	North America	10 (2)	1.061 (0.943-1.194)	0.311	1.061 (0.268-4.208)	0.685	0.902 / 0.948	Sparse
NLTW	UNIT	Region	Asia	23 (3)	1.070 (0.991-1.156)	0.082	1.070 (0.895-1.281)	0.217	0.902 / 0.948	Limited
NLTW	UNIT	Spatial scale	400 m	1 (1)	1.165 (0.964-1.407)	0.109	NE	NE	0.597 / NE	Descriptive
NLTW	UNIT	Spatial scale	800 m	2 (1)	1.003 (0.870-1.156)	0.966	NE	NE	0.597 / NE	Descriptive
NLTW	UNIT	Spatial scale	Road-network distance	9 (1)	1.042 (0.913-1.189)	0.530	NE	NE	0.597 / NE	Descriptive
NLTW	UNIT	Spatial scale	Self-defined neighbourhood (≤15-min walk)	20 (1)	1.087 (0.958-1.234)	0.187	NE	NE	0.597 / NE	Descriptive
General LTPA	CAT	Built-environment domain	AE	8 (2)	1.254 (0.943-1.666)	0.116	1.254 (0.667-2.358)	0.360	<0.001 / NE	Sparse
General LTPA	CAT	Built-environment domain	DA	15 (3)	1.218 (0.951-1.559)	0.115	1.218 (0.851-1.743)	0.215	<0.001 / NE	Limited
General LTPA	CAT	Built-environment domain	ES	2 (1)	0.911 (0.590-1.407)	0.669	NE	NE	<0.001 / NE	Descriptive
General LTPA	CAT	Built-environment domain	LUM	2 (1)	0.857 (0.540-1.361)	0.503	NE	NE	<0.001 / NE	Descriptive
General LTPA	CAT	Built-environment domain	PG	9 (4)	1.424 (1.113-1.821)	0.006	1.424 (1.036-1.956)	0.035	<0.001 / NE	Limited
General LTPA	CAT	Built-environment domain	RD	4 (2)	1.046 (0.675-1.622)	0.836	1.046 (0.541-2.024)	0.613	<0.001 / NE	Sparse
General LTPA	CAT	Built-environment domain	SC	2 (1)	0.405 (0.255-0.642)	<0.001	NE	NE	<0.001 / NE	Descriptive
General LTPA	CAT	Built-environment domain	SQ	5 (2)	1.362 (0.976-1.901)	0.068	1.362 (0.631-2.938)	0.250	<0.001 / NE	Sparse
General LTPA	CAT	Built-environment domain	TS	2 (1)	0.944 (0.621-1.436)	0.784	NE	NE	<0.001 / NE	Descriptive
General LTPA	CAT	Environment-measure type	Objective	17 (4)	1.094 (1.029-1.162)	0.005	1.094 (0.991-1.207)	0.060	0.002 / 0.021	Limited
General LTPA	CAT	Environment-measure type	Perceived	32 (3)	1.277 (1.188-1.373)	<0.001	1.277 (1.086-1.501)	0.026	0.002 / 0.021	Limited
General LTPA	CAT	Region	North America	3 (1)	1.060 (0.826-1.360)	0.639	NE	NE	0.736 / 0.700	Descriptive
General LTPA	CAT	Region	Asia	36 (3)	1.218 (1.067-1.390)	0.004	1.218 (0.918-1.616)	0.095	0.736 / 0.700	Limited
General LTPA	CAT	Region	Europe	2 (1)	1.079 (0.814-1.429)	0.590	NE	NE	0.736 / 0.700	Descriptive
General LTPA	CAT	Region	Oceania	2 (1)	1.220 (0.899-1.655)	0.196	NE	NE	0.736 / 0.700	Descriptive
General LTPA	CAT	Region	South America	6 (1)	1.312 (0.988-1.743)	0.060	NE	NE	0.736 / 0.700	Descriptive
General LTPA	CAT	Spatial scale	1000 m	2 (1)	1.081 (0.814-1.436)	0.576	NE	NE	0.881 / 0.598	Descriptive
General LTPA	CAT	Spatial scale	250 m	2 (1)	1.108 (0.826-1.487)	0.480	NE	NE	0.881 / 0.598	Descriptive
General LTPA	CAT	Spatial scale	500 m	11 (3)	1.151 (0.990-1.339)	0.065	1.151 (0.815-1.627)	0.218	0.881 / 0.598	Limited
General LTPA	CAT	Spatial scale	Administrative	14 (2)	1.211 (1.039-1.411)	0.017	1.211 (0.502-2.918)	0.223	0.881 / 0.598	Sparse
General LTPA	CAT	Urbanicity	Urban	21 (3)	1.192 (1.074-1.322)	0.001	1.192 (0.881-1.611)	0.122	0.098 / 0.024	Limited
General LTPA	CAT	Urbanicity	Mixed	8 (2)	1.079 (0.957-1.217)	0.210	1.079 (1.056-1.102)	0.014	0.098 / 0.024	Sparse
General LTPA	CAT	Urbanicity	Rural	18 (1)	1.347 (1.142-1.588)	0.001	NE	NE	0.098 / 0.024	Descriptive
General LTPA	UNIT	Built-environment domain	AE	1 (1)	1.212 (0.828-1.774)	0.299	NE	NE	0.009 / NE	Descriptive
General LTPA	UNIT	Built-environment domain	DA	4 (2)	1.204 (0.823-1.762)	0.315	1.204 (0.583-2.485)	0.383	0.009 / NE	Sparse
General LTPA	UNIT	Built-environment domain	ES	4 (3)	1.247 (0.852-1.826)	0.236	1.247 (0.567-2.745)	0.350	0.009 / NE	Limited
General LTPA	UNIT	Built-environment domain	LUM	3 (1)	1.001 (0.527-1.902)	0.998	NE	NE	0.009 / NE	Descriptive
General LTPA	UNIT	Built-environment domain	PG	1 (1)	1.237 (0.845-1.812)	0.253	NE	NE	0.009 / NE	Descriptive
General LTPA	UNIT	Built-environment domain	RD	3 (1)	1.004 (0.528-1.908)	0.989	NE	NE	0.009 / NE	Descriptive
General LTPA	UNIT	Built-environment domain	SC	6 (1)	1.001 (0.527-1.902)	0.997	NE	NE	0.009 / NE	Descriptive
General LTPA	UNIT	Built-environment domain	TS	1 (1)	1.223 (0.834-1.793)	0.279	NE	NE	0.009 / NE	Descriptive
General LTPA	UNIT	Environment-measure type	Objective	19 (2)	1.010 (0.673-1.516)	0.961	1.010 (0.796-1.281)	0.695	0.328 / 0.438	Sparse
General LTPA	UNIT	Environment-measure type	Perceived	4 (2)	1.341 (0.876-2.051)	0.167	1.341 (0.032-56.167)	0.501	0.328 / 0.438	Sparse
General LTPA	UNIT	Region	Asia	19 (2)	1.010 (0.673-1.516)	0.961	1.010 (0.796-1.281)	0.695	0.328 / 0.438	Sparse

Outcome	Scale	Moderator	Subgroup	k (g)	Standard OR (95% CI)	p	CR2 OR (95% CI)	CR2 p	Omnibus p Standard / CR2	Support
General LTPA	UNIT	Region	Europe	4 (2)	1.341 (0.876-2.051)	0.167	1.341 (0.032-56.167)	0.501	0.328 / 0.438	Sparse
General LTPA	UNIT	Sex composition	Mixed	21 (3)	1.014 (1.000-1.028)	0.049	1.014 (0.984-1.045)	0.186	<0.001 / NE	Limited
General LTPA	UNIT	Sex composition	Female	1 (1)	2.230 (1.536-3.238)	<0.001	NE	NE	<0.001 / NE	Descriptive
General LTPA	UNIT	Sex composition	Male	1 (1)	1.530 (1.039-2.253)	0.033	NE	NE	<0.001 / NE	Descriptive
General LTPA	UNIT	Spatial scale	1000 m	4 (1)	1.001 (0.895-1.120)	0.978	NE	NE	0.010 / NE	Descriptive
General LTPA	UNIT	Spatial scale	250 m	4 (1)	1.003 (0.897-1.122)	0.954	NE	NE	0.010 / NE	Descriptive
General LTPA	UNIT	Spatial scale	400 m	7 (1)	1.018 (0.909-1.139)	0.747	NE	NE	0.010 / NE	Descriptive
General LTPA	UNIT	Spatial scale	500 m	4 (1)	1.002 (0.896-1.122)	0.963	NE	NE	0.010 / NE	Descriptive
General LTPA	UNIT	Spatial scale	Administrative	2 (1)	1.860 (1.385-2.497)	<0.001	NE	NE	0.010 / NE	Descriptive
General LTPA	UNIT	Urbanicity	Urban	7 (1)	1.018 (0.517-2.003)	0.958	NE	NE	0.641 / 0.899	Descriptive
General LTPA	UNIT	Urbanicity	Mixed	16 (3)	1.217 (0.816-1.816)	0.319	1.217 (0.537-2.761)	0.409	0.641 / 0.899	Limited

Note. Omnibus p values are from moderator tests comparing all levels of the corresponding categorical moderator. CR2 omnibus tests are reported as NE when the Satterthwaite-adjusted denominator degrees of freedom were insufficient for stable estimation. For subgroups represented by a single independent study/sample (g = 1), CR2 subgroup confidence intervals and p values were not interpreted and are reported as NE; the standard model estimate is shown descriptively only, and the support category is labeled “Descriptive.” Support categories for the remaining subgroups are those generated in the prespecified subgroup feasibility checks. Subgroup analyses were exploratory, particularly where g was small.

Supplementary Table S6. Meta-regression analyses of female percentage, mean age, and publication year for LTW, NLTW, and General LTPA.

Outcome	Scale	Moderator	k (g)	beta (95% CI)	p	CR2 beta (95% CI)	CR2 p	CR2 df	Pseudo-R2 total (%)
LTW	CAT	Female percentage (%)	133 (13)	0.0030 (0.0000 to 0.0050)	0.023	0.0030 (-0.0040 to 0.0100)	0.155	1.49	6.6
LTW	CAT	Mean age (years)	119 (11)	0.0090 (-0.0160 to 0.0340)	0.476	0.0090 (-0.0360 to 0.0540)	0.529	2.35	-5.6
LTW	CAT	Publication year	138 (14)	0.0170 (-0.0030 to 0.0360)	0.094	0.0170 (-0.0170 to 0.0500)	0.232	3.63	8.8
LTW	UNIT	Female percentage (%)	84 (10)	0.0000 (-0.0010 to 0.0020)	0.561	0.0000 (-0.0060 to 0.0070)	0.738	1.51	4.7
LTW	UNIT	Mean age (years)	82 (9)	-0.0380 (-0.0650 to -0.0110)	0.007	-0.0380 (-0.1610 to 0.0860)	0.311	1.93	-63.9
LTW	UNIT	Publication year	89 (11)	-0.0040 (-0.0210 to 0.0140)	0.670	-0.0040 (-0.0310 to 0.0240)	0.661	2.36	-17.3
NLTW	CAT	Female percentage (%)	8 (2)	0.0220 (-0.0330 to 0.0780)	0.362	0.0220 (-0.0290 to 0.0740)	0.171	1.62	-1.9
NLTW	CAT	Mean age (years)	8 (2)	0.1010 (-0.0080 to 0.2110)	0.065	0.1010 (-0.0670 to 0.2700)	0.089	1.14	79.7
NLTW	CAT	Publication year	12 (3)	-0.0880 (-0.2430 to 0.0670)	0.235	-0.0880 (-1.1610 to 0.9850)	0.513	1.04	19.1
NLTW	UNIT	Female percentage (%)	3 (2)	0.0460 (-0.2390 to 0.3310)	0.288	0.0460 (0.0410 to 0.0510)	0.005	1.00	99.5
NLTW	UNIT	Mean age (years)	32 (4)	0.0450 (-0.0280 to 0.1190)	0.217	0.0450 (-0.2530 to 0.3440)	0.472	1.46	-173.6
NLTW	UNIT	Publication year	33 (5)	-0.0180 (-0.0370 to 0.0020)	0.076	-0.0180 (-0.0580 to 0.0230)	0.169	1.58	15.8
General LTPA	CAT	Female percentage (%)	46 (6)	0.0090 (-0.0170 to 0.0340)	0.483	0.0090 (-0.0370 to 0.0550)	0.507	2.13	-4.2
General LTPA	CAT	Mean age (years)	26 (3)	-0.0310 (-0.1520 to 0.0910)	0.608	-0.0310 (-0.5850 to 0.5240)	0.612	1.00	-5.9
General LTPA	CAT	Publication year	49 (7)	-0.0030 (-0.0240 to 0.0180)	0.776	-0.0030 (-0.0330 to 0.0270)	0.631	1.49	-21.7
General LTPA	UNIT	Female percentage (%)	16 (3)	0.0040 (-0.0020 to 0.0090)	0.170	0.0040 (0.0020 to 0.0050)	0.022	1.00	-2.8
General LTPA	UNIT	Publication year	23 (4)	0.1550 (-0.2140 to 0.5250)	0.393	0.1550 (-0.5540 to 0.8640)	0.430	1.90	-19.9

Note. Regression coefficients (beta) are expressed on the log-OR scale and represent the estimated change in log OR per one-unit increase in the moderator. Pseudo-R2 total is the proportional reduction in total estimated heterogeneity relative to the corresponding intercept-only model; negative values can occur because variance-component estimates may increase after adding a moderator. Analyses with insufficient data were not fitted and therefore do not appear in the table.

Supplementary Table S7. Publication-bias and small-study-effect diagnostics for LTW, NLTW, and General LTPA.

Outcome	Scale	k (g)	SE-Egger beta	p	CR2 p	Variance-Egger beta	p	Trim-and-fill k0	Adjusted OR (95% CI)
LTW	CAT	138 (14)	0.043	0.896	0.956	0.510	0.247	0	1.072 (1.030-1.115)
LTW	UNIT	89 (11)	0.397	0.074	0.629	-1.028	0.099	6	1.084 (1.048-1.122)
NLTW	CAT	12 (3)	3.428	0.010	0.107	2.940	0.019	2	1.623 (1.067-2.467)
NLTW	UNIT	33 (5)	1.065	<0.001	0.068	6.412	0.002	13	1.012 (0.971-1.055)
General LTPA	CAT	49 (7)	1.253	<0.001	0.005	3.470	<0.001	13	1.100 (1.009-1.199)
General LTPA	UNIT	23 (4)	2.404	<0.001	0.055	18.699	<0.001	4	1.008 (1.003-1.014)

Note. SE-Egger refers to the three-level Egger-type meta-regression using the effect-size standard error as moderator; CR2 p is the cluster-robust small-sample-corrected p value for the same slope. Variance-Egger uses sampling variance as an alternative moderator. Trim-and-fill was implemented as a conventional two-level random-effects sensitivity diagnostic and does not explicitly model the within-study/sample dependence structure; adjusted estimates are therefore interpreted as exploratory.

Supplementary Table S8. Comparison of original and outlier-removed pooled effects for LTW, NLTW, and General LTPA.

Outcome	Scale	Outliers n	Original k (g)	Original OR (95% CI)	p	Original I2 (%)	After removal k (g)	After removal OR (95% CI)	p	After removal I2 (%)	After-removal CR2 OR (95% CI); p
LTW	CAT	9	138 (14)	1.103 (1.022-1.191)	0.012	55.9	129 (14)	1.116 (1.046-1.189)	<0.001	44.6	1.116 (1.039-1.198); 0.006
LTW	UNIT	8	89 (11)	1.068 (0.998-1.144)	0.058	98.9	81 (11)	1.059 (1.015-1.105)	0.008	97.0	1.059 (1.010-1.111); 0.024
NLTW	CAT	0	12 (3)	2.008 (0.879-4.591)	0.090	90.0	12 (3)	2.008 (0.879-4.591)	0.090	90.0	2.008 (0.385-10.464); 0.210
NLTW	UNIT	1	33 (5)	1.063 (1.017-1.112)	0.008	92.8	32 (5)	1.057 (1.017-1.097)	0.006	90.9	1.057 (0.983-1.136); 0.086
General LTPA	CAT	4	49 (7)	1.180 (1.089-1.278)	<0.001	57.4	45 (7)	1.148 (1.072-1.231)	<0.001	41.1	1.148 (1.053-1.252); 0.009
General LTPA	UNIT	1	23 (4)	1.154 (0.871-1.528)	0.302	100.0	22 (4)	1.014 (1.001-1.028)	0.042	98.3	1.014 (0.984-1.045); 0.176

Note. Potential outliers were defined a priori as effect estimates with |studentized residual| > 1.96. When no outlier was identified, the outlier-removed model is identical to the original model. CR2 results shown are for the model after exclusion of flagged outliers.

Supplementary Table S9. Leave-one-study/sample-out sensitivity analyses for LTW, NLTW, and General LTPA.

Outcome	Scale	Independent study/sample removed	Remaining k	Pooled OR (95% CI)	p	Total tau2
LTW	CAT	Berke 2007	126	1.092 (1.008-1.183)	0.032	0.027
LTW	CAT	Gomez 2010	124	1.119 (1.035-1.210)	0.005	0.024
LTW	CAT	Satariano 2010	133	1.125 (1.054-1.201)	<0.001	0.021
LTW	CAT	Salvador 2009	126	1.098 (1.010-1.194)	0.028	0.027
LTW	CAT	Inoue 2011	130	1.100 (1.010-1.198)	0.028	0.030
LTW	CAT	Nathan 2012	114	1.119 (1.035-1.209)	0.005	0.034
LTW	CAT	Pelclova 2012	131	1.107 (1.021-1.201)	0.014	0.029
LTW	CAT	Tsunoda 2012	120	1.112 (1.023-1.208)	0.013	0.026
LTW	CAT	Ding 2014	134	1.100 (1.015-1.192)	0.020	0.029
LTW	CAT	Troped 2014	135	1.094 (1.008-1.186)	0.031	0.030
LTW	CAT	Giehl 2016	125	1.101 (1.011-1.199)	0.027	0.032
LTW	CAT	Corseuil Giehl 2017	132	1.090 (1.009-1.178)	0.030	0.027
LTW	CAT	Liao 2017	128	1.092 (1.007-1.183)	0.033	0.027
LTW	CAT	Twardzik 2023	136	1.094 (1.010-1.185)	0.027	0.029
LTW	UNIT	Sugiyama 2008	87	1.052 (0.988-1.121)	0.111	0.009
LTW	UNIT	Boruff 2012	82	1.075 (0.994-1.162)	0.070	0.013
LTW	UNIT	Nathan 2012	85	1.078 (1.000-1.162)	0.049	0.012
LTW	UNIT	CerinB 2013	84	1.077 (0.997-1.163)	0.060	0.013
LTW	UNIT	Kramer 2013	87	1.080 (1.010-1.155)	0.025	0.010
LTW	UNIT	Ding 2014	65	1.060 (0.983-1.144)	0.129	0.012
LTW	UNIT	Nathan 2014	82	1.073 (0.995-1.156)	0.066	0.013
LTW	UNIT	Troped 2014	79	1.070 (0.989-1.157)	0.089	0.013
LTW	UNIT	Troped 2017	85	1.037 (1.003-1.072)	0.034	0.002
LTW	UNIT	Cerin 2020	87	1.073 (0.993-1.160)	0.074	0.013
LTW	UNIT	Kim 2025	67	1.074 (0.995-1.160)	0.067	0.013
NLTW	CAT	Wang 2010	8	1.501 (0.656-3.433)	0.284	0.339
NLTW	CAT	He 2020	8	2.801 (1.335-5.877)	0.013	0.287
NLTW	CAT	Besser 2021	8	1.997 (0.423-9.420)	0.327	0.869
NLTW	UNIT	Wang 2010	32	1.060 (1.014-1.108)	0.011	0.004
NLTW	UNIT	Cerin 2013a	32	1.053 (1.007-1.101)	0.026	0.003
NLTW	UNIT	Procter-Gray 2015	24	1.083 (1.009-1.161)	0.028	0.007
NLTW	UNIT	Barnett 2016	13	1.068 (0.973-1.174)	0.150	0.005
NLTW	UNIT	Cerin 2020	31	1.079 (1.035-1.125)	<0.001	0.004
General LTPA	CAT	Booth 2000	47	1.179 (1.077-1.290)	<0.001	0.017
General LTPA	CAT	Parra 2010	43	1.154 (1.065-1.252)	<0.001	0.009
General LTPA	CAT	Hanibuchi 2011	43	1.251 (1.155-1.356)	<0.001	0.033
General LTPA	CAT	Tsunoda 2012	31	1.146 (1.063-1.234)	<0.001	0.011
General LTPA	CAT	Van Cauwenberg 2012	47	1.201 (1.098-1.314)	<0.001	0.020
General LTPA	CAT	Gao 2015	37	1.188 (1.069-1.319)	0.002	0.033
General LTPA	CAT	McMorris 2015	46	1.218 (1.114-1.332)	<0.001	0.024

Outcome	Scale	Independent study/sample removed	Remaining k	Pooled OR (95% CI)	p	Total tau2
General LTPA	UNIT	Hanibuchi 2011	11	1.228 (0.809-1.864)	0.298	0.100
General LTPA	UNIT	Van Cauwenberg 2012	21	1.215 (0.811-1.819)	0.327	0.107
General LTPA	UNIT	CerinB 2013	16	1.217 (0.808-1.834)	0.324	0.106
General LTPA	UNIT	Kramer 2013	21	1.014 (1.000-1.028)	0.049	0.000

Note. One independent study/sample and all effect estimates contributed by that sample were removed at each iteration, after which the three-level model was refitted. This procedure preserves the study/sample-level clustering structure more appropriately than deleting one effect estimate at a time.

Supplementary Table S10. CAT-UNIT joint sensitivity analyses for LTW, NLTW, and General LTPA.

Panel A. CR2 estimates from separate and joint models

Outcome	Scale	Model	CR2 OR (95% CI)	p	CR2 df
LTW	CAT	Separate	1.103 (1.015-1.200)	0.025	12.14
LTW	CAT	Joint_Common	1.105 (1.014-1.204)	0.027	13.80
LTW	CAT	Joint_ScaleSpecific	1.103 (1.015-1.200)	0.025	12.14
LTW	UNIT	Separate	1.068 (0.989-1.154)	0.084	9.40
LTW	UNIT	Joint_Common	1.077 (1.002-1.157)	0.044	11.87
LTW	UNIT	Joint_ScaleSpecific	1.068 (0.989-1.154)	0.084	9.40
NLTW	CAT	Separate	2.008 (0.385-10.464)	0.210	1.99
NLTW	CAT	Joint_Common	1.723 (0.484-6.130)	0.203	1.96
NLTW	CAT	Joint_ScaleSpecific	2.008 (0.385-10.464)	0.210	1.99
NLTW	UNIT	Separate	1.063 (0.978-1.156)	0.094	2.36
NLTW	UNIT	Joint_Common	1.102 (0.975-1.246)	0.090	3.80
NLTW	UNIT	Joint_ScaleSpecific	1.063 (0.978-1.156)	0.094	2.36
General LTPA	CAT	Separate	1.180 (1.067-1.304)	0.007	5.62
General LTPA	CAT	Joint_Common	1.198 (1.073-1.337)	0.006	7.67
General LTPA	CAT	Joint_ScaleSpecific	1.180 (1.067-1.304)	0.007	5.62
General LTPA	UNIT	Separate	1.154 (0.756-1.761)	0.358	2.97
General LTPA	UNIT	Joint_Common	1.127 (0.998-1.273)	0.053	7.24
General LTPA	UNIT	Joint_ScaleSpecific	1.154 (0.756-1.761)	0.358	2.97

Panel B. Joint-model fit comparison

Outcome	Joint model	k	Parameters	logLik	AIC	BIC	Delta AIC	Delta BIC
LTW	Joint_Common	227	4	6.103	-4.21	9.46	16.31	9.48
LTW	Joint_ScaleSpecific	227	6	16.257	-20.52	-0.02	0.00	0.00
NLTW	Joint_Common	45	4	7.639	-7.28	-0.23	11.39	7.87
NLTW	Joint_ScaleSpecific	45	6	15.336	-18.67	-8.10	0.00	0.00
General LTPA	Joint_Common	72	4	50.710	-93.42	-84.42	0.45	0.00
General LTPA	Joint_ScaleSpecific	72	6	52.932	-93.86	-80.37	0.00	4.05

Note. Joint_Common assumes a common heterogeneity structure for CAT and UNIT, whereas Joint_ScaleSpecific allows scale-specific heterogeneity components. Joint models include effect scale as a moderator and are used only to assess whether scale-specific CAT and UNIT estimates remain consistent with their separate models; they are not interpreted as a single pooled overall OR across CAT and UNIT. Delta AIC/BIC are calculated relative to the better-fitting model within each outcome.

Supplementary Table S11. Adapted GRADE assessment by outcome and built-environment domain for the certainty of evidence on associations between community built environment and leisure-time physical activity among older adults

Outcome	Environmental domain	Risk of bias	Inconsistency	Indirectness	Imprecision	Publication bias	Overall certainty	Summary interpretation
**LTW**	TS (Traffic safety)	Not serious	Serious	Not serious	Not serious	Undetected	Moderate (⊕⊕⊕◯)	Comparatively consistent and precise evidence; findings were generally stable across robust and sensitivity analyses.
	DA (Destination accessibility)	Not serious	Serious	Not serious	Not serious	Undetected	Moderate (⊕⊕⊕◯)	Comparatively consistent and precise evidence; findings were generally stable across robust and sensitivity analyses.
	ES (Environmental safety)	Not serious	Serious	Not serious	Serious	Undetected	Low (⊕⊕◯◯)	Confidence reduced by residual heterogeneity, smaller evidence bases and/or imprecision.

Outcome	Environmental domain	Risk of bias	Inconsistency	Indirectness	Imprecision	Publication bias	Overall certainty	Summary interpretation
	AE (Aesthetics)	Not serious	Serious	Not serious	Not serious	Undetected	Moderate (⊕⊕⊕◯)	Comparatively consistent and precise evidence; findings were generally stable across robust and sensitivity analyses.
	PG (Parks and green space)	Not serious	Serious	Not serious	Not serious	Undetected	Moderate (⊕⊕⊕◯)	Comparatively consistent and precise evidence; findings were generally stable across robust and sensitivity analyses.
	RD (Residential density)	Not serious	Serious	Not serious	Not serious	Undetected	Moderate (⊕⊕⊕◯)	Comparatively consistent and precise evidence; findings were generally stable across robust and sensitivity analyses.
	LUM (Land use mix)	Not serious	Serious	Not serious	Serious	Undetected	Low (⊕⊕◯◯)	Confidence reduced by residual heterogeneity, smaller evidence bases and/or imprecision.
	SC (Street connectivity)	Not serious	Serious	Not serious	Serious	Undetected	Low (⊕⊕◯◯)	Confidence reduced by residual heterogeneity, smaller evidence bases and/or imprecision.
	SQ (Sidewalk quality)	Not serious	Serious	Not serious	Serious	Undetected	Low (⊕⊕◯◯)	Confidence reduced by residual heterogeneity, smaller evidence bases and/or imprecision.
**NLTW**	TS (Traffic safety)	Not serious	Serious	Not serious	Not serious	Undetected	Moderate (⊕⊕⊕◯)	Comparatively more stable evidence within NLTW, but the evidence base remained limited.
	DA (Destination accessibility)	Not serious	Serious	Not serious	Not serious	Undetected	Moderate (⊕⊕⊕◯)	Comparatively more stable evidence within NLTW, but the evidence base remained limited.
	ES (Environmental safety)	Not serious	Serious	Not serious	Serious	Undetected	Low (⊕⊕◯◯)	Confidence reduced by residual heterogeneity, smaller evidence bases and/or imprecision.
	AE (Aesthetics)	Not serious	Serious	Not serious	Serious	Undetected	Low (⊕⊕◯◯)	Confidence reduced by residual heterogeneity, smaller evidence bases and/or imprecision.
	PG (Parks and green space)	Not serious	Serious	Not serious	Serious	Undetected	Low (⊕⊕◯◯)	Confidence reduced by residual heterogeneity, smaller evidence bases and/or imprecision.
	RD (Residential density)	Not serious	Serious	Not serious	Serious	Undetected	Low (⊕⊕◯◯)	Confidence reduced by residual heterogeneity, smaller evidence bases and/or imprecision.
	LUM (Land use mix)	Not serious	Serious	Not serious	Serious	Undetected	Low (⊕⊕◯◯)	Confidence reduced by residual heterogeneity, smaller evidence bases and/or imprecision.
	SC (Street connectivity)	Not serious	Serious	Not serious	Serious	Undetected	Low (⊕⊕◯◯)	Confidence reduced by residual heterogeneity, smaller evidence bases and/or imprecision.
	SQ (Sidewalk quality)	Not serious	Serious	Not serious	Serious	Undetected	Low (⊕⊕◯◯)	Confidence reduced by residual heterogeneity, smaller evidence bases and/or imprecision.
**General LTPA**	TS (Traffic safety)	Not serious	Serious	Not serious	Not serious	Undetected	Moderate (⊕⊕⊕◯)	Comparatively consistent and precise evidence; findings were generally stable across robust and sensitivity analyses.
	DA (Destination accessibility)	Not serious	Serious	Not serious	Not serious	Undetected	Moderate (⊕⊕⊕◯)	Comparatively consistent and precise evidence; findings were generally stable across robust and sensitivity analyses.
	ES (Environmental safety)	Not serious	Serious	Not serious	Serious	Undetected	Low (⊕⊕◯◯)	Confidence reduced by residual heterogeneity, smaller evidence bases and/or imprecision.
	AE (Aesthetics)	Not serious	Serious	Not serious	Not serious	Undetected	Moderate (⊕⊕⊕◯)	Comparatively consistent and precise evidence; findings were generally stable across robust and sensitivity analyses.
	PG (Parks and green space)	Not serious	Serious	Not serious	Not serious	Undetected	Moderate (⊕⊕⊕◯)	Comparatively consistent and precise evidence; findings were generally stable across robust and sensitivity analyses.
	RD (Residential density)	Not serious	Serious	Not serious	Not serious	Undetected	Moderate (⊕⊕⊕◯)	Comparatively consistent and precise evidence; findings were generally stable across robust and sensitivity analyses.
	LUM (Land use mix)	Not serious	Serious	Not serious	Serious	Undetected	Low (⊕⊕◯◯)	Confidence reduced by residual heterogeneity, smaller evidence bases and/or imprecision.

Outcome	Environmental domain	Risk of bias	Inconsistency	Indirectness	Imprecision	Publication bias	Overall certainty	Summary interpretation
	SC (Street connectivity)	Not serious	Serious	Not serious	Serious	Undetected	Low (⊕⊕◯◯)	Confidence reduced by residual heterogeneity, smaller evidence bases and/or imprecision.
	SQ (Sidewalk quality)	Not serious	Serious	Not serious	Serious	Undetected	Low (⊕⊕◯◯)	Confidence reduced by residual heterogeneity, smaller evidence bases and/or imprecision.

Notes: GRADE = Grading of Recommendations Assessment, Development and Evaluation; LTW = leisure-time walking; NLTW = neighbourhood leisure-time walking; General LTPA = general leisure-time physical activity; TS = traffic safety; DA = destination accessibility; ES = environmental safety; AE = aesthetics; PG = parks and green space; RD = residential density; LUM = land use mix; SC = street connectivity; SQ = sidewalk quality. This table presents an adapted GRADE assessment for this observational association review. Certainty was judged for each outcome-domain combination considering risk of bias, inconsistency, indirectness, imprecision, publication bias, 95% prediction intervals, CR2 robust inference and sensitivity analyses. Moderate certainty was assigned where evidence was comparatively stronger, more precise and more stable; low certainty was assigned where residual heterogeneity, smaller evidence bases and/or imprecision reduced confidence. No combination was rated high because all included studies were cross-sectional and residual uncertainty remained.

Supplementary Figure S1.

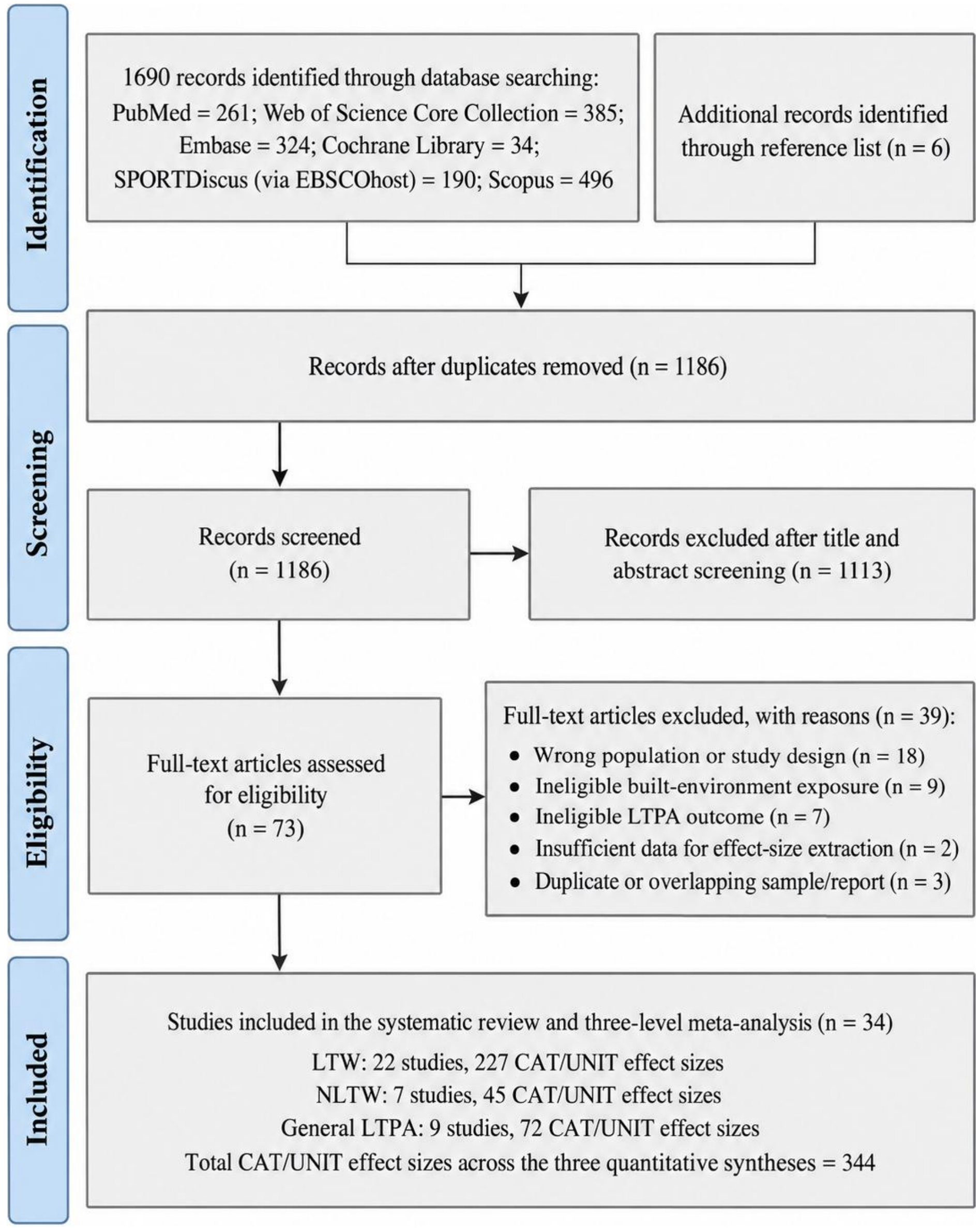

Supplementary Figure S1. PRISMA flow diagram of the study-selection process.

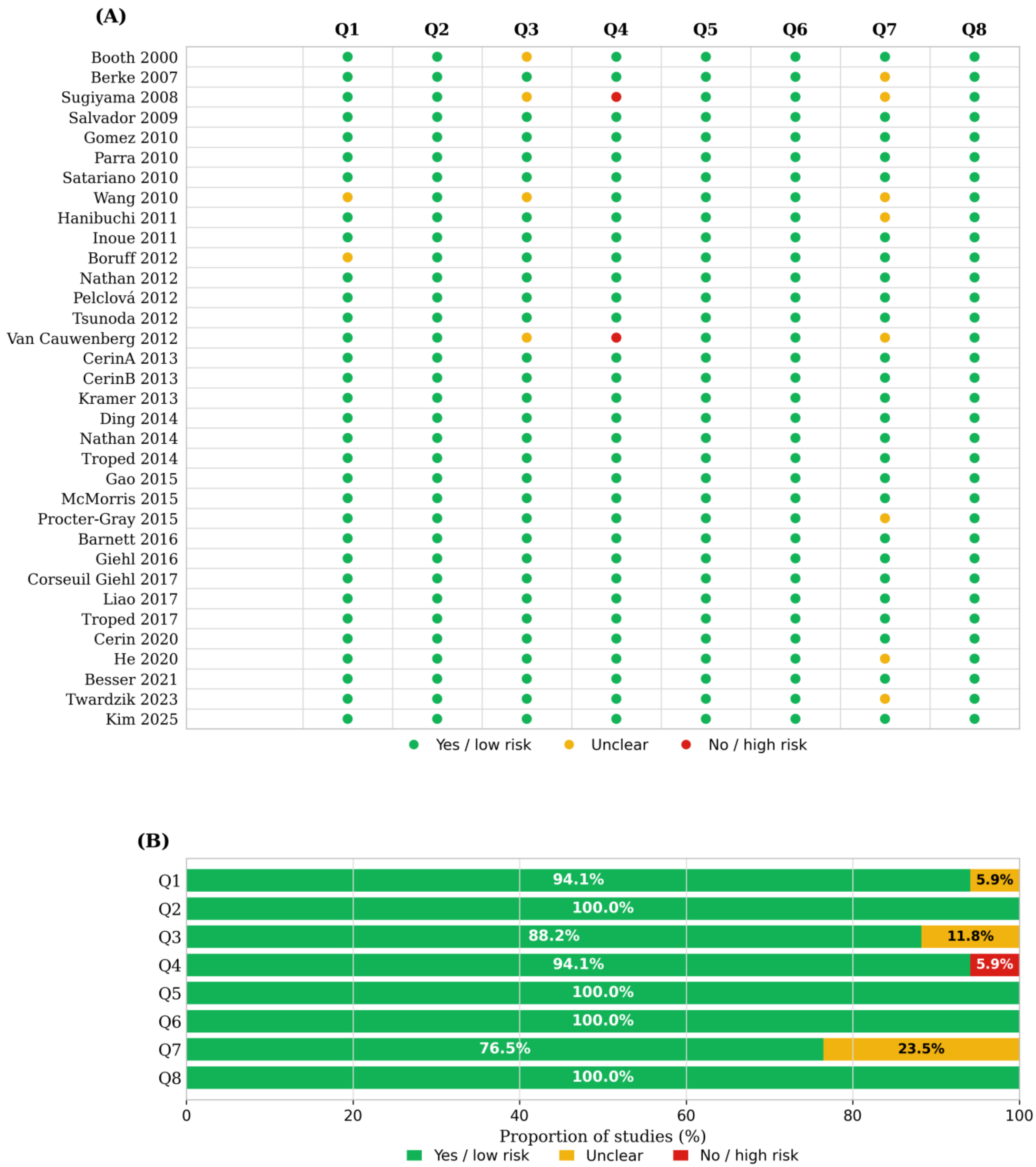
(A)
Q1
Q2
Q3
Q4
Q5
Q6
Q7
Q8
Booth 2000
Berke 2007
Sugiyama 2008
Salvador 2009
Gomez 2010
Parra 2010
Satariano 2010
Wang 2010
Hanibuchi 2011
Inoue 2011
Boruff 2012
Nathan 2012
Pelclová 2012
Tsunoda 2012
Van Cauwenberg 2012
CerinA 2013
CerinB 2013
Kramer 2013
Ding 2014
Nathan 2014
Troped 2014
Gao 2015
McMorris 2015
Procter-Gray 2015
Barnett 2016
Giehl 2016
Corseuil Giehl 2017
Liao 2017
Troped 2017
Cerin 2020
He 2020
Besser 2021
Twardzik 2023
Kim 2025
Yes / low risk
Unclear
No / high risk
(B)
94.1%
5.9%
100.0%
88.2%
11.8%
94.1%
5.9%
100.0%
100.0%
76.5%
23.5%
100.0%
0
20
40
60
80
100
Proportion of studies (%)
Yes / low risk
Unclear
No / high risk

Supplementary Figure S2. Methodological quality assessment of the included studies using the JBI critical appraisal checklist: (A) study-level ratings across the eight checklist items; (B) summary proportions for each checklist item.

A

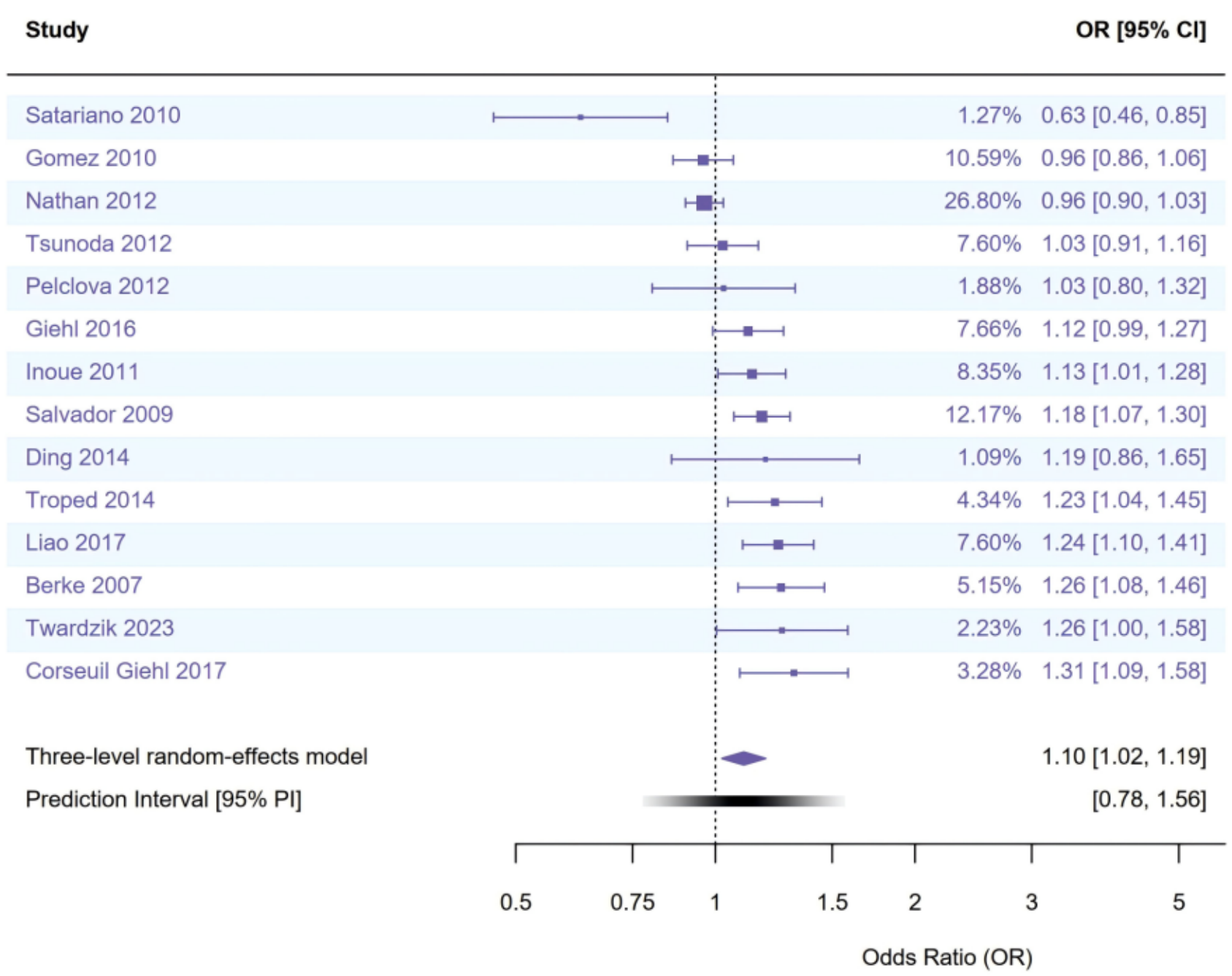

B

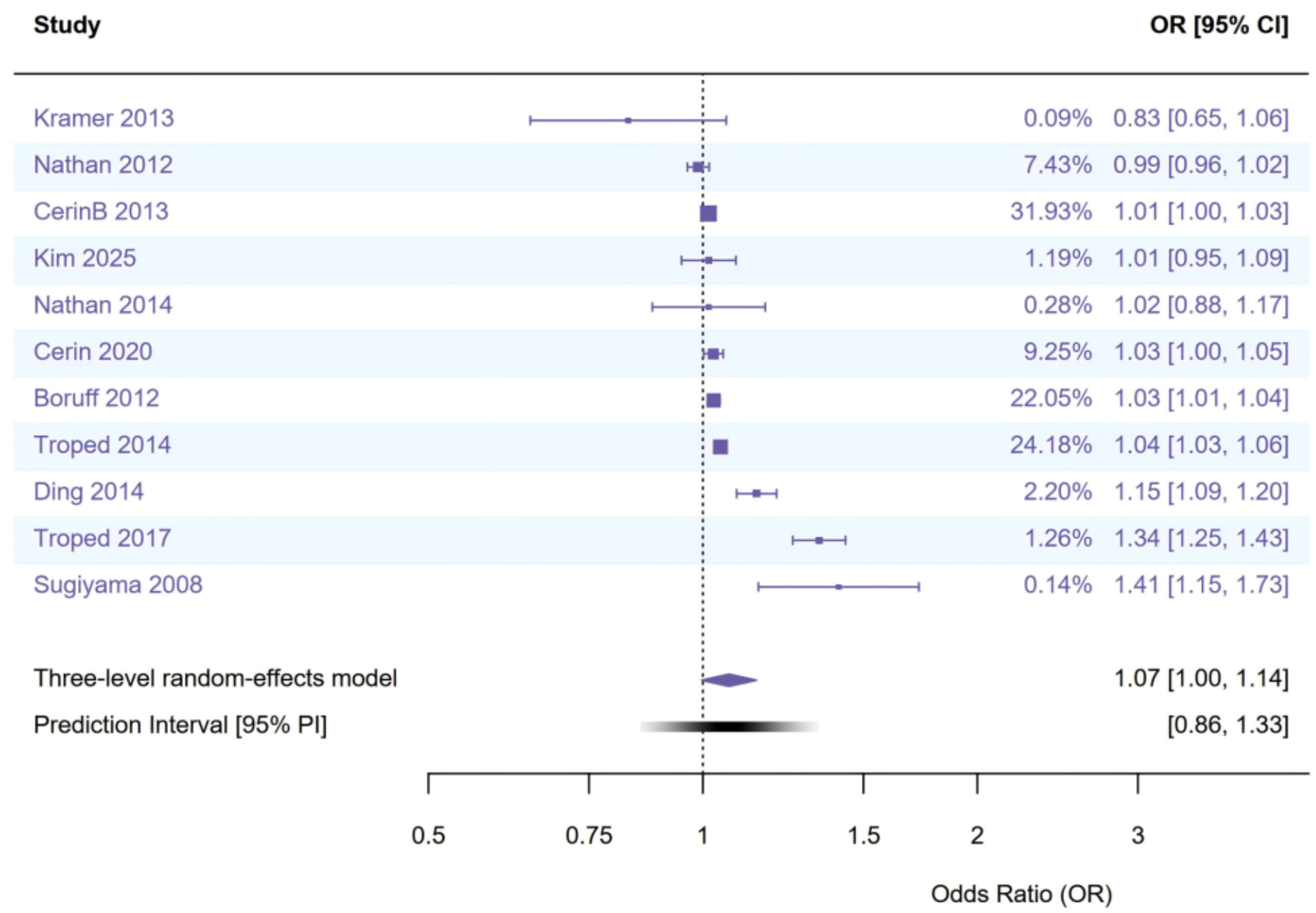

C

D

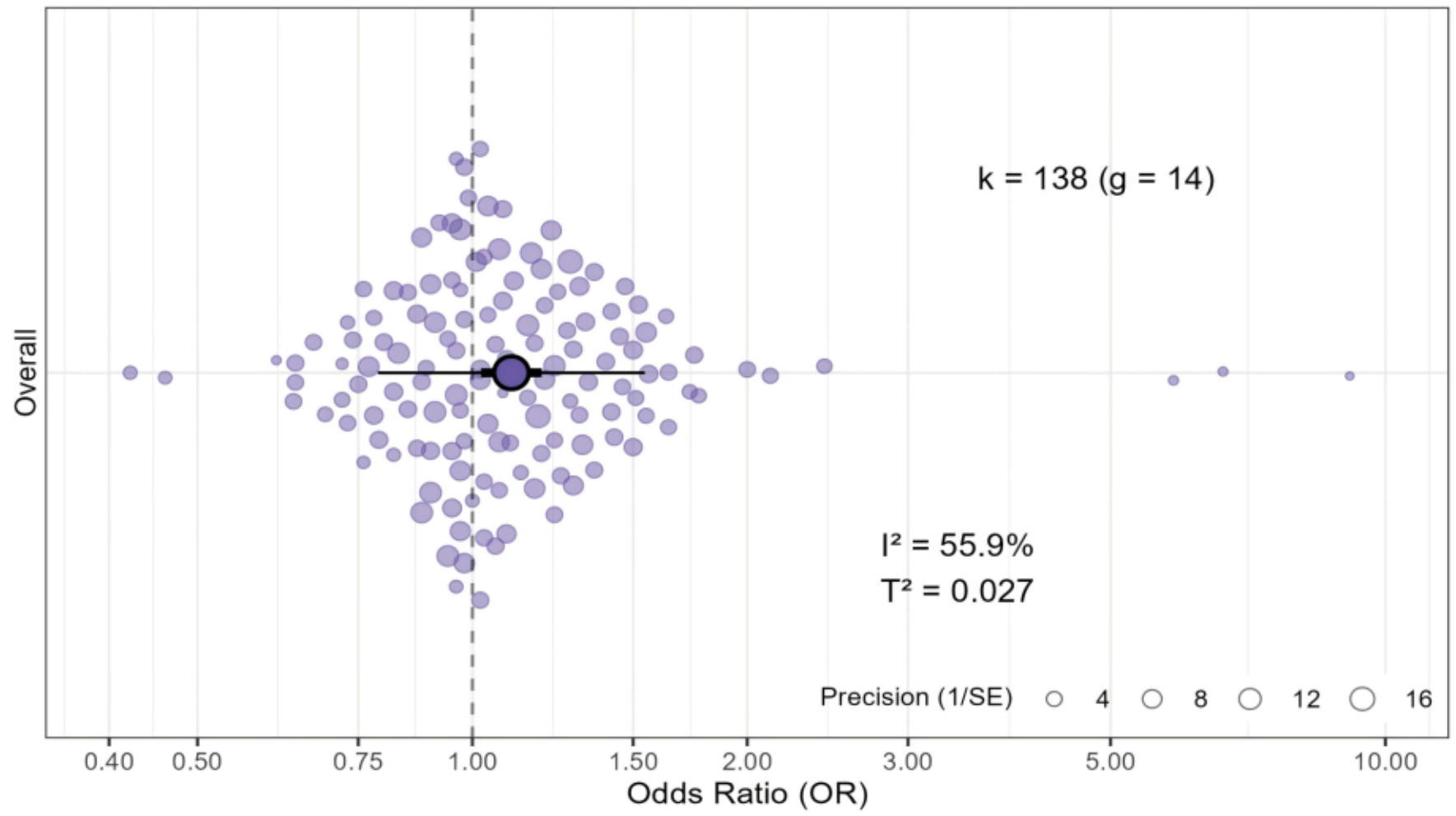

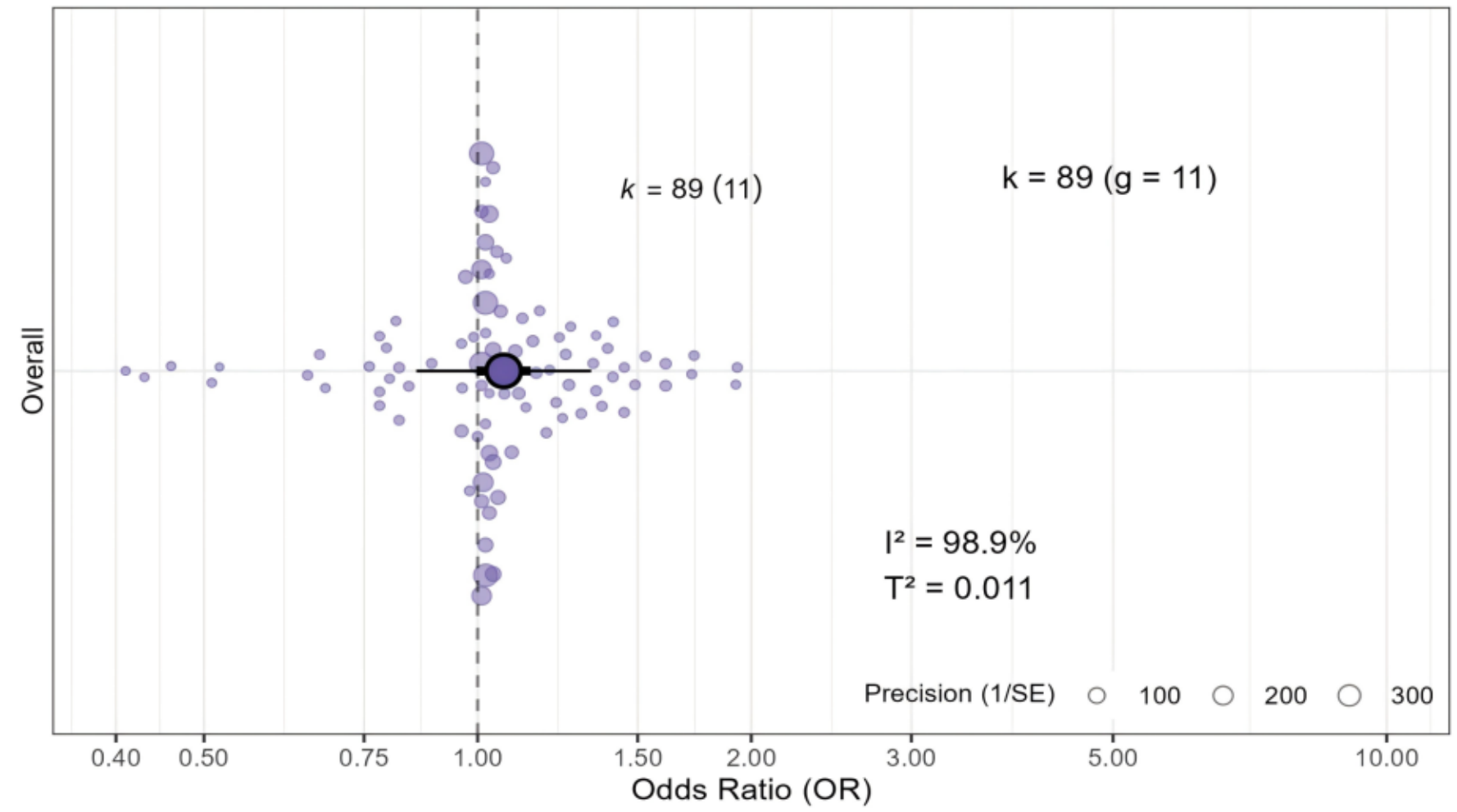

E

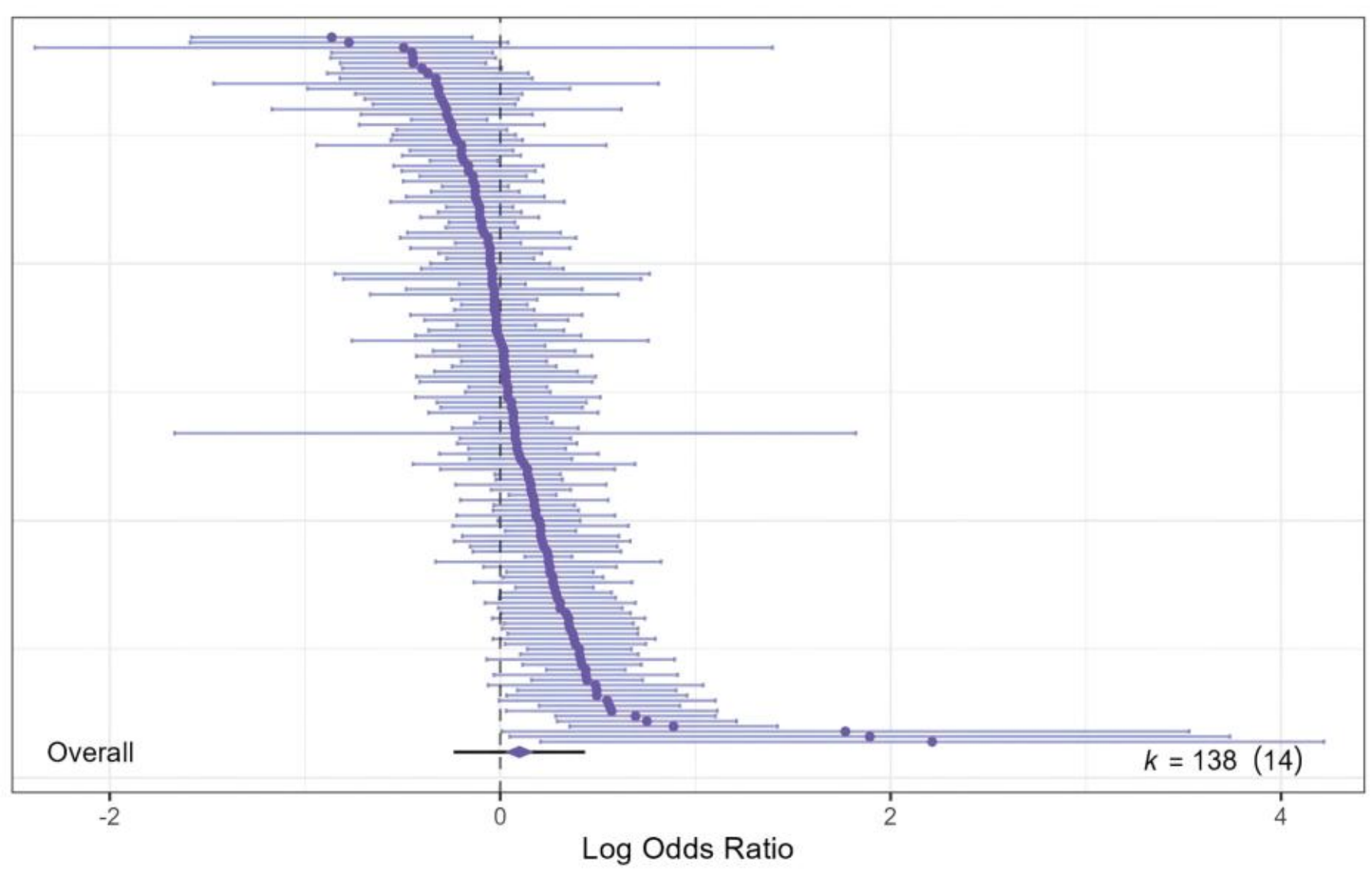

F

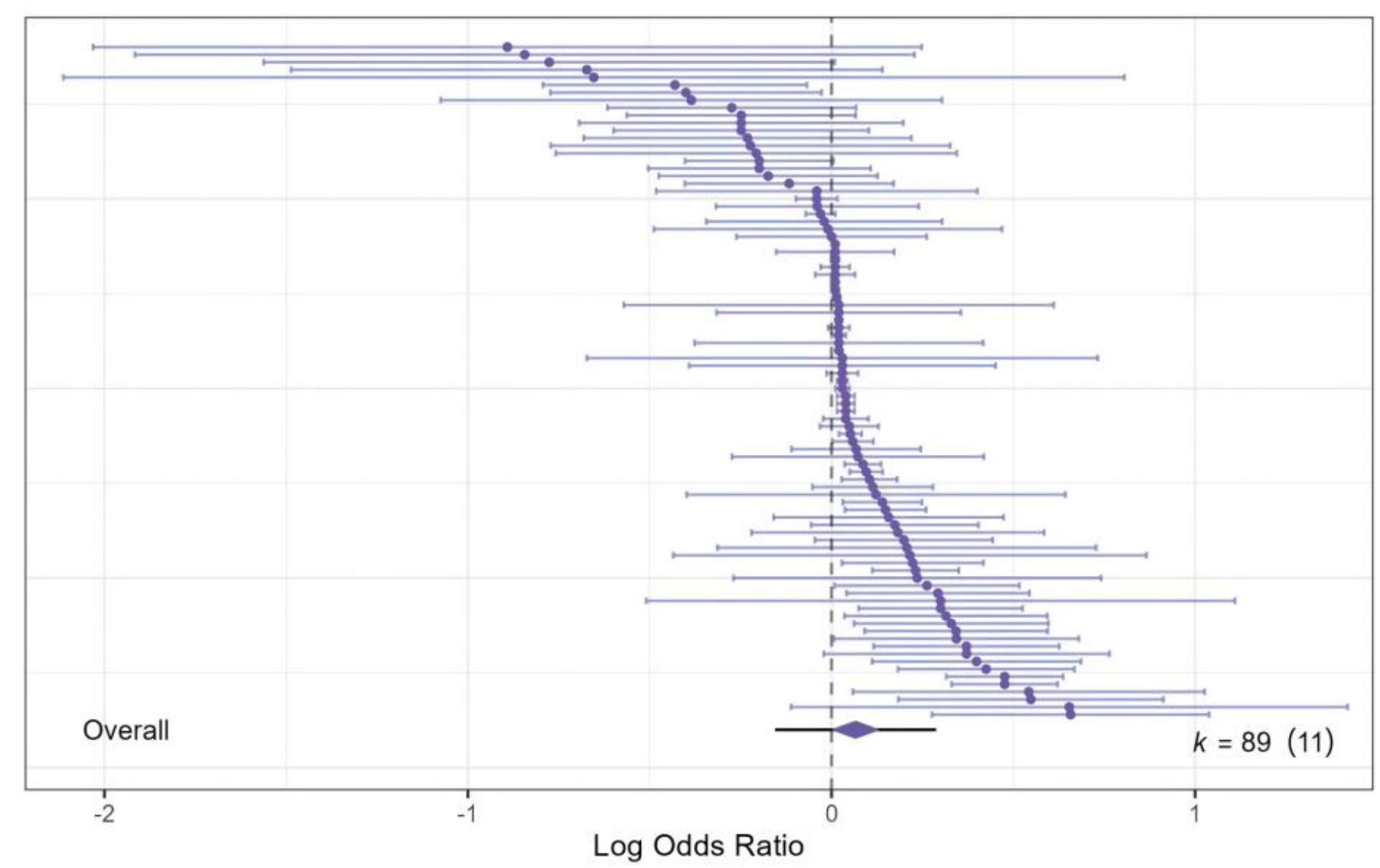

Supplementary Figure S3. Leisure-time walking (LTW): overall three-level meta-analysis plots. A, CAT forest plot; B, UNIT forest plot; C, CAT annotated orchard plot; D, UNIT annotated orchard plot; E, CAT caterpillar plot; F, UNIT caterpillar plot.

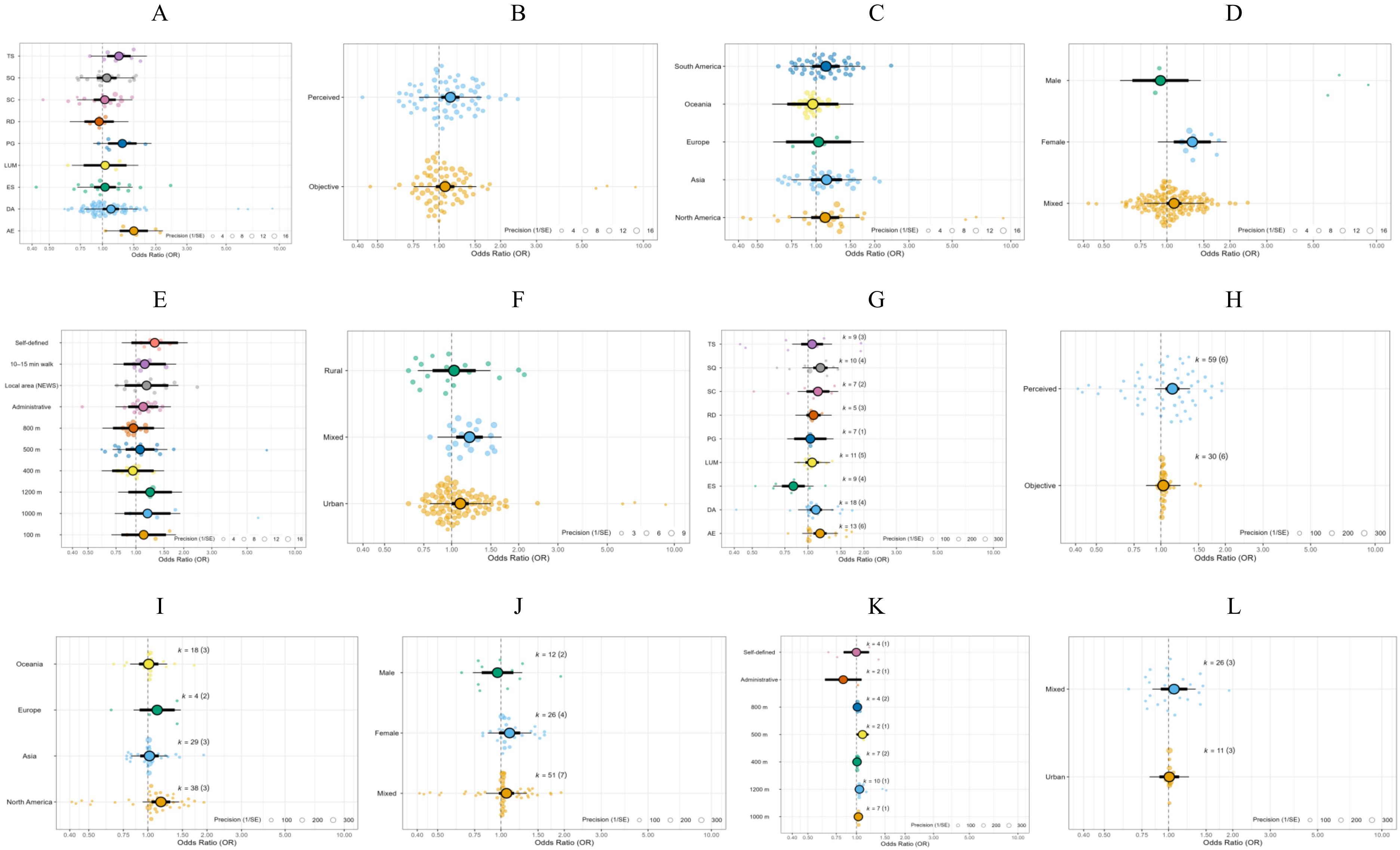

Supplementary Figure S4. Leisure-time walking (LTW): subgroup analyses. A, CAT by built-environment domain; B, CAT by environment-measure type; C, CAT by region; D, CAT by sex composition; E, CAT by spatial scale; F, CAT by urbanicity; G, UNIT by built-environment domain; H, UNIT by environment-measure type; I, UNIT by region; J, UNIT by sex composition; K, UNIT by spatial scale; L, UNIT by urbanicity.

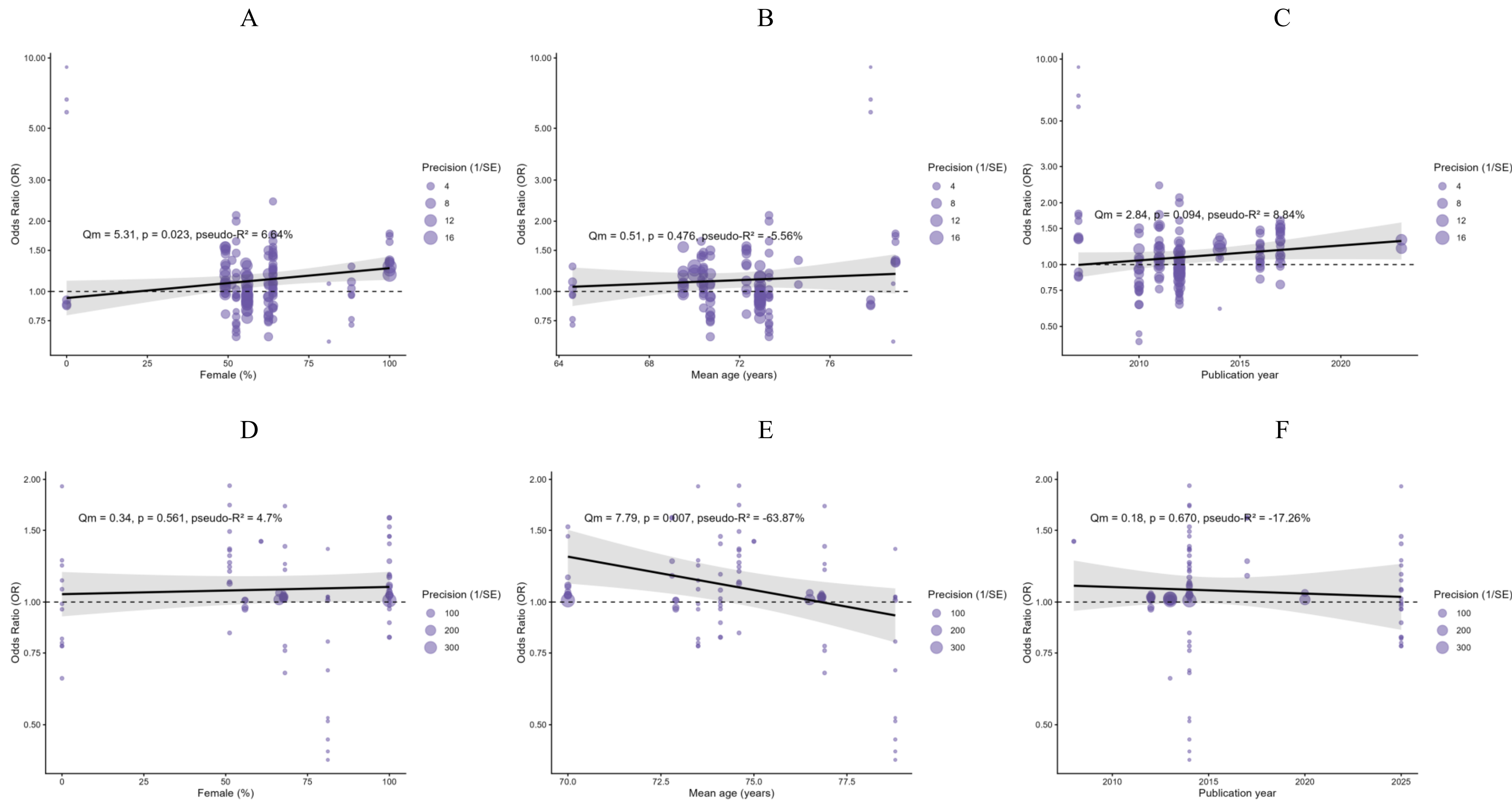

Supplementary Figure S5. Leisure-time walking (LTW): meta-regression plots. A, CAT by female percentage; B, CAT by mean age; C, CAT by publication year; D, UNIT by female percentage; E, UNIT by mean age; F, UNIT by publication year.

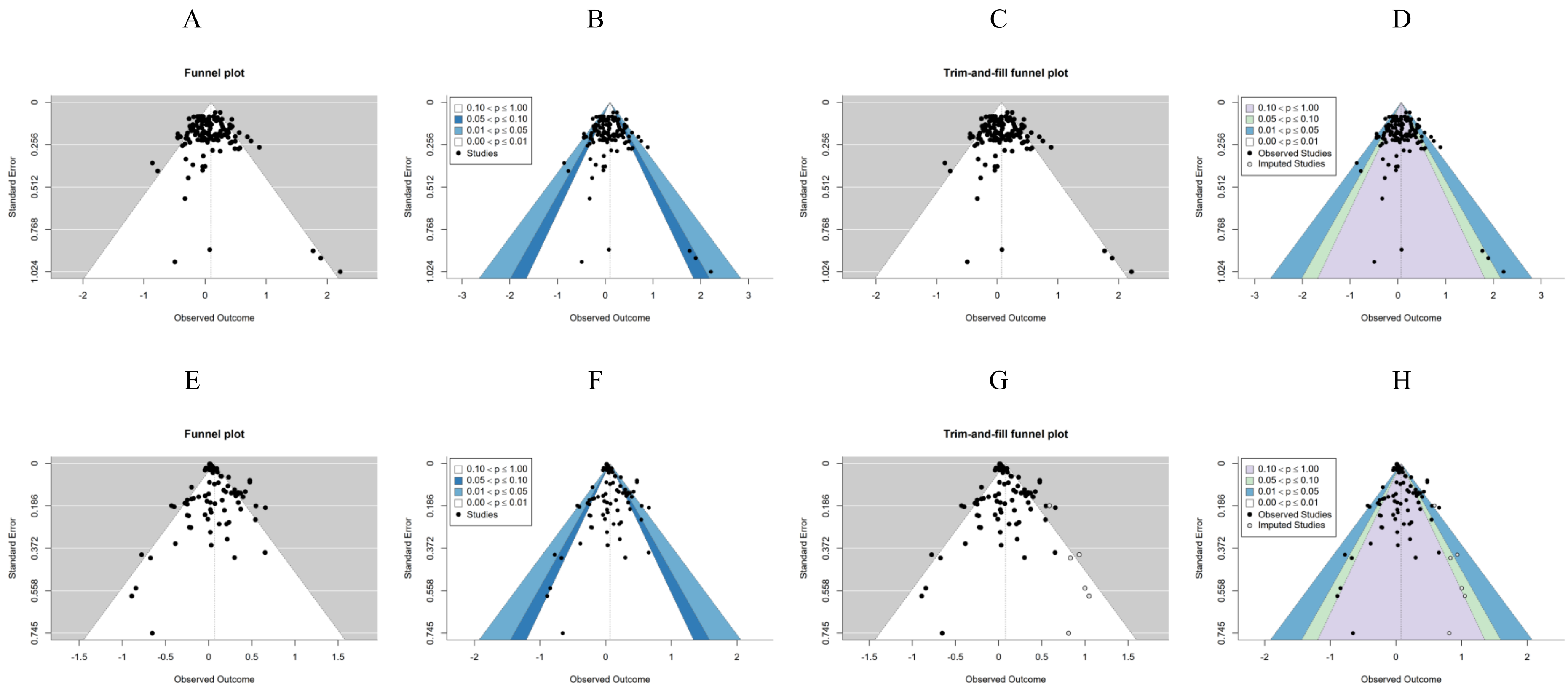

Supplementary Figure S6. Leisure-time walking (LTW): publication-bias and small-study-effect diagnostics. A, CAT basic funnel plot; B, CAT contour-enhanced funnel plot; C, CAT trim-and-fill basic funnel plot; D, CAT trim-and-fill contour-enhanced funnel plot; E, UNIT basic funnel plot; F, UNIT contour-enhanced funnel plot; G, UNIT trim-and-fill basic funnel plot; H, UNIT trim-and-fill contour-enhanced funnel plot.

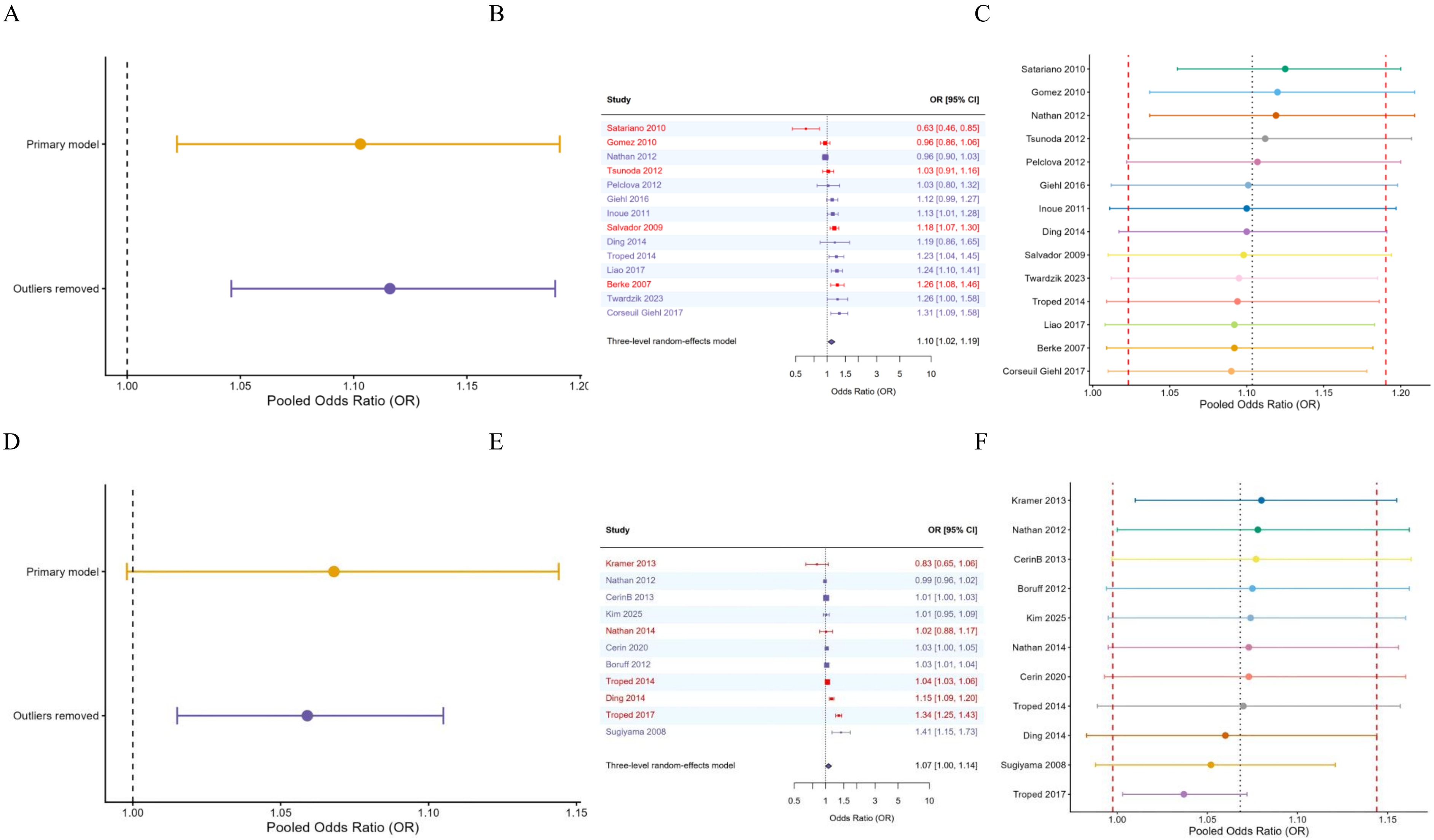

Supplementary Figure S7. Leisure-time walking (LTW): sensitivity analyses. A, CAT comparison before and after outlier removal; B, CAT outlier-identification forest plot; C, CAT leave-one-study/sample-out analysis; D, UNIT comparison before and after outlier removal; E, UNIT outlier-identification forest plot; F, UNIT leave-one-study/sample-out analysis.

A

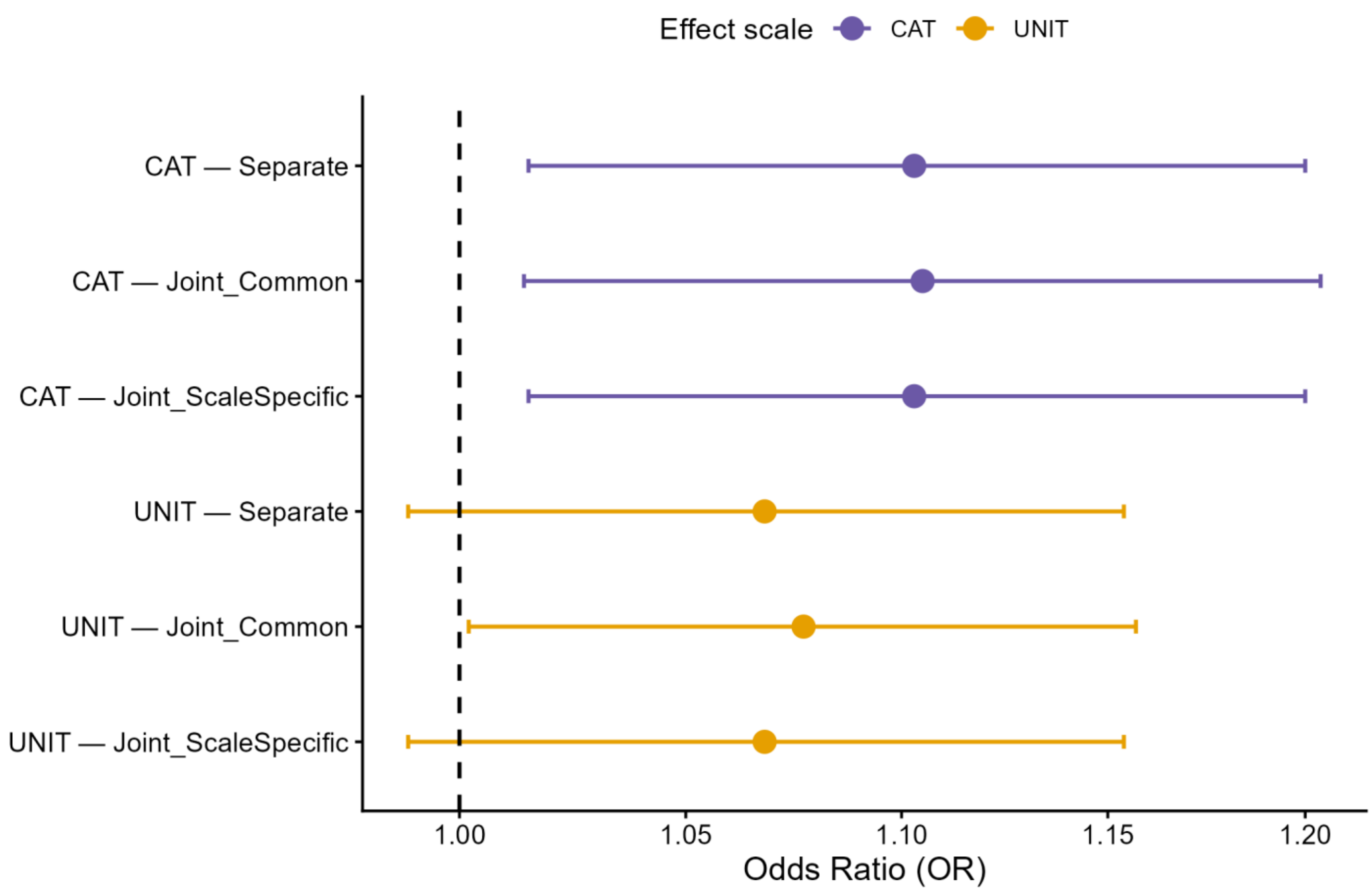

Supplementary Figure S8. Leisure-time walking (LTW): CAT–UNIT joint sensitivity analysis. A, comparison of separate and joint CR2 estimates.

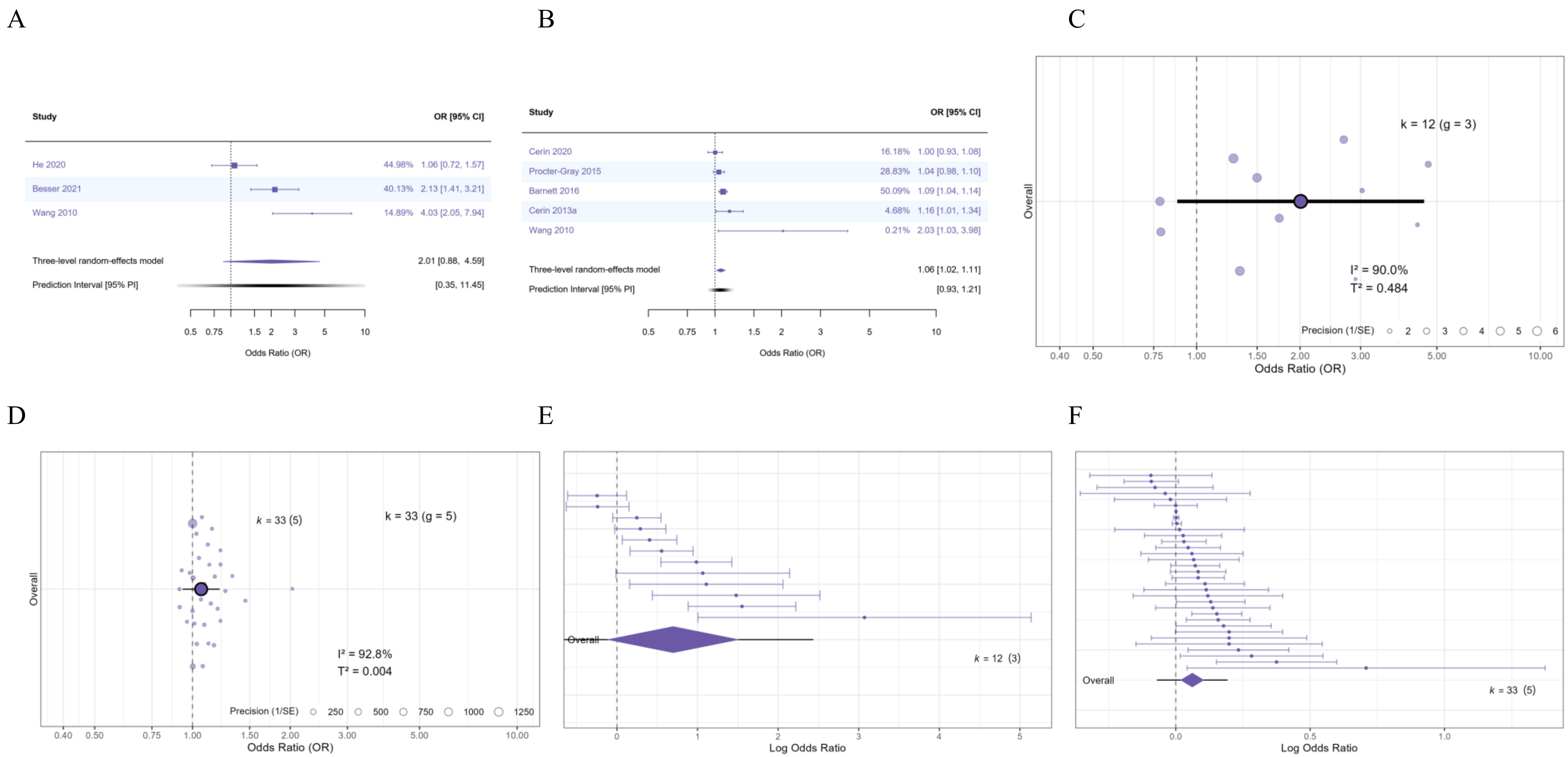

Supplementary Figure S9. Neighbourhood leisure-time walking (NLTW): overall three-level meta-analysis plots. A, CAT forest plot; B, UNIT forest plot; C, CAT annotated orchard plot; D, UNIT annotated orchard plot; E, CAT caterpillar plot; F, UNIT caterpillar plot.

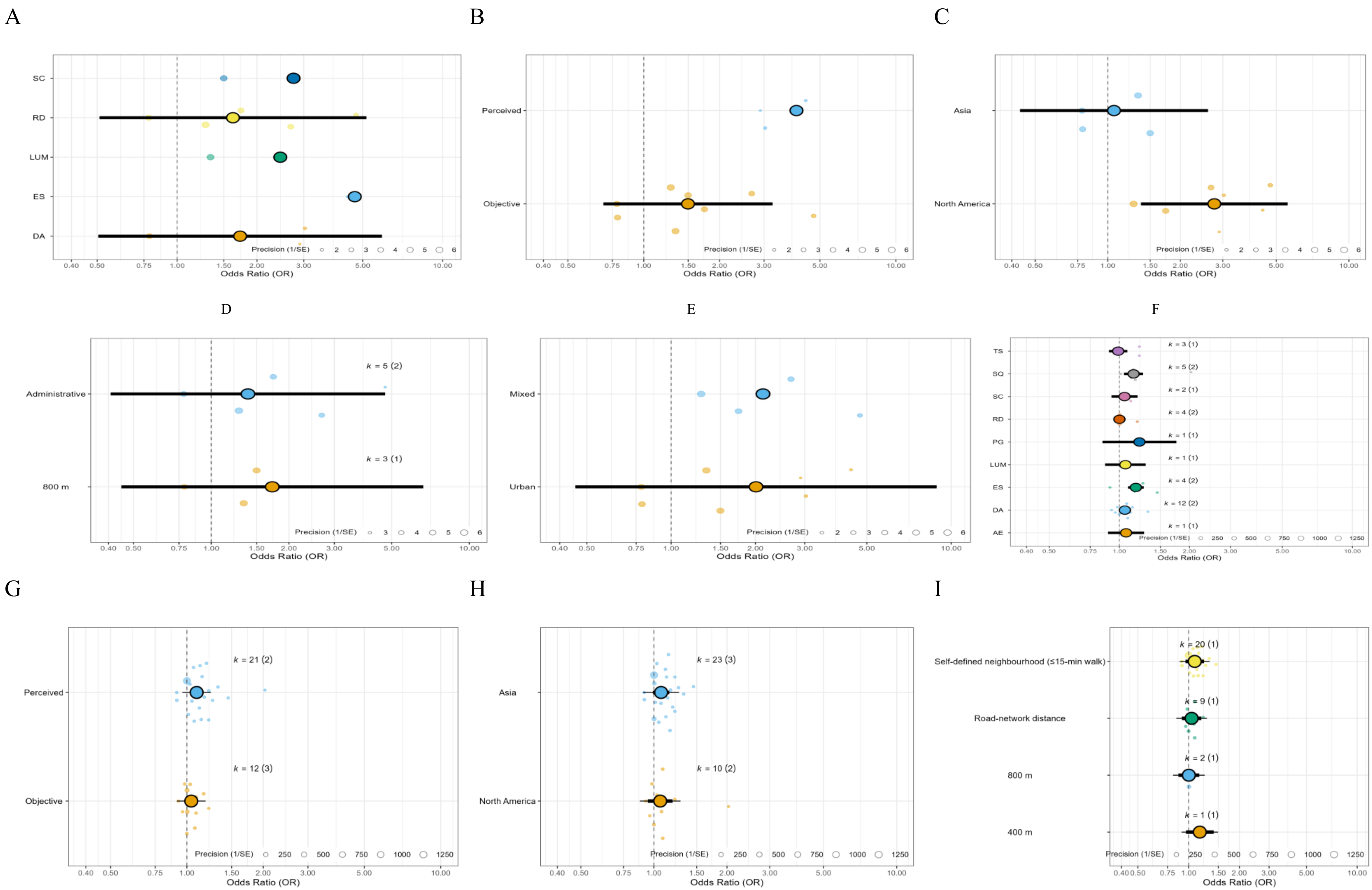

Supplementary Figure S10. Neighbourhood leisure-time walking (NLTW): subgroup analyses. A, CAT by built-environment domain; B, CAT by environment-measure type; C, CAT by region; D, CAT by spatial scale; E, CAT by urbanicity; F, UNIT by built-environment domain; G, UNIT by environment-measure type; H, UNIT by region; I, UNIT by spatial scale.

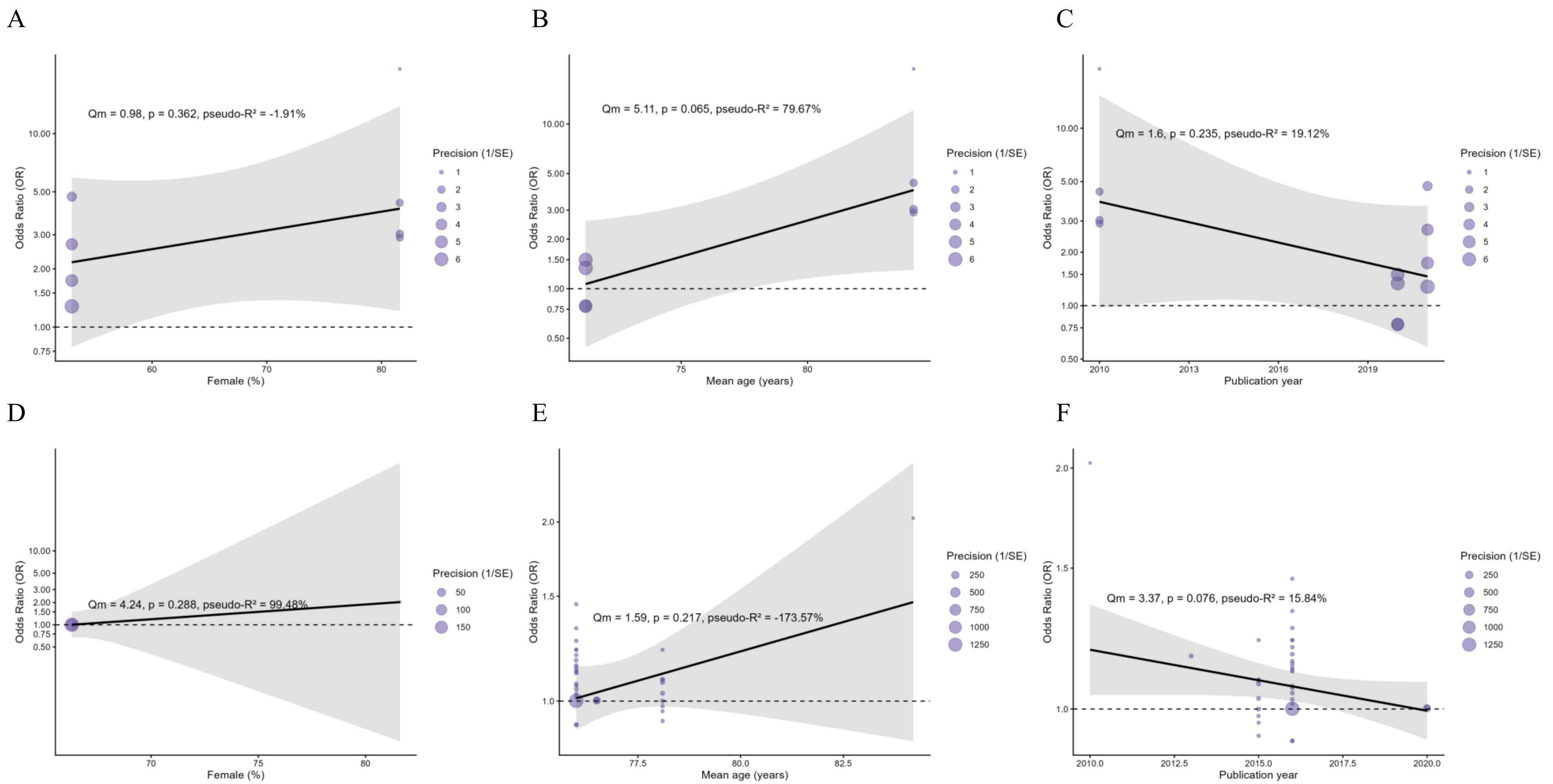

Supplementary Figure S11. Neighbourhood leisure-time walking (NLTW): meta-regression plots. A, CAT by female percentage; B, CAT by mean age; C, CAT by publication year; D, UNIT by female percentage; E, UNIT by mean age; F, UNIT by publication year. These analyses are exploratory because the number of independent study samples was small.

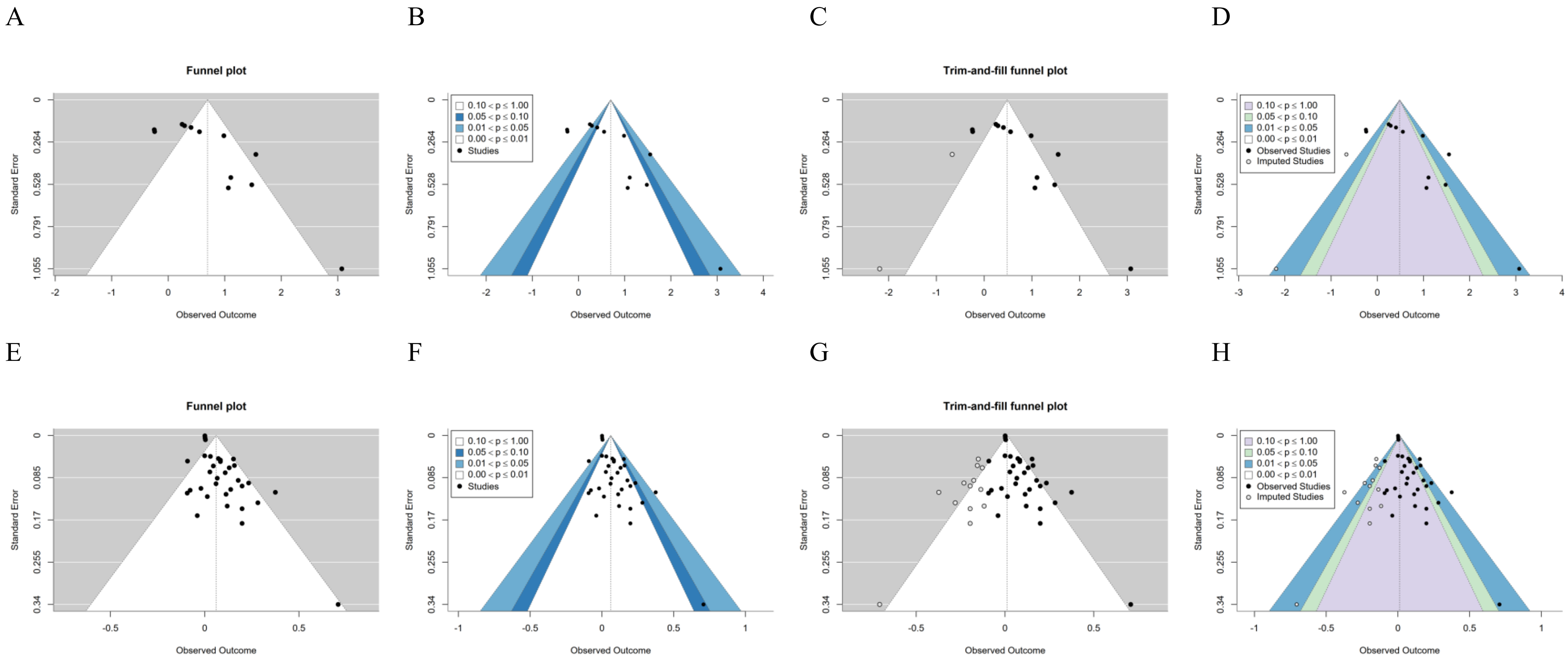

Supplementary Figure S12. Neighbourhood leisure-time walking (NLTW): publication-bias and small-study-effect diagnostics. A, CAT basic funnel plot; B, CAT contour-enhanced funnel plot; C, CAT trim-and-fill basic funnel plot; D, CAT trim-and-fill contour-enhanced funnel plot; E, UNIT basic funnel plot; F, UNIT contour-enhanced funnel plot; G, UNIT trim-and-fill basic funnel plot; H, UNIT trim-and-fill contour-enhanced funnel plot.

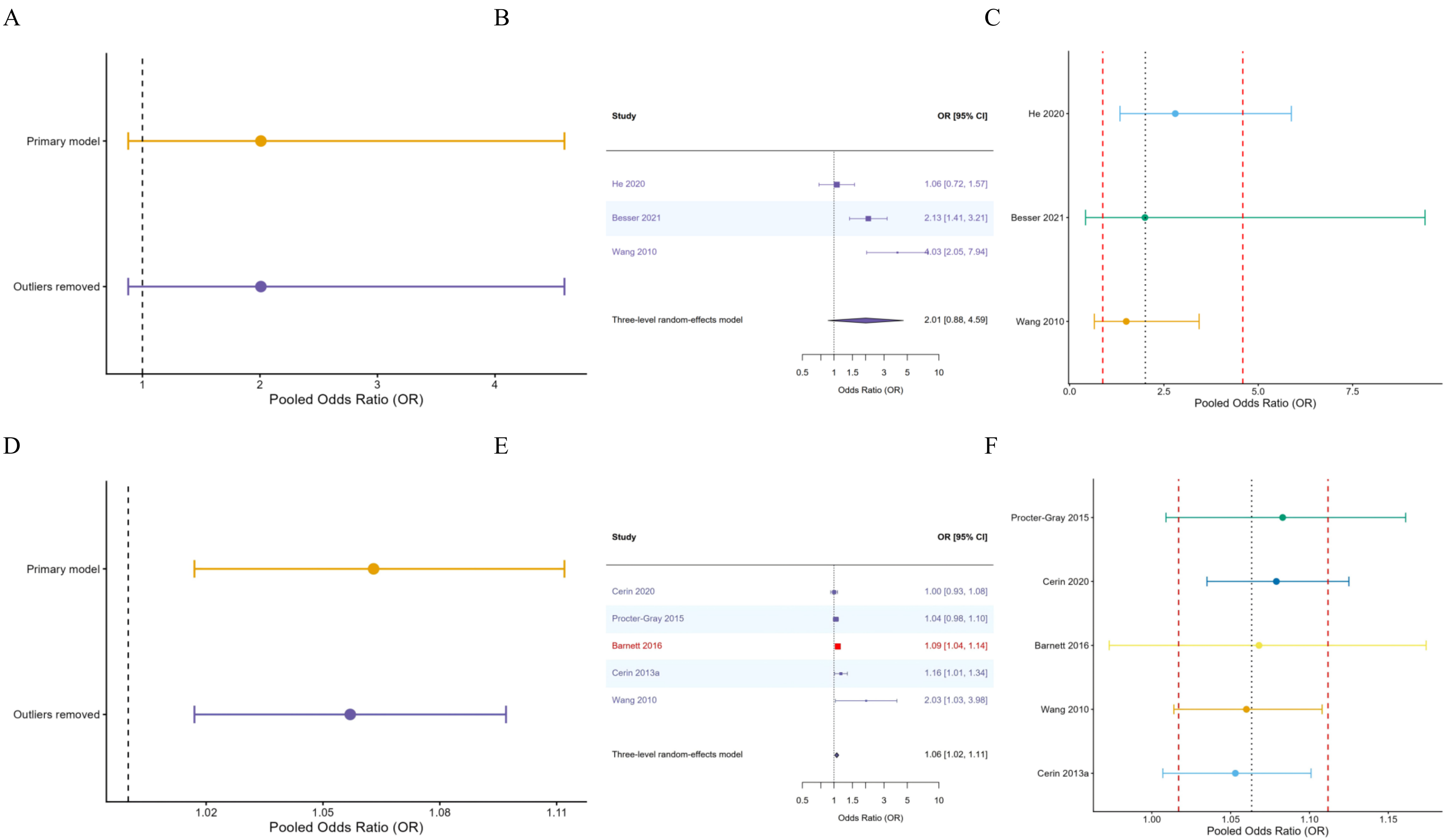

Supplementary Figure S13. Neighbourhood leisure-time walking (NLTW): sensitivity analyses. A, CAT comparison before and after outlier removal; B, CAT outlier-identification forest plot; C, CAT leave-one-study/sample-out analysis; D, UNIT comparison before and after outlier removal; E, UNIT outlier-identification forest plot; F, UNIT leave-one-study/sample-out analysis.

A

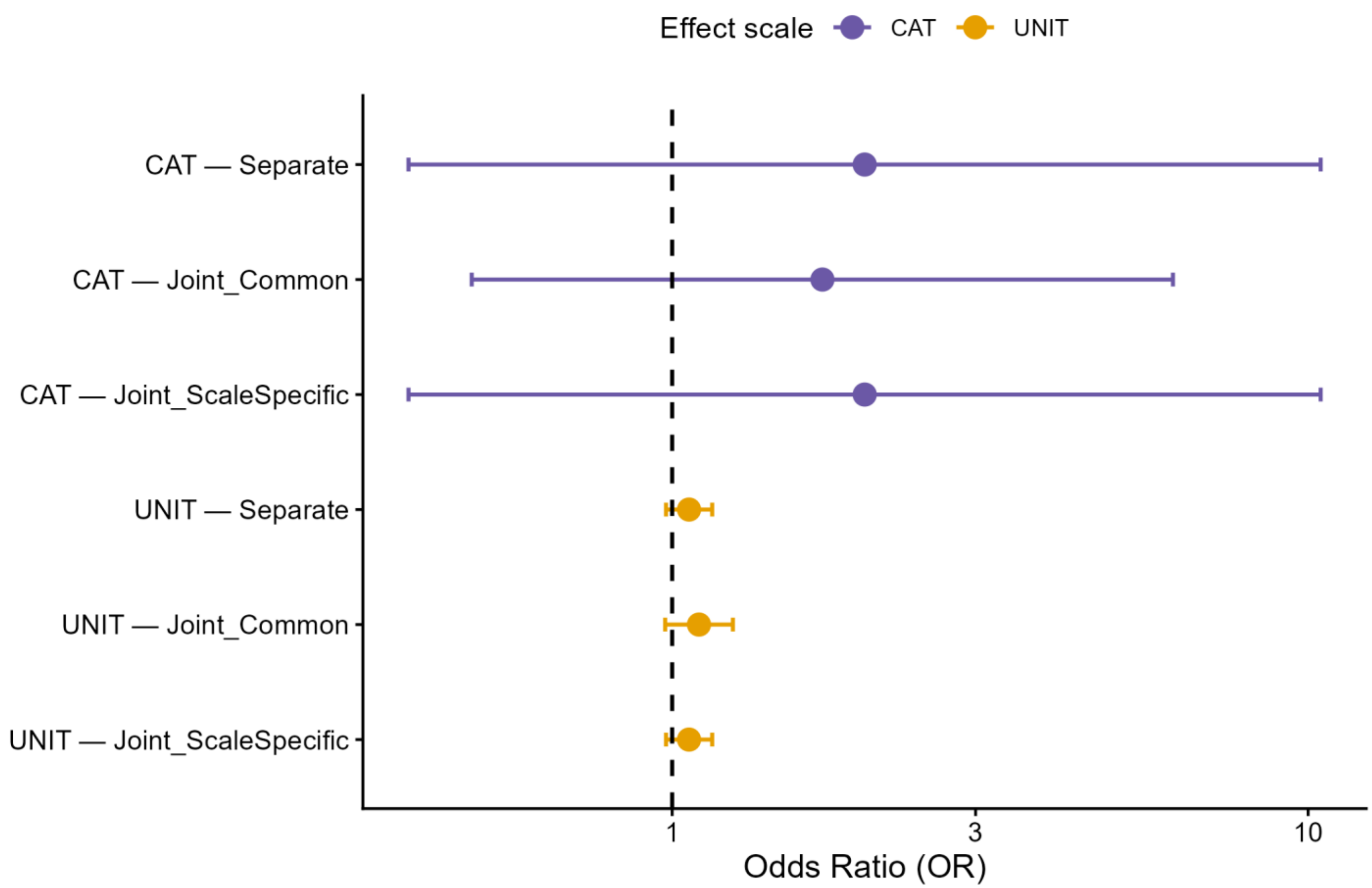

Supplementary Figure S14. Neighbourhood leisure-time walking (NLTW): CAT–UNIT joint sensitivity analysis. A, comparison of separate and joint CR2 estimates.

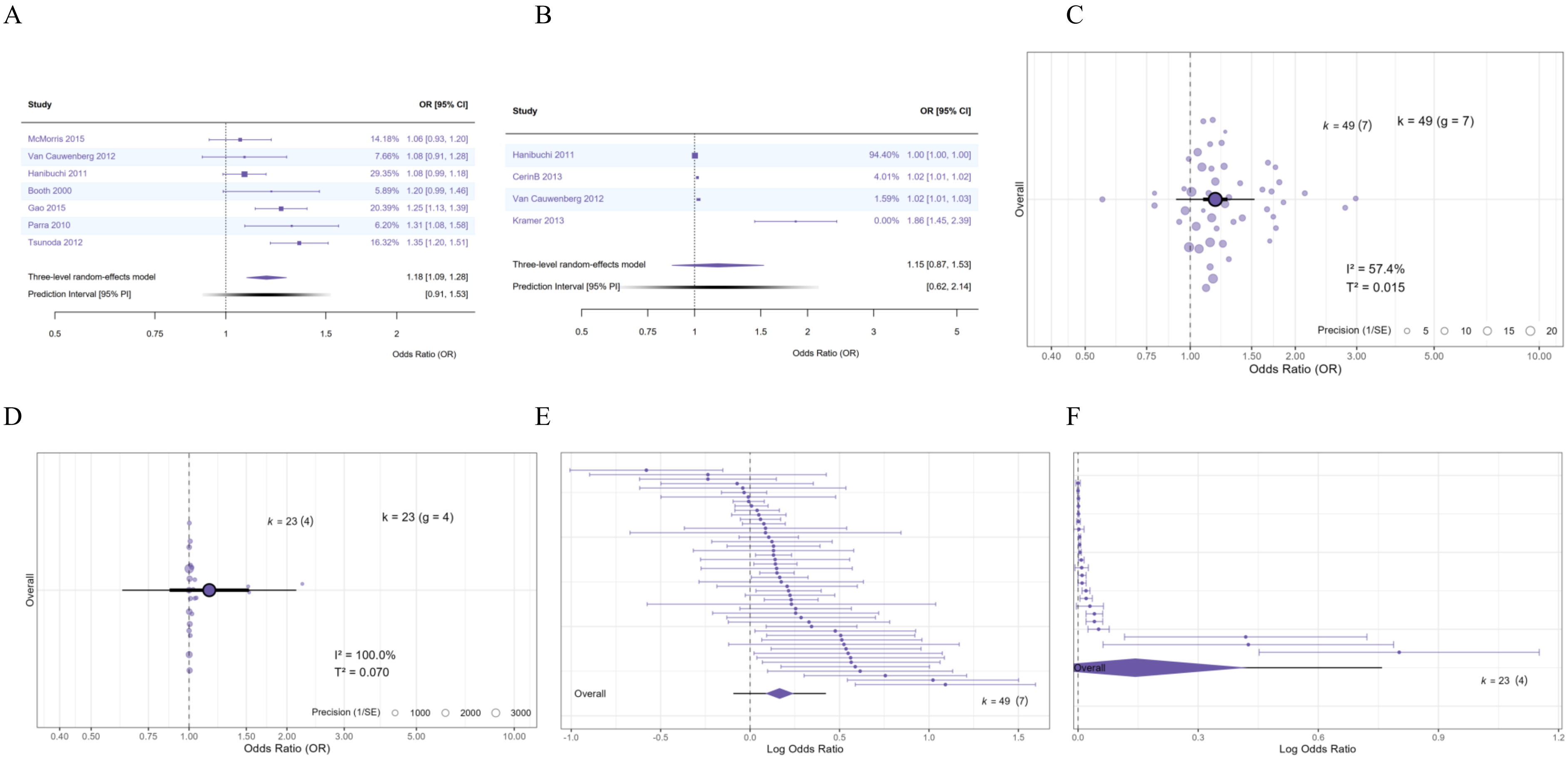

Supplementary Figure S15. General leisure-time physical activity (General LTPA): overall three-level meta-analysis plots. A, CAT forest plot; B, UNIT forest plot; C, CAT annotated orchard plot; D, UNIT annotated orchard plot; E, CAT caterpillar plot; F, UNIT caterpillar plot.

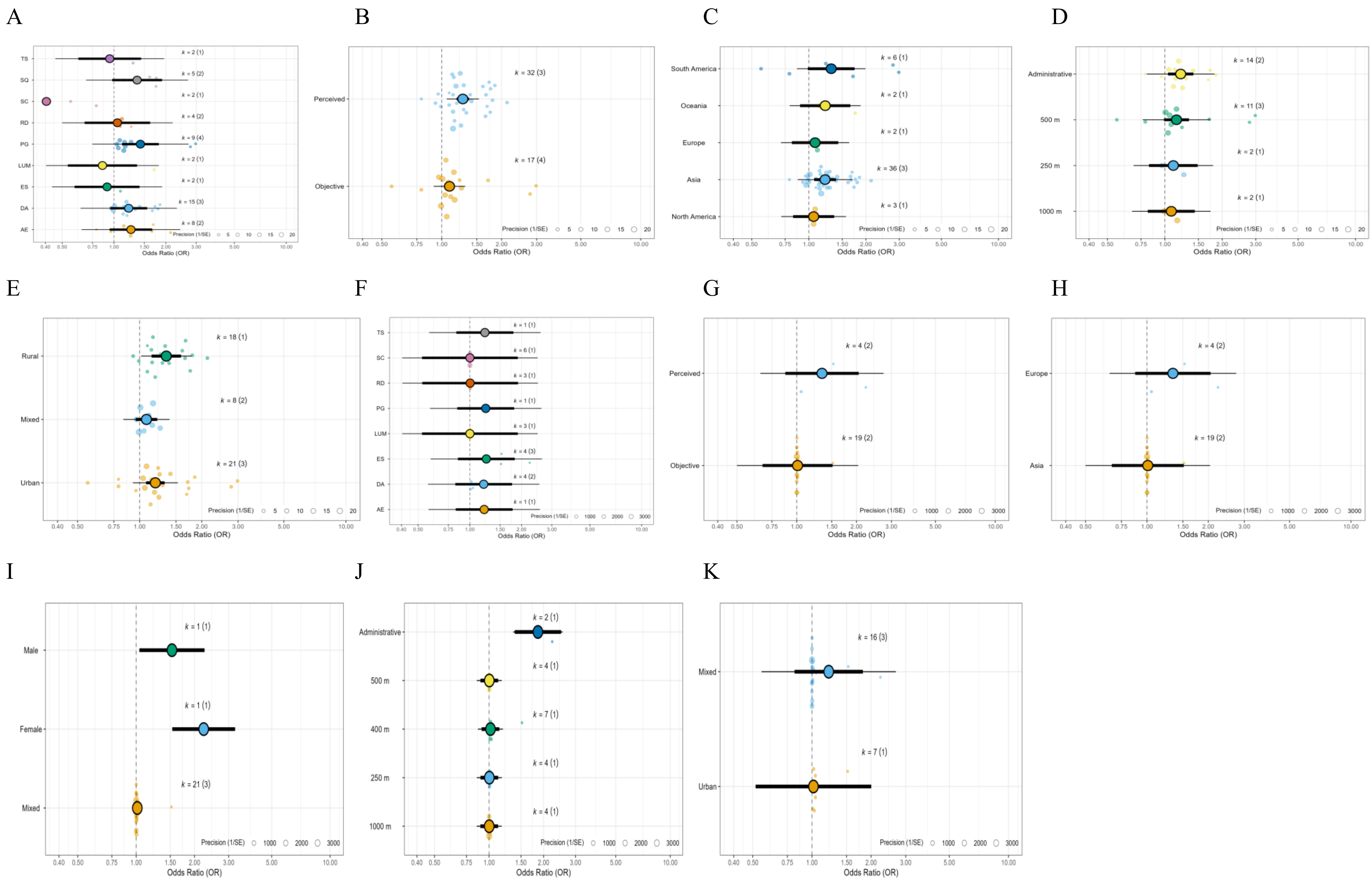

Supplementary Figure S16. General leisure-time physical activity (General LTPA): subgroup analyses. A, CAT by built-environment domain; B, CAT by environment-measure type; C, CAT by region; D, CAT by spatial scale; E, CAT by urbanicity; F, UNIT by built-environment domain; G, UNIT by environment-measure type; H, UNIT by region; I, UNIT by sex composition; J, UNIT by spatial scale; K, UNIT by urbanicity.

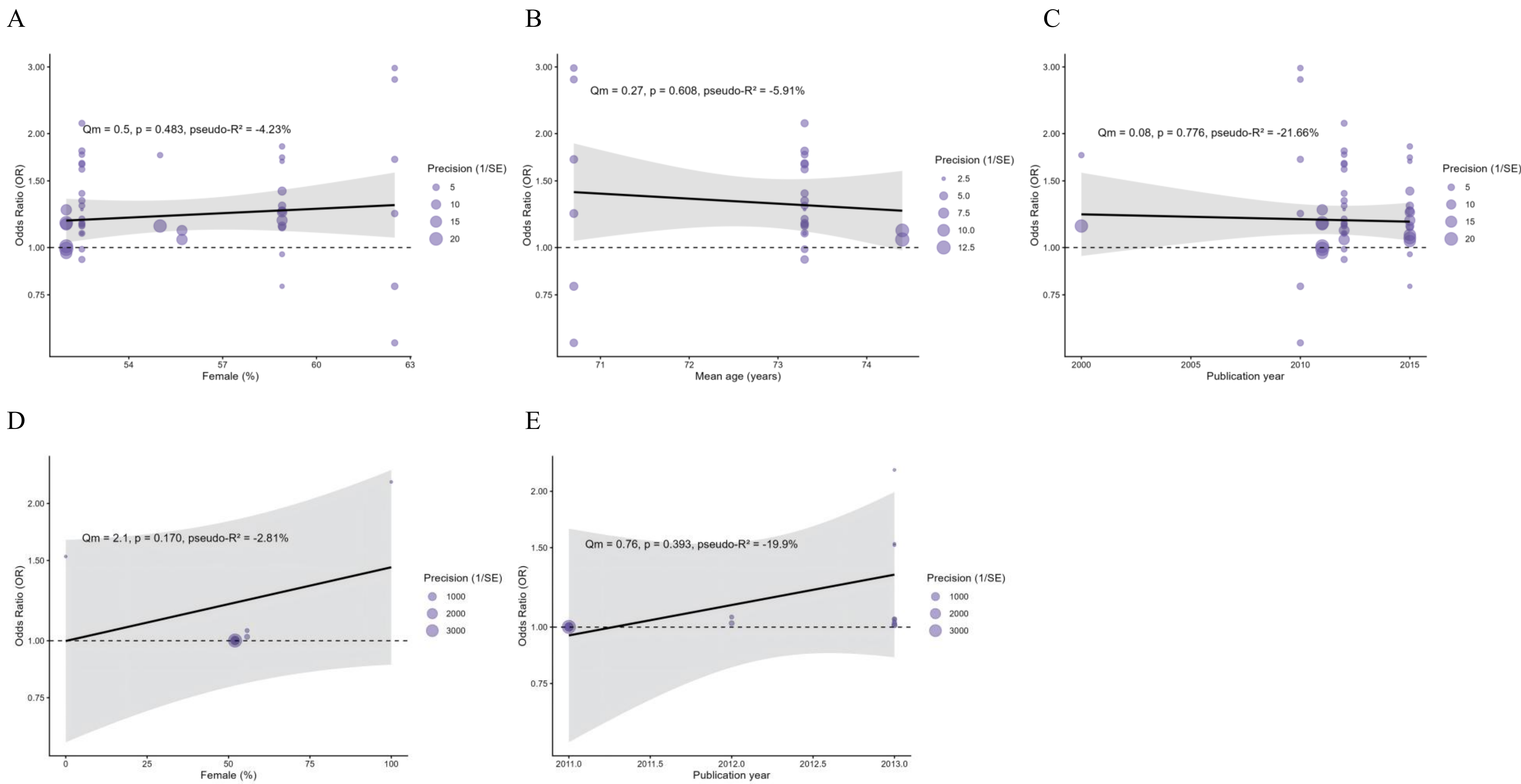

Supplementary Figure S17. General leisure-time physical activity (General LTPA): meta-regression plots. A, CAT by female percentage; B, CAT by mean age; C, CAT by publication year; D, UNIT by female percentage; E, UNIT by publication year.

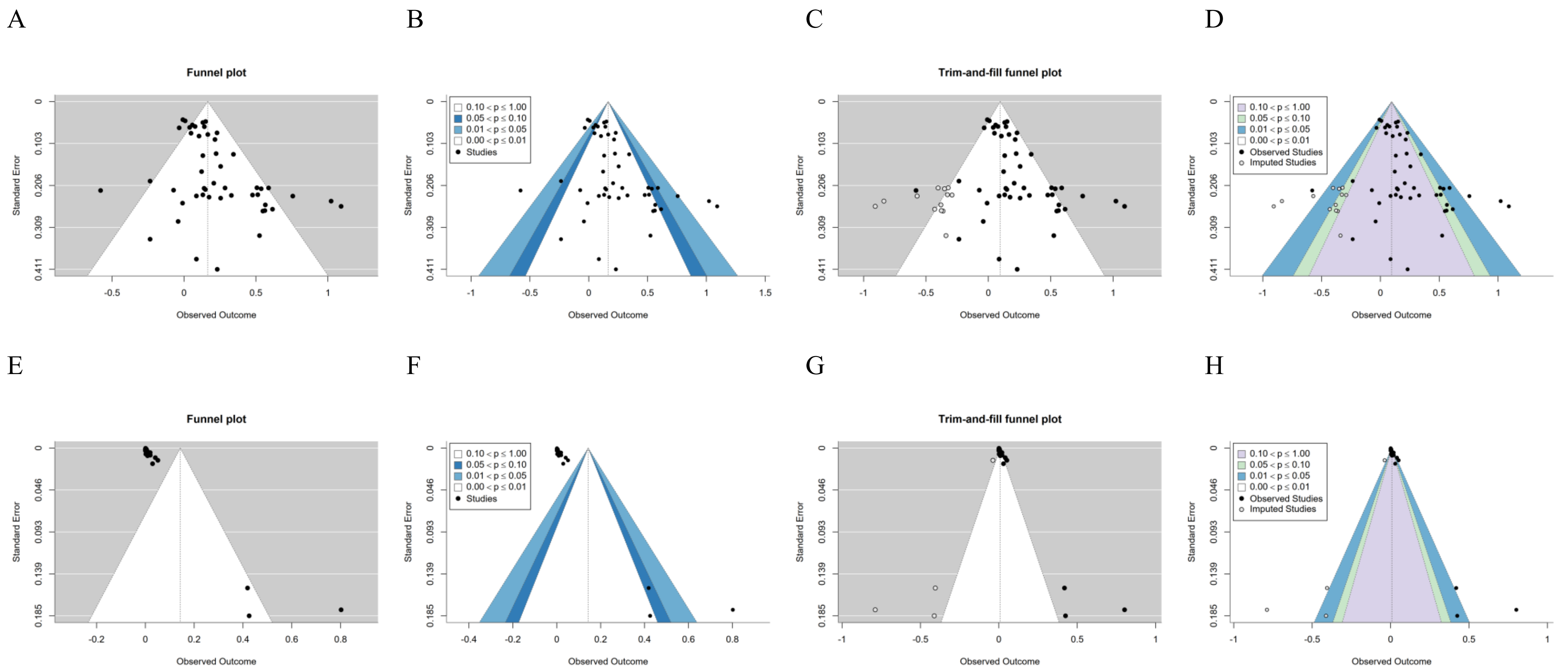

Supplementary Figure S18. General leisure-time physical activity (General LTPA): publication-bias and small-study-effect diagnostics. A, CAT basic funnel plot; B, CAT contour-enhanced funnel plot; C, CAT trim-and-fill basic funnel plot; D, CAT trim-and-fill contour-enhanced funnel plot; E, UNIT basic funnel plot; F, UNIT contour-enhanced funnel plot; G, UNIT trim-and-fill basic funnel plot; H, UNIT trim-and-fill contour-enhanced funnel plot.

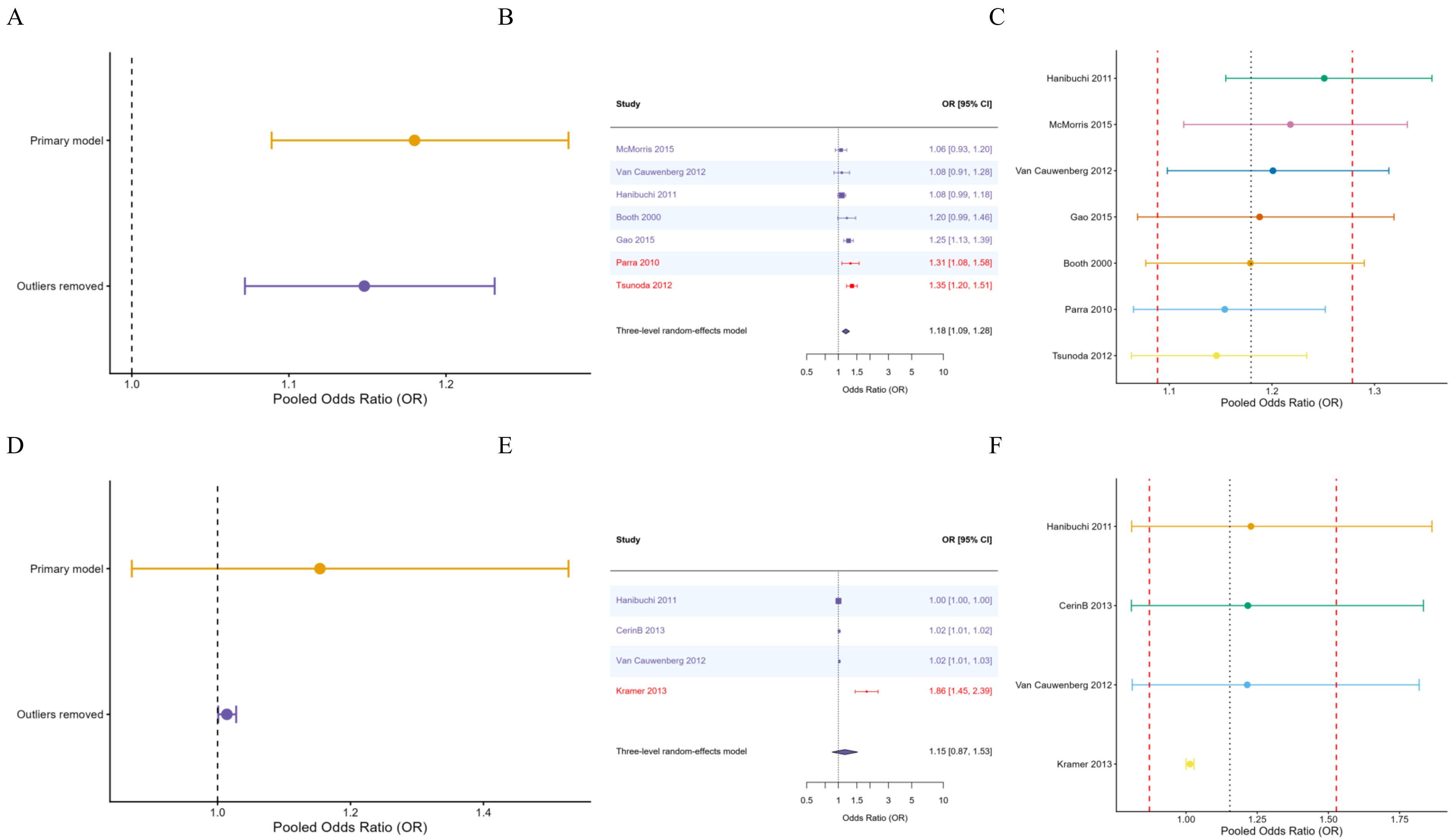

Supplementary Figure S19. General leisure-time physical activity (General LTPA): sensitivity analyses. A, CAT comparison before and after outlier removal; B, CAT outlier-identification forest plot; C, CAT leave-one-study/sample-out analysis; D, UNIT comparison before and after outlier removal; E, UNIT outlier-identification forest plot; F, UNIT leave-one-study/sample-out analysis.

A

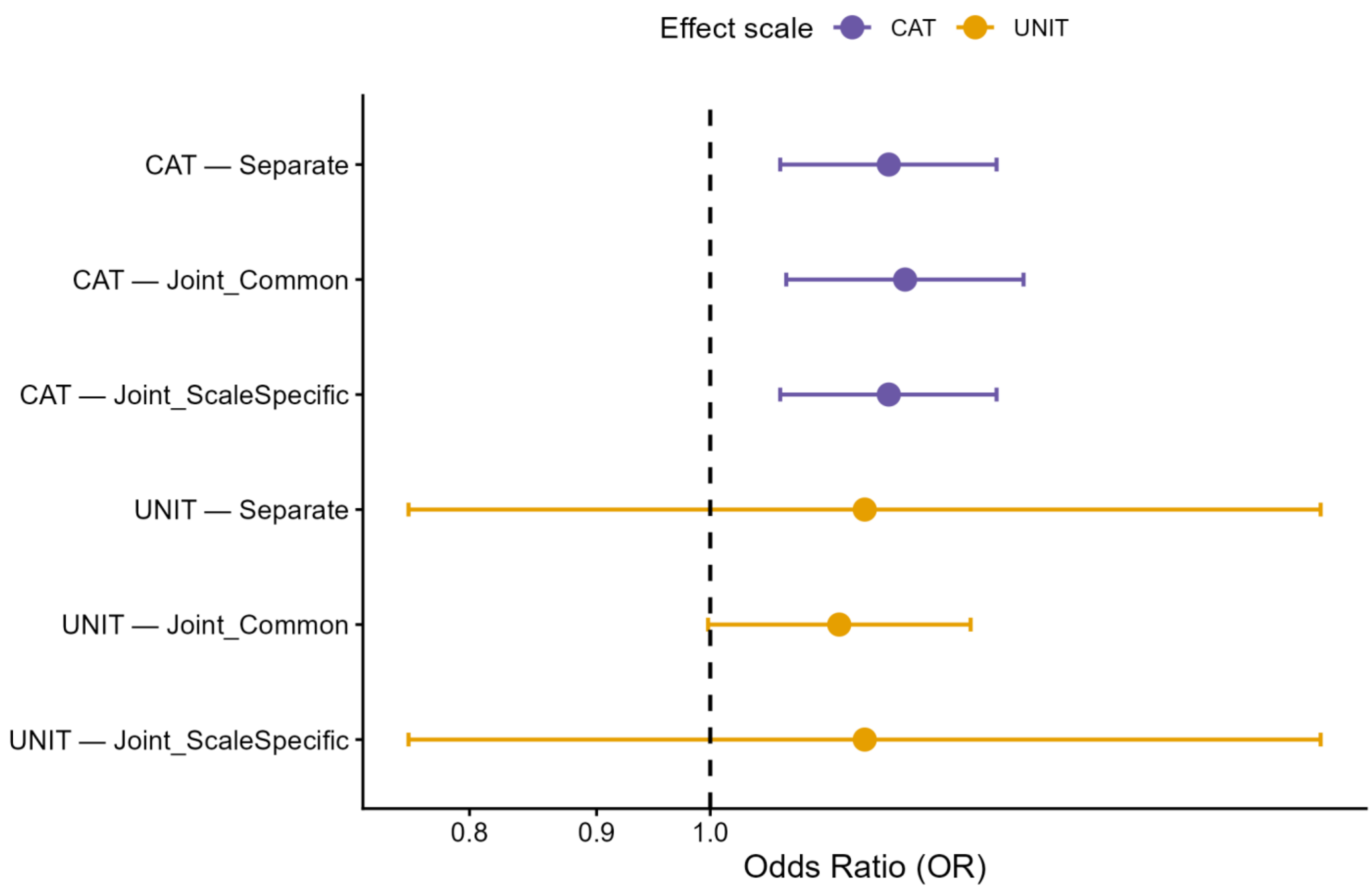

Supplementary Figure S20. General leisure-time physical activity (General LTPA): CAT–UNIT joint sensitivity analysis. A, comparison of separate and joint CR2 estimates.
